# Publisher Correction: Ecosystem productivity affected the spatiotemporal disappearance of Neanderthals in Iberia

**DOI:** 10.1038/s41559-022-01917-6

**Published:** 2022-10-21

**Authors:** M. Vidal-Cordasco, D. Ocio, T. Hickler, A. B. Marín-Arroyo

**Affiliations:** 1 Grupo I+D+i EvoAdapta (Evolución Humana y Adaptaciones Económicas y Ecológicas durante la Prehistoria), Departamento Ciencias Históricas, Universidad de Cantabria, Santander, Spain; 2 Mott MacDonald, Cambridge, UK; 3 Senckenberg Biodiversity and Climate Research Centre (SBiK-F), Frankfurt am Main, Germany; 4 Department of Physical Geography, Goethe University, Frankfurt, Germany

**Keywords:** Archaeology, Ecological modelling

Correction to: *Nature Ecology & Evolution* 10.1038/s41559-022-01861-5, published online 29 September 2022.

In the version of this article initially published, there was an error in equation (12), which originally read, in part, “$B_i = D_i{\int} {XW_i} \to$”. The equation has been corrected to read “$B_i = D_i \times W_i \to$” in the HTML and PDF versions of the article.

