# Supplementary material for: Ecosystem productivity affected the spatiotemporal disappearance of Neanderthals in Iberia
Source: Nat Ecol Evol. 2022 Sep 29;6(11):1644–57. doi: 10.1038/s41559-022-01861-5 (PMC9630105; doi:10.1038/s41559-022-01861-5)
Supplement: Supplementary file 1 — Supplementary Tables 1–7, Figs. 1–12 and Note. [file 41559_2022_1861_MOESM1_ESM.pdf]

nature portfolio

https://doi.org/10.1038/s41559-022-01861-5

Supplementary information

Ecosystem productivity affected the spatiotemporal disappearance of Neanderthals in Iberia

In the format provided by the authors and unedited

Supplementary Information

	Cluster 1		Cluster 2		Cluster 3	
	n	n	n	n	n	%
Eurosiberian (n=25)	16	64	8	32	1	4
Submediterranean (n=9)	4	44	3	33	2	23
Mesomediterranean (n=19)	1	5	11	58	7	37
Thermomediterranean (n=15)	1	6	3	20	11	74

Supplementary Table 1. Number (n) and percentage (%) of sites from the current Eurosiberian, Submediterranean, Mesomediterranean and Thermomediterranean regions which NPP estimations are grouped into the first, second and third clusters based on the *dCORT* dissimilarity index.

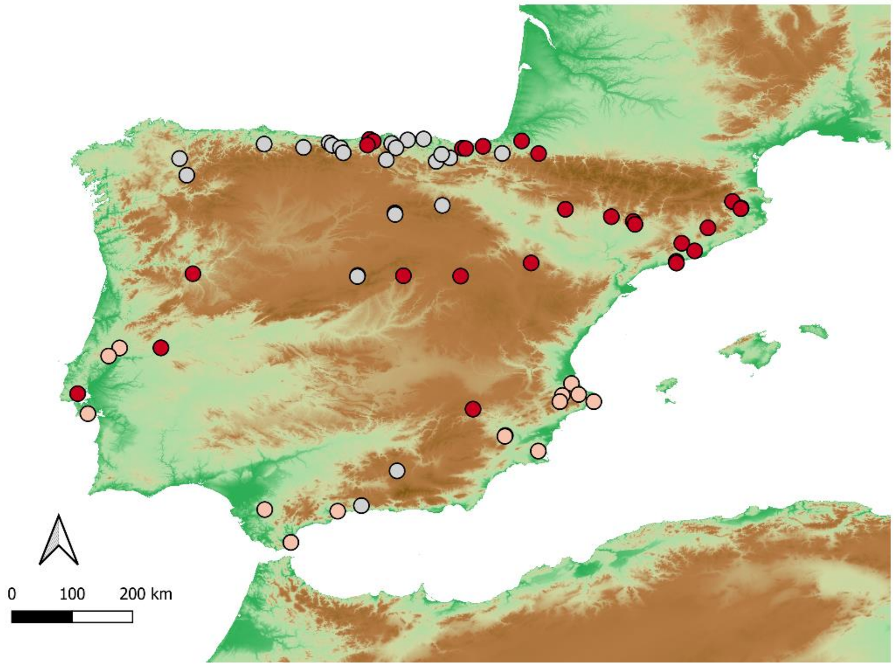

Supplementary Fig. 1. Geographic distribution of the archaeo-paleontological sites, which NPP estimations were clustered into the first (grey dots), second (red dots) and third (orange dots) groups, according to the *dCORT* dissimilarity index.

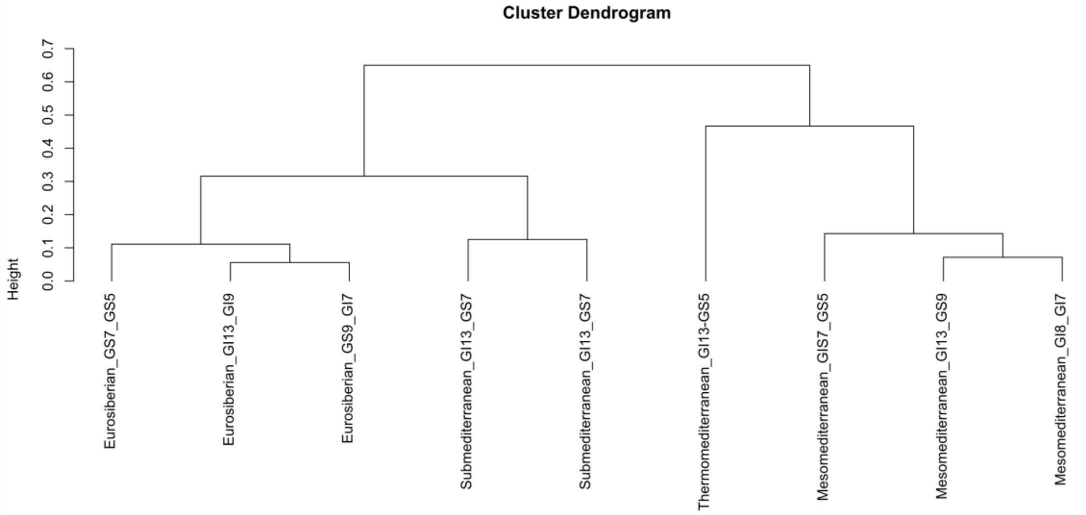

Supplementary Fig. 2. Cluster dendrogram showing the classification of each herbivore PCOM in each region according to the Jaccard Similarity Index. Each PCOM is defined by the list of herbivore species present in that region, during a certain period of time, expressed in stadial (GS) or interstadials (GI). Further information is available in the main text and in the Supplementary Dataset 2.

Region	Stadial-Interstadial	Small			Medium			Medium-large			Large		
		Mean	Min	Max	Mean	Min	Max	Mean	Min	Max	Mean	Min	Max
Eurosiberian	GI-13	13.69	10.66	17.58	168.00	130.83	215.75	153.48	119.52	197.10	427.46	332.87	548.93
Eurosiberian	GS-13	12.66	9.93	16.13	155.40	121.94	198.04	141.97	111.40	180.92	395.39	310.25	503.89
Eurosiberian	GI-12	12.39	9.74	15.76	152.10	119.58	193.46	138.95	109.24	176.74	386.99	304.25	492.23
Eurosiberian	GS-12	6.95	5.59	8.63	85.29	68.66	105.95	77.92	62.72	96.79	217.00	174.69	269.57
Eurosiberian	GI-11	15.50	11.91	20.18	190.30	146.16	247.76	173.85	133.53	226.34	484.18	371.89	630.38
Eurosiberian	GS-11	12.98	10.16	16.58	159.36	124.75	203.57	145.58	113.96	185.97	405.46	317.40	517.95
Eurosiberian	GI-10	15.48	11.89	20.15	190.00	145.96	247.32	173.58	133.35	225.95	483.42	371.38	629.28
Eurosiberian	GS-10	12.02	9.48	15.25	147.57	116.33	187.20	134.81	106.27	171.02	375.47	295.97	476.31
Eurosiberian	GI-9	14.46	11.20	18.68	177.51	137.43	229.29	162.17	125.55	209.48	451.66	349.66	583.41
Eurosiberian	GS-9	11.14	8.79	14.11	136.75	107.95	173.23	162.73	128.46	206.14	347.94	274.67	440.76
Eurosiberian	GI-8	15.63	11.91	20.51	191.81	146.13	251.75	228.24	173.90	299.58	488.02	371.82	640.55
Eurosiberian	GS-8	12.82	9.99	16.46	157.41	122.65	202.02	187.32	145.95	240.40	400.51	312.07	514.01
Eurosiberian	GI-7	14.53	11.17	18.91	178.39	137.11	232.11	212.28	163.15	276.20	453.89	348.85	590.56
Eurosiberian	GS-7	8.44	6.79	10.49	103.62	83.39	128.75	123.30	99.24	153.20	214.53	172.66	266.56
Eurosiberian	GI-6	13.68	10.70	17.49	167.94	131.37	214.70	199.85	156.32	255.49	347.71	271.98	444.52
Eurosiberian	GS-6	4.88	3.85	6.18	59.91	47.29	75.90	71.29	56.27	90.32	124.04	97.90	157.15
Eurosiberian	GI-5	14.62	11.36	18.81	179.42	139.41	230.92	213.51	165.89	274.79	371.48	288.64	478.10
Eurosiberian	GS-5	10.86	8.65	13.62	133.25	106.18	167.21	158.56	126.35	198.98	275.87	219.83	346.20
Thermomediterranean	GI-13	55.93	44.48	70.33	179.93	143.09	226.25	235.72	187.46	296.40	125.16	99.53	157.37
Thermomediterranean	GS-13	51.50	41.16	64.44	165.67	132.41	207.29	217.04	173.47	271.56	115.24	92.10	144.19
Thermomediterranean	GI-12	44.88	36.07	55.84	144.37	116.04	179.63	189.14	152.01	235.33	100.42	80.71	124.95
Thermomediterranean	GS-12	43.60	35.07	54.21	140.25	112.80	174.37	183.74	147.78	228.44	97.56	78.46	121.29
Thermomediterranean	GI-11	56.73	45.08	71.41	182.51	145.00	229.71	239.10	189.96	300.94	126.95	100.86	159.78
Thermomediterranean	GS-11	57.22	45.44	72.07	184.08	146.16	231.83	241.15	191.48	303.71	128.04	101.67	161.26

Thermomediterranean	GI-10	54.34	43.30	68.20	174.81	139.28	219.40	229.01	182.46	287.42	121.59	96.88	152.61
Thermomediterranean	GS-10	54.45	43.38	68.35	175.17	139.55	219.88	229.48	182.82	288.06	121.84	97.07	152.95
Thermomediterranean	GI-9	52.90	42.22	66.29	170.18	135.81	213.24	222.94	177.92	279.36	118.37	94.47	148.33
Thermomediterranean	GS-9	55.11	43.87	69.23	177.27	141.12	222.69	232.24	184.87	291.74	123.31	98.16	154.90
Thermomediterranean	GI-8	52.17	41.67	65.32	167.83	134.04	210.14	219.87	175.60	275.29	116.74	93.24	146.17
Thermomediterranean	GS-8	53.15	42.41	66.62	170.99	136.42	214.32	224.01	178.72	280.78	118.94	94.89	149.08
Thermomediterranean	GI-7	52.82	42.15	66.18	169.91	135.60	212.88	222.59	177.65	278.89	118.18	94.32	148.08
Thermomediterranean	GS-7	49.39	39.56	61.67	158.89	127.26	198.40	208.16	166.71	259.91	110.52	88.52	138.00
Thermomediterranean	GI-6	50.58	40.47	63.23	162.72	130.17	203.41	213.17	170.53	266.47	113.18	90.54	141.48
Thermomediterranean	GS-6	47.48	38.09	59.18	152.72	122.52	190.37	200.08	160.50	249.40	106.23	85.22	132.42
Thermomediterranean	GI-5	57.89	45.92	72.96	186.21	147.73	234.71	243.94	193.54	307.48	129.52	102.76	163.26
Thermomediterranean	GS-5	51.33	41.04	64.22	165.13	132.00	206.58	216.34	172.93	270.63	114.86	91.82	143.69
Mesomediterranean	GI-13	30.11	23.61	38.42	153.73	120.50	196.12	174.67	136.92	222.84	354.90	278.18	452.76
Mesomediterranean	GS-13	30.29	23.73	38.67	154.63	121.14	197.38	175.69	137.64	224.27	356.97	279.66	455.66
Mesomediterranean	GI-12	27.44	21.69	34.73	140.09	110.70	177.27	159.17	125.79	201.42	323.41	255.57	409.25
Mesomediterranean	GS-12	24.08	19.20	30.19	122.91	98.03	154.11	139.66	111.38	175.11	283.75	226.31	355.78
Mesomediterranean	GI-11	32.21	25.08	41.36	164.41	128.02	211.14	186.80	145.46	239.90	379.55	295.54	487.42
Mesomediterranean	GS-11	29.72	23.33	37.87	151.73	119.08	193.33	172.40	135.30	219.67	350.28	274.90	446.33
Mesomediterranean	GI-10	31.30	24.44	40.08	159.78	124.77	204.60	181.54	141.77	232.47	368.85	288.05	472.33
Mesomediterranean	GS-10	28.14	22.19	35.69	143.67	113.30	182.18	163.24	128.73	207.00	331.67	261.55	420.58
Mesomediterranean	GI-9	31.83	24.82	40.83	162.51	126.69	208.45	184.65	143.95	236.85	375.16	292.48	481.22
Mesomediterranean	GS-9	28.98	22.80	36.85	147.96	116.38	188.10	168.11	132.24	213.72	341.57	268.68	434.24
Mesomediterranean	GI-8	35.52	27.76	45.46	181.32	141.69	232.05	206.02	160.99	263.66	314.07	245.41	401.93
Mesomediterranean	GS-8	34.46	27.01	43.98	175.93	137.87	224.49	199.90	156.66	255.07	304.73	238.81	388.84
Mesomediterranean	GI-7	35.26	27.57	45.08	179.97	140.73	230.15	204.49	159.90	261.50	311.73	243.76	398.64
Mesomediterranean	GS-7	34.59	27.46	43.58	176.58	140.15	222.46	200.63	159.25	252.77	201.77	160.15	254.20

Mesomediterranean	GI-6	41.00	32.08	52.40	209.29	163.75	267.50	237.80	186.06	303.94	239.15	187.11	305.66
Mesomediterranean	GS-6	34.23	27.19	43.09	174.72	138.79	219.96	198.53	157.69	249.93	199.65	158.59	251.34
Mesomediterranean	GI-5	44.23	34.34	56.97	225.81	175.32	290.84	256.57	199.20	330.46	258.02	200.33	332.33
Mesomediterranean	GS-5	40.67	31.85	51.94	207.62	162.57	265.16	235.91	184.72	301.28	237.24	185.76	302.99
Submediterranean	GI-13	37.80	29.91	47.77	134.82	106.68	170.39	147.51	116.72	186.42	319.38	252.72	403.63
Submediterranean	GS-13	31.10	24.91	38.83	110.91	88.83	138.47	121.35	97.19	151.50	262.74	210.44	328.03
Submediterranean	GI-12	33.20	26.50	41.59	118.42	94.53	148.34	129.56	103.42	162.30	280.52	223.93	351.41
Submediterranean	GS-12	17.81	14.21	22.33	63.53	50.67	79.65	69.51	55.44	87.14	150.50	120.04	188.68
Submediterranean	GI-11	36.56	29.00	46.09	130.39	103.43	164.37	142.66	113.17	179.83	308.88	245.03	389.37
Submediterranean	GS-11	27.34	22.00	33.99	97.52	78.45	121.24	106.70	85.83	132.64	231.02	185.84	287.20
Submediterranean	GI-10	37.03	29.35	46.73	132.08	104.68	166.66	144.51	114.52	182.34	312.88	247.97	394.80
Submediterranean	GS-10	23.81	19.18	29.57	84.93	68.39	105.47	92.92	74.83	115.39	201.19	162.02	249.84
Submediterranean	GI-9	31.62	25.31	39.51	112.77	90.25	140.91	123.38	98.75	154.17	267.15	213.81	333.80
Submediterranean	GS-9	21.97	17.67	27.31	78.35	63.03	97.40	85.72	68.96	106.56	185.60	149.30	230.72
Submediterranean	GI-8	35.38	28.13	44.50	126.19	100.33	158.71	138.06	109.77	173.64	298.93	237.68	375.97
Submediterranean	GS-8	25.34	20.41	31.47	90.37	72.78	112.23	98.88	79.62	122.79	214.09	172.40	265.86
Submediterranean	GI-7	34.22	27.26	42.94	122.03	97.24	153.14	133.51	106.39	167.55	289.08	230.35	362.78
Submediterranean	GS-7	16.84	13.39	21.19	60.07	47.74	75.58	65.72	52.24	82.70	142.31	113.10	179.05
Submediterranean	GI-6	32.15	25.89	39.92	114.66	92.34	142.38	125.45	101.03	155.77	142.90	115.08	177.44
Submediterranean	GS-6	17.32	13.48	22.26	61.78	48.08	79.38	67.59	52.60	86.85	76.99	59.91	98.93
Submediterranean	GI-5	39.63	31.78	49.42	141.34	113.34	176.26	154.64	124.00	192.84	176.14	141.25	219.66
Submediterranean	GS-5	31.20	25.12	38.74	111.26	89.60	138.17	121.73	98.03	151.17	138.66	111.66	172.19

Supplementary Table 2. Estimated biomass of small (<10 kg), medium (10-100 kg), medium-large (100-500 kg), and large (>500 kg) sized herbivores in each Greenland Stadial (GS) and Interstadial (GI) period in the Eurosiberian, Submediterranean, Thermomediterranean and Mesomediterranean regions. It can be observed the mean value and the 95% Confidence Interval for the estimations (min and max).

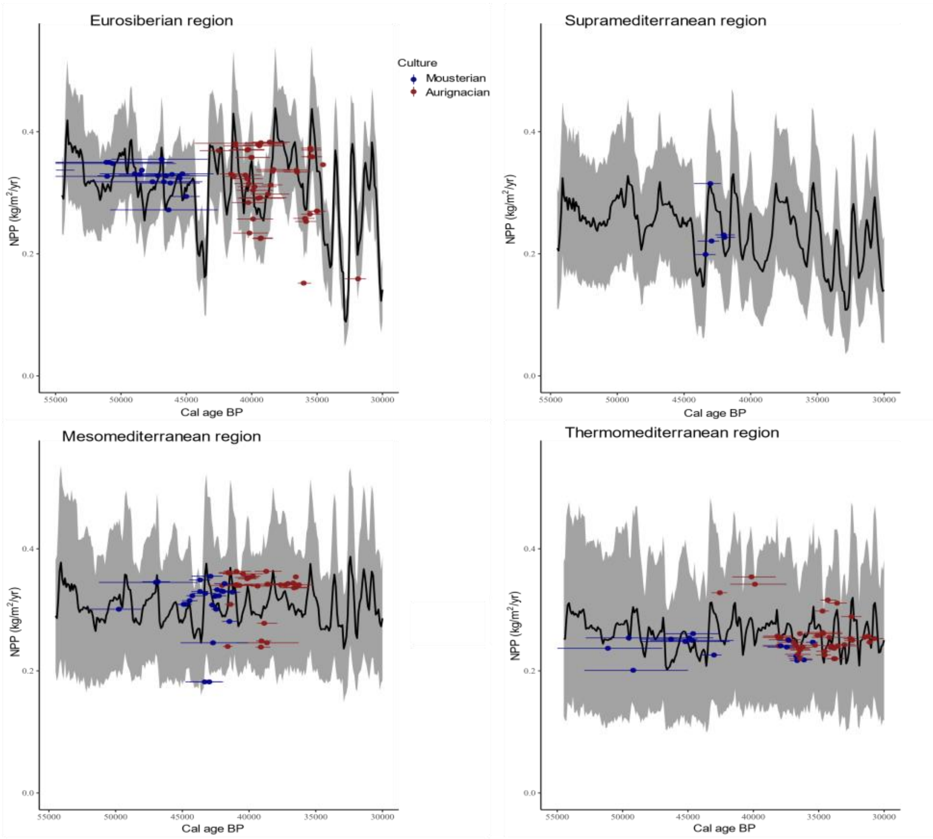

Supplementary Fig. 3. Temporal evolution of the Net Primary Productivity (NPP) in each biogeographic region of Iberia between 54.5 and 30 ky BP. Dots represent the mean values and the horizontal bars the 95% CI of each Mousterian (blue) and Aurignacian (red) raw calibrated date.

a.

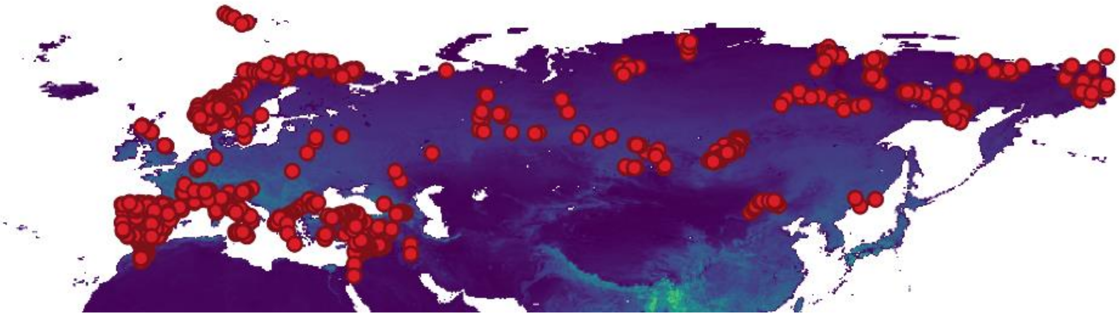

b.

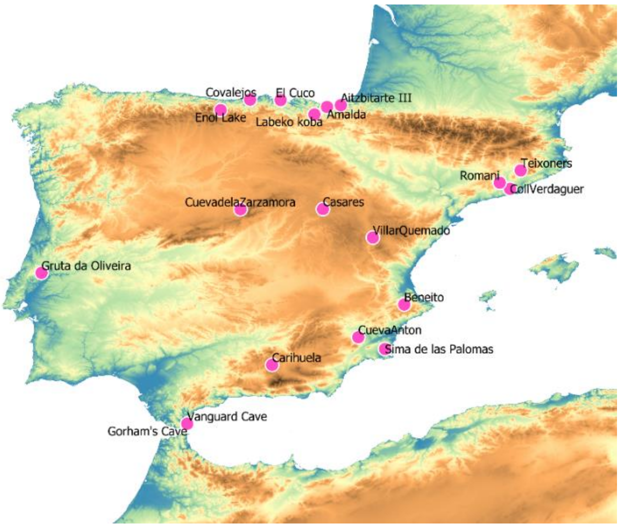

Supplementary Fig. 4. a. Localities of Europe and Asia with extant pollen taxa percentages and its associated climatological conditions from the European Modern Pollen Database[1] b. Archaeological sites with dated pollen samples used to estimate mean annual temperature and mean annual precipitation in the paleoclimate validation process. For details, see Supplementary Dataset 2.

Mean annual temperature (C°)					Mean Annual Precipitation (mm/year)				
RMSE	r^2	p	Avg. Bias	Max. Bias	RMSE	r^2	p	Avg. Bias	Max. Bias
4.14	0.77	0.0001	0.003	8.26	294.4	0.50	0.001	-2.4	1967.5

Supplementary Table 3. Results obtained from a two-sided Weighted Averaging regression test after bootstrap. Values of Root Mean Square Error of Prediction (RSEMP), correlation coefficient (r^2), p-values (p), average bias of the model residuals and maximum bias of the model residuals after the bootstrap cross-validation process.

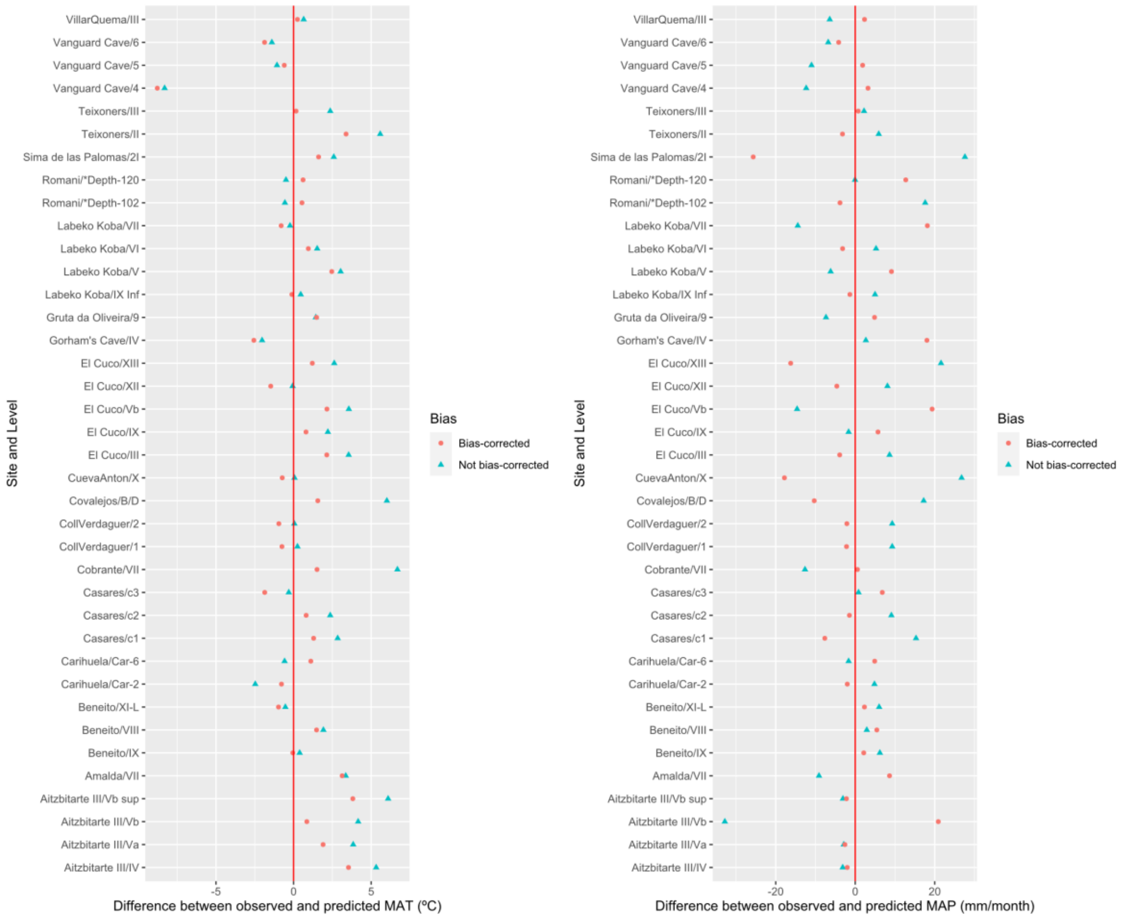

Supplementary Fig. 5. Difference in the MAT and MAP values obtained from the HadCM3B-M2.1 coupled general circulation model[2] and that obtained from the palynological record before (green triangle) and after (red circle) using the delta correction method. The vertical red line indicates when the difference between the observed and predicted values is 0.

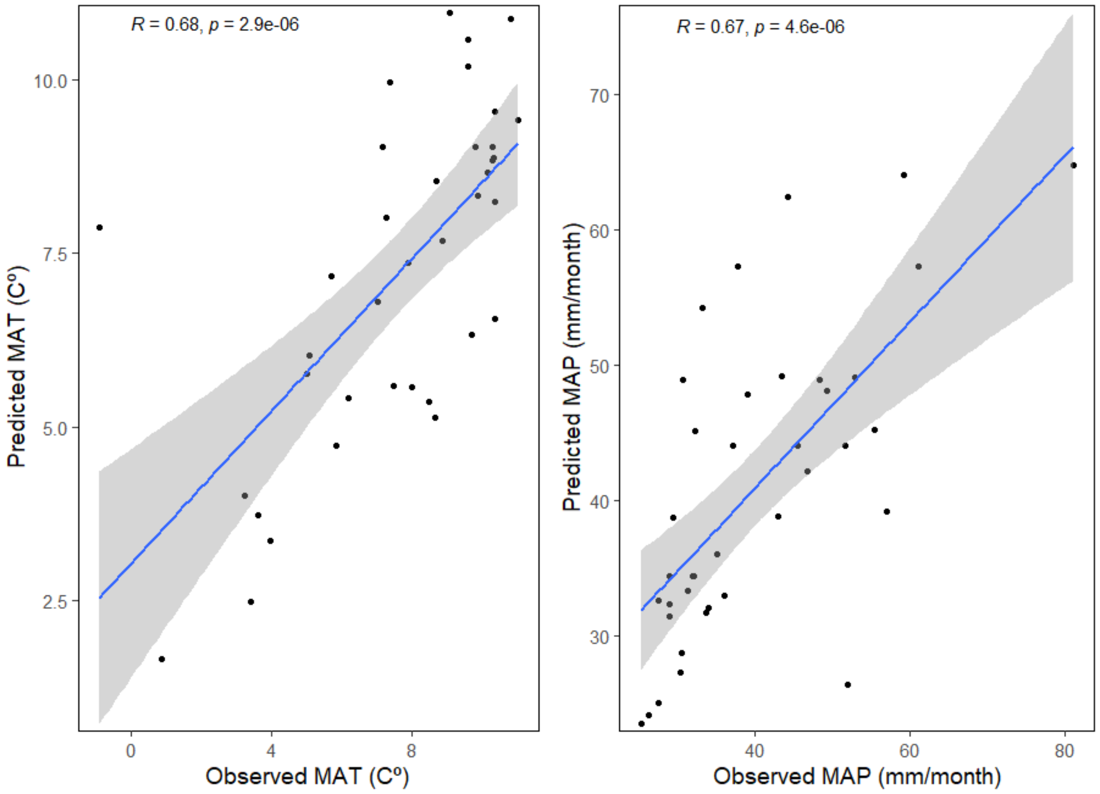

Supplementary Figure 6. Correlation between the estimated mean annual temperatures (observed MAT) and precipitation (observed MAP) from the palynological record and the values obtained from the HadCM3B-M2.1 coupled general circulation[2]. Each dot represents the mean value and in shaded grey the 95% CI of the regression model. The Pearson's correlation coefficient (P) and significance level (p-value) is provided.

Parameter	Value	Parameter	Value
vegmode	“cohort”	ifdisturb	1
nyear	25000	distinterval	200
Nyear_spinup	500	ifcalccton	1
ifdailynpp	1	ifbgestab	1
ifdailydecomp	0	ifsme	1
ifcalcsla	1	ifstochestab	1
iffire	0	ifstochmort	1
npatch	100	ifcdebt	0
patcharea	1000	textured_soil	1
estinterval	5		

Supplementary Table 4. Global parameters used in the LPJ-GUESS model. Vegmode= vegetation model mode, nyear= number of simulated years, nyear_spinup= duration of the spin-up phase in years, ifdailynpp= calculate NPP daily (1) or monthly (0), ifdailydecomp= calculate soil respiration daily (1) or monthly (0), eifcalcsla= calculate SLA from leaf longevity (1) or not (0), iffire= implement fire (1) or not (0), npatch= number of replicate patches, patcharea= patch area in m^2, estinterval= years between establishment events in cohort mode, ifdisturb= generic patch-destroying disturbaces enabled (1) or not (0), destinterval= average return time for generic patch-destroying disturbances in years, ifcalccton= whether to calculate leaf C:N min from leaf longevity (1) or not (0), ifbgestab= background establishment enabled (1) or not (0), ifsme= spatial mass effect enabled (1) or not (0), ifstochestab= establishment stochastic (1) or not (0), ifstochmort= mortality stochastic (1) or not (0), ifcdebt= C debt permitted (1) or not (0), textured_soil = if soil class input is used (1) or not (0)

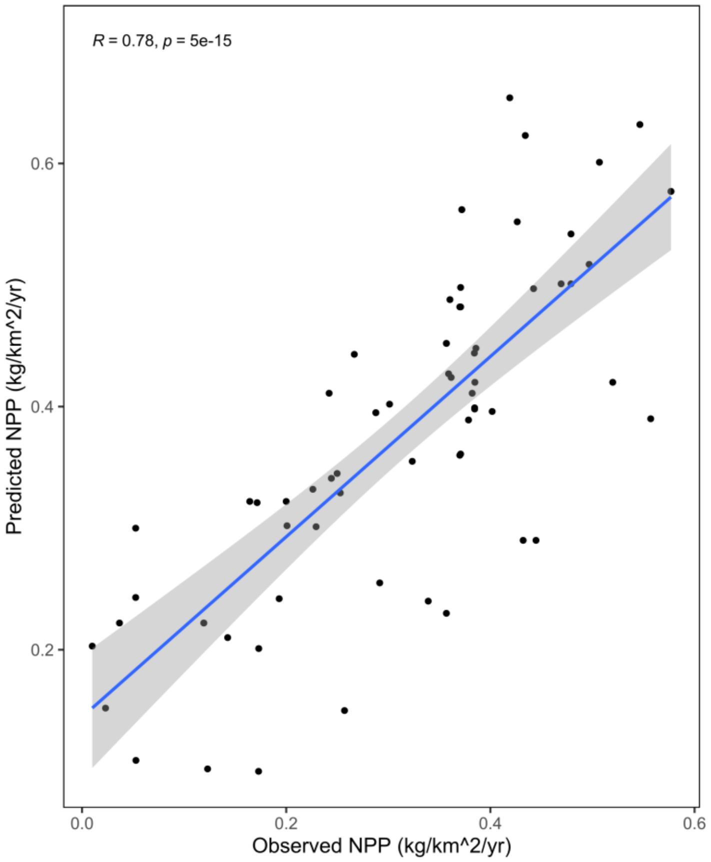

Supplementary Fig. 7. Correlation between the observed and predicted values of Net Primary Productivity (NPP) for the present-day. The mean values are represented with black dots and the 95% CI of the regression model in shaded grey. The Pearson's correlation coefficient (P) and significance level (p-value) is provided. Observed values were obtained from Imhoff et al.[3]

		Model 1		
Region	Mean	SD	Min	Max
EuroSiberian	0.30	0.07	0.12	0.44
Mesomediterranean	0.30	0.03	0.25	0.37
Submediterranean	0.24	0.05	0.16	0.33
Thermomediterranean	0.25	0.02	0.20	0.31
		Model 2		
Region	Mean	SD	Min	Max
EuroSiberian	0.24	0.07	0.10	0.40
Mesomediterranean	0.25	0.04	0.18	0.33
Submediterranean	0.19	0.05	0.10	0.32
Thermomediterranean	0.20	0.03	0.14	0.29
		Model 3		
Region	Mean	SD	Min	Max
EuroSiberian	0.35	0.08	0.14	0.50
Mesomediterranean	0.37	0.03	0.29	0.45
Submediterranean	0.26	0.06	0.14	0.37
Thermomediterranean	0.29	0.03	0.23	0.38

Supplementary Table 5. Mean value, Standard deviation (SD) and minimum and maximum values of the Net Primary Productivity (kg/km^2/yr) estimated for the GI-12, GS-12, GS-9, GI-8 and GS-5 with the LPJ-GUESS model when the input paleoclimate data of each archaeological and paleontological site is obtained from Armstrong et al.[2](Model 1), Beyer et al.[4] (Model 2), and Holden et al.[5] (Model 3).

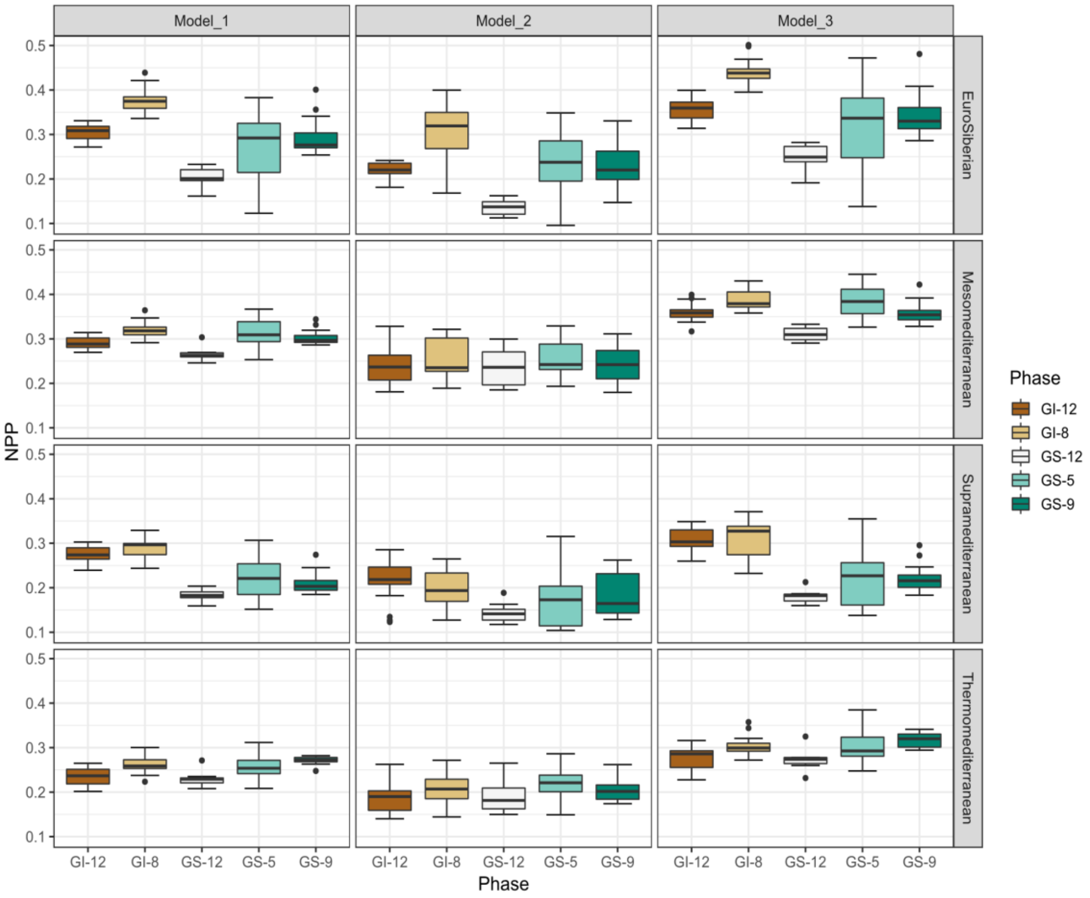

Supplementary Fig. 8. Box and whisker plot of the estimated NPP in each biogeographic region during the GI-12, GS-12, GS-9, GI-8 and GS-5 when the input paleoclimate data is obtained from Armstrong et al.[2](Model 1), Beyer et al.[4] (Model 2), and Holden et al.[5] (Model 3). The centre line of the box-plot elements corresponds to the median, the box limits to the first and third quartiles, the whiskers to 1.5x interquartile range and the dots to the outliers.

a.

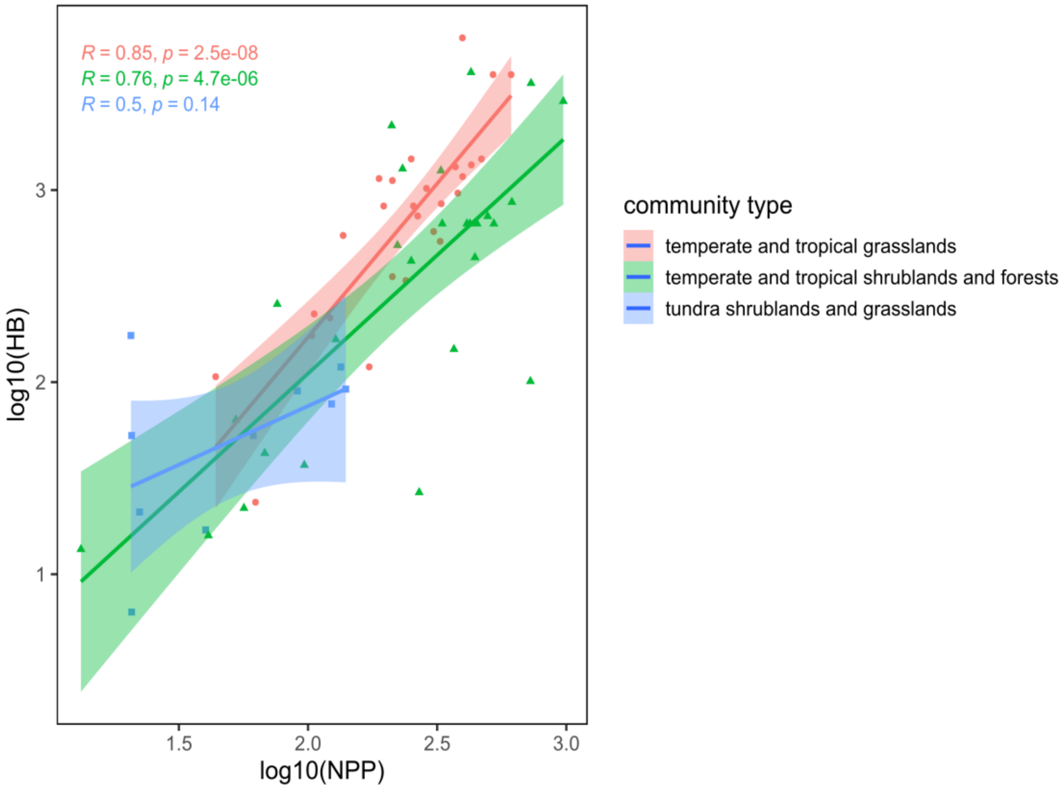

b.

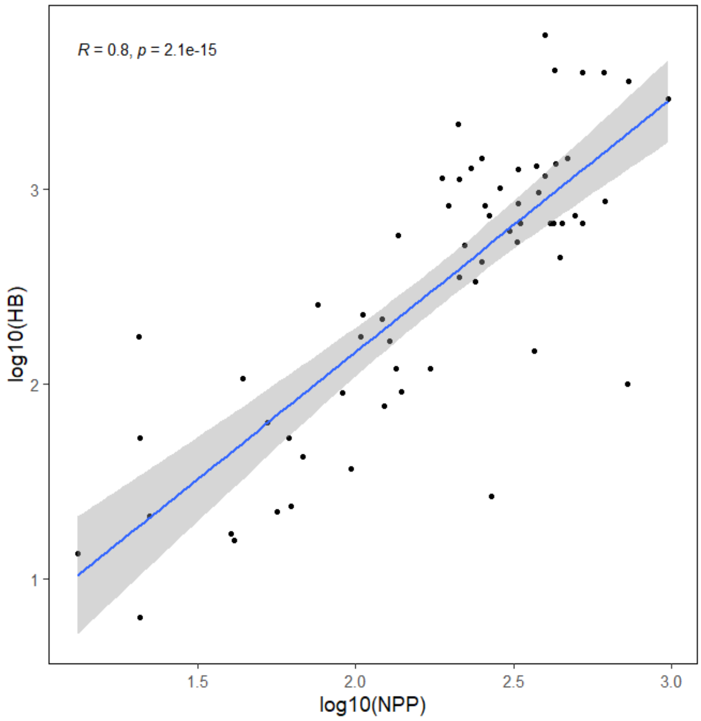

Supplementary Fig 9. a. Tow-sided Pearson's correlation coefficient test to assess the associations between the log-transformed Net Primary Productivity (NPP) and the log-transformed herbivore biomass (HB) in different plant community types. The 95% CI of the regression model is in shaded grey b. Tow-sided Pearson's correlation coefficient test to assess the associations between NPP and herbivore biomass across different terrestrial ecosystems. In shaded grey is represented the 95%CI of the regression model. The data used to compute these correlations is available in the Supplementary Dataset 3.

	Estimate	Std. Error	t-value	p-value	Adj. r^2
Intercept	-0.6422	0.3380	-1.900	-0.0621	0.701
log_{10}(NPP)	1.4017	0.1439	9.737	4.1×10^{-14}	

Supplementary Table 6. Results of the linear model to predict the herbivore biomass from the Net Primary Productivity. This statistical test used was a MM-type estimator for linear models because the outcomes obtained are more robust than least square regressions (the latter is more sensitive to outliers). Thus, in MM-type regressions is less likely that a small fraction of outliers have a large effect on the results obtained [6].

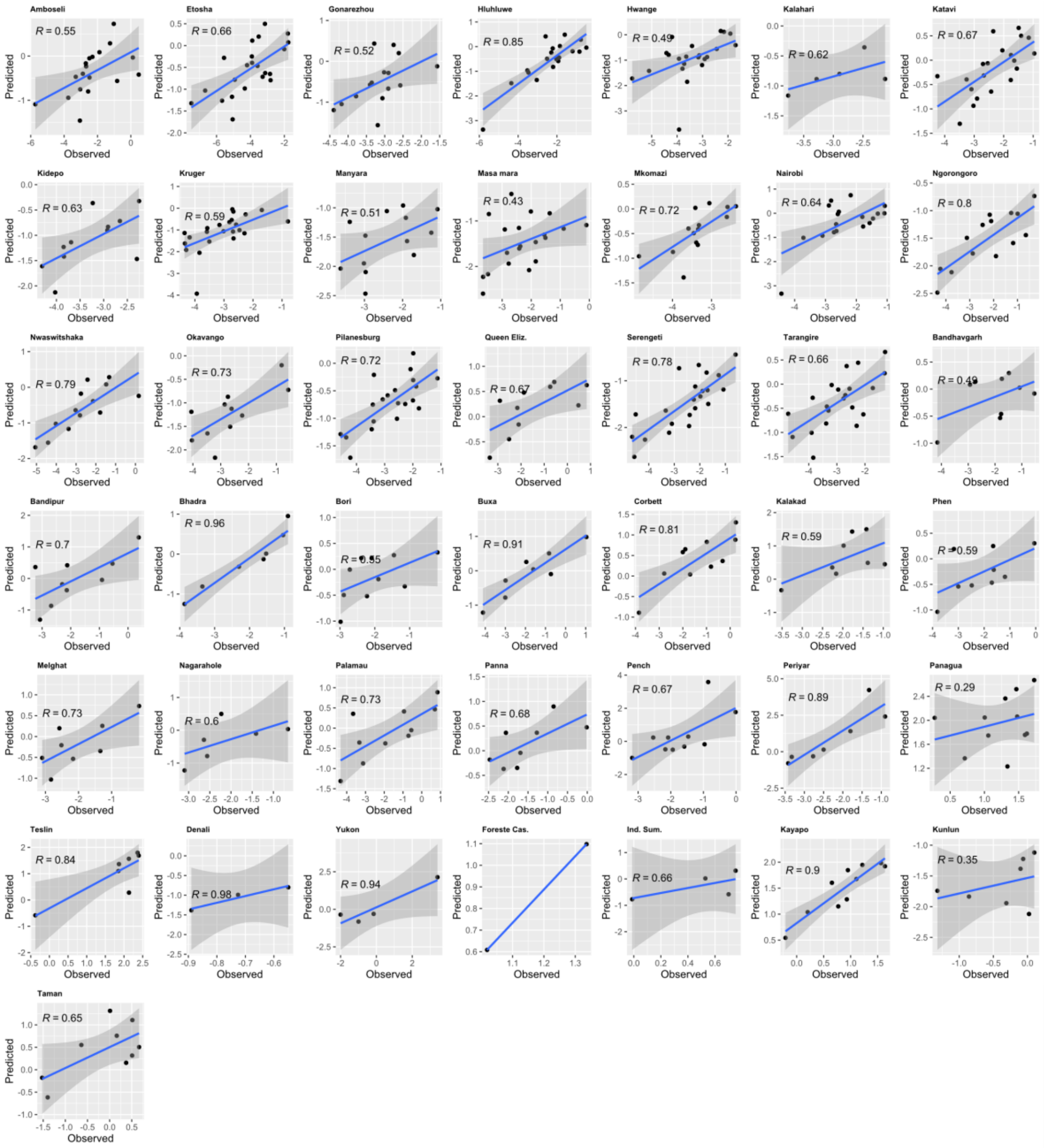

Supplementary Fig. 10. Linear correlation between the observed and predicted population density of extant herbivores species in different national parks/reserves. The mean values are represented with black dots and the 95% CI of the regression model in shaded grey. For details, see Supplementary Dataset 3.

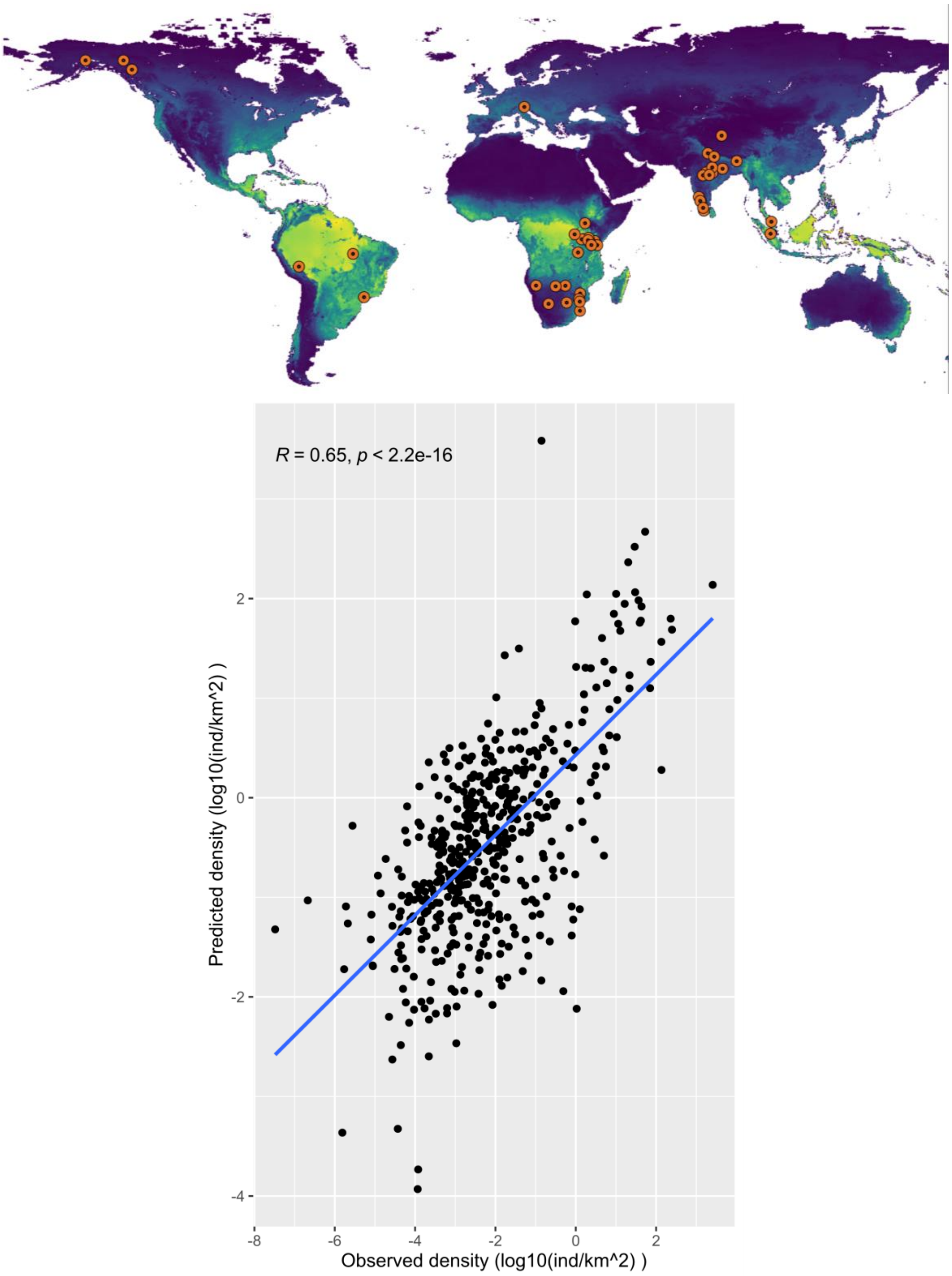

Supplementary Fig. 11. Geographical distribution of the national parks or reserves with empirical data on extant herbivore population densities used to validate the model. Below, two-sided Pearson's correlation coefficient test used to assess the relationship between the observed and predicted herbivore population densities across all the national parks/reserves. For details, see Supplementary Dataset 3.

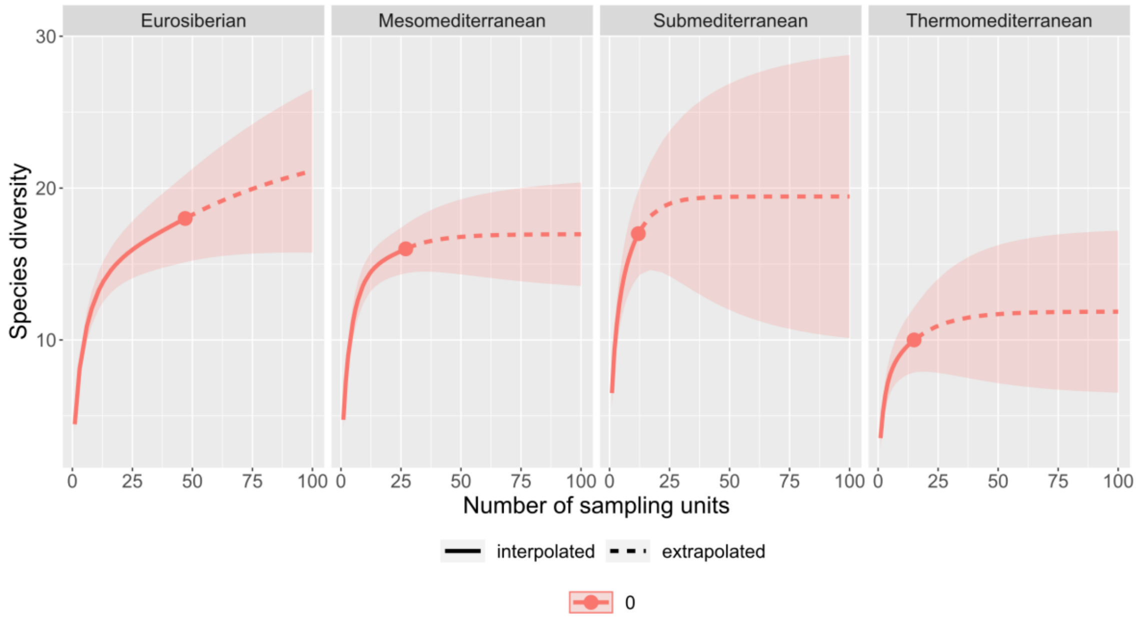

Supplementary Fig. 12 Species diversity in each biogeographic region (red dot) according to the LFAs used in the study and expected increase in the species diversity (discontinuous line) if the sample of LFAs was 100. The shaded part shows the 95%CI of the estimations.

Region	Observed Richness	Extrapolated Richness	%
Eurosiberian	18 (14.98-21.01)	21.13 (15.75-26.5)	14.8
Submediterranean	17 (14.40-19.59)	18.83 (10.70-26.96)	9.7
Mesomediterranean	16 (13.65-18.34)	18.77 (13.04-24.36)	14.7
Thermomediterranean	10 (7.87-12.12)	11.86(6.63-17.08)	15.6

Supplementary Table 7. Species richness in each biogeographic region with the actual sample size used in the study (Observed Richness) and the expected richness with a sample size of 100 LFAs (Extrapolated Richness). % indicates the difference between the observed and extrapolated richness in each region.

REFERENCES

1. Davis, B. A. S. *et al.* The Eurasian Modern Pollen Database (EMPD), version 2. *Earth Syst. Sci. Data* 12, 2423–2445 (2020).
2. Armstrong, E., Hopcroft, P. O. & Valdes, P. J. A simulated Northern Hemisphere terrestrial climate dataset for the past 60,000 years. *Sci. Data* 6, 1–16 (2019).
3. Imhoff, M. L. *et al.* Global patterns in human consumption of net primary production. *Nat. 2004 4296994* 429, 870–873 (2004).
4. Beyer, R. M., Krapp, M. & Manica, A. High-resolution terrestrial climate, bioclimate and vegetation for the last 120,000 years. *Sci. Data* 7, 1–9 (2020).
5. Holden, P. B. *et al.* PALEO-PGEM v1.0: A statistical emulator of Pliocene-Pleistocene climate. *Geosci. Model Dev.* 12, 5137–5155 (2019).
6. Kudraszow, N. L. & Maronna, R. A. Estimates of MM type for the multivariate linear model. *J. Multivar. Anal.* 102, 1280–1292 (2011).

Supplementary Note

REGIONAL BAYESIAN AGE MODELS

1. Eurosiberian Region 24
1.1. Excluded sites, levels or dates. 24
1.2. End Mousterian 26
1.3. Châtelperronian 41
1.4. Aurignacian 47
1.5. Summary 63
2. Mediterranean region 64
2.1. Excluded sites, levels or dates 64
2.2. Supramediterranean region: End Mousterian 67
2.2.1. Summary 74
2.3. Mesomediterranean 74
2.3.1. End Mousterian 75
2.3.2. Aurignacian 96
Summary 112
2.4. Thermomediterranean 113
2.4.1. End Mousterian 113
2.4.2. Châtelperronian 133
2.4.3. Aurignacian 136
Summary 160

1. Eurosiberian Region

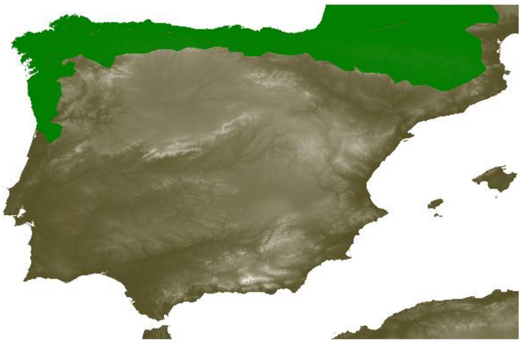

Figure 1. The Iberian Peninsula with the Eurosiberian biogeographic region shaded in green.

1.1. Excluded sites, levels or dates.

In the Eurosiberian region, the following archaeological levels were excluded from the Bayesian age models:

- Level N4. Prado Vargas.

In level N4, two radiocarbon dates were obtained from two charcoals with AMS: Beta-548,573 (38900±380 uncal BP) and Beta-548,572 (36830±330 uncal BP). These radiocarbon dates significantly differ from the Optically Stimulated Luminescence (OSL) dates performed on quartz grains obtained from the same level, which provided three different dates: 54.6±3.2, 46.9±4.2; and 48.3±3.2 ka BP. Furthermore, a chronological assessment of this level with amino acid racemisation (AAR) performed on a horse tooth (sample BSL 4682) yield a chronology of 46.4 ka BP. These results show that Mousterian levels were deposited during the MIS 3, but it is not possible to provide a more precise chronology. In this connection, the researcher who studied this level consider that "*given the close agreement between the mean OSL ages (46.2 ± 3.2 and 48.3 ± 3.2 ka BP at 1σ) and the mean AAR age for Level N4 (46.4 ka BP), the systematic 14C age offset could be indicative of minor problems related to incomplete removal of organic contaminants*" [1].

- Level 3. Cova Eirós.

It has been recently published a radiocarbon date (OxA-30471) performed with AMS on a bone remain recovered from the top of level 3 in Cova Eiríos, providing a chronology of 35,100 ± 700 BP (uncalibrated) [2]. The authors claimed that this chronology is parsimonious with the paleoenvironmental reconstructions previously made from the micromammal remains recovered from this level [3]. However, they also acknowledge that "*dating of Level 3 is problematic due to the discrepancy observed between the first OSL dates and subsequent results of the paleoenvironmental and sedimentological studies. Initially, for Level 3 two dates were obtained by OSL at the Universidad*

Autónoma de Madrid (UAM): 87,540 ± 6112 yrs BP (MAD-5951BIN) and 84,807 ± 4919 yrs BP (MAD-5612BIN). Therefore, the Neanderthal occupations of Level 3 would be placed at the MIS 5b interstadial” [2]. These discrepancies cast doubts about the chronology of this occupation and, as the authors acknowledge, more radiocarbon and OSL dates are necessary to better define the chronology of this site.

- Level IV, A Valiña.

Level IV in A Valiña was excavated in 1987 and two radiocarbon dates (GrN-17729 and GrN-20833) were obtained from bone remains, providing a chronology of 34,800±1,900 and 31,730±1,800 BP respectively (uncalibrated). The cultural attribution of this archaeological level was based on a Châtelperronian point and a relatively high proportion of burins in the assemblage; however, these lithic tools were posteriorly reassessed, showing that the so-called Châtelperronian point does not display the typical features of the Châtelperronian points and “*something similar occurs with the so-called burins, which, in many cases, are facets caused by the fracture of the support. As for scrapers, many of them are simply not retouched or laminar retouches are confused with rock crystal facets. As well as the so-called Dufour leaf, from our point of view, it is nothing more than a fragment of unknapped limestone*.”[4] Therefore, this level provides evidence about the human occupation and the herbivore guild composition in this region during the MIS 3, but the difficulties to obtain a reliable cultural attribution makes more parsimonious to exclude this level from the Bayesian age model for the Châtelperronian techno-complex.

- Levels 8, 9 and 10, Cueva Morín.

Level 10 of Cueva Morin proivded an important set of lithic tools attributed to Châtelperronian. The first dates obtained from this layer provided 26,660±577 BP (SI951) and 35,000±6,777 BP (951a) (uncalibrated); the first date was considered problematic because it is too young, and the second one was problematic due to the large standard deviation. Therefore, for some time, the chronology of this level was assumed to be between that obtained from level 11 (Mousterian) and that obtained from level 8 (Aurignacian) [5]. However, it has been suggested that this level 10 was probably a mixture of Mousterian and Aurignacian industries from the upper and lower layers. Maroto et al. (2012) analysed one sample (GrA-33823) from the sections of level 10 left by previous excavations, which provided an age of 29,380±260 uncal BP. The authors consider that the new date of this level “*cannot be considered satisfactory either, since it is stratigraphically contradictory, inconsistent with the previously available date for this level and too young for a Châtelperronian assemblage. Moreover, the date from level 10 must be viewed with caution as the charcoal has a %C of only 24.0%*”[6]. Likewise, regarding levels 8 and 9, the authors stated: “*Concerning the Early Upper Palaeolithic assemblages, the most disappointing results are those from Morín. Levels 10 to 8 have yielded a series of perfectly reversed dates, inconsistent with the stratigraphical succession and the cultural attribution of these layers. Only the date from level 9 could be considered consistent with the adscription of this layer to the Archaic Aurignacian, but the general picture offered by the Morín dates suggests using this result with caution*.” [6]. Therefore, the stratigraphic issues and the low quality of some radiocarbon dates make more parsimonious to exclude this site from the Bayesian age models.

1.2. End Mousterian

a. Including all the radiocarbon dates.

```
Options()
 {
  Curve("IntCal20","intcal20.14c");
  BCAD=FALSE;
  SD1=TRUE;
  SD2=TRUE;
  ConvergenceData=TRUE;
  kIterations=300;
 };
Plot()
{
  Outlier_Model("General",T(5),U(0,4),"t");
Outlier_Model("SSimple",N(0,2),0,"s");
  Sequence()
  {
    Boundary("End Mousterian");
    Phase("Mousterian")
{
R_Date("El_Castillo_OxA-22205", 49400, 3700)
    {
color="Green";
     Outlier("General", 0.05);
    };
R_Date("El_Castillo_OxA-22204", 48700, 3400)
    {
color="Green";
     Outlier("General", 0.05);
    };
R_Date("Miron_OxA_33516", 48200, 3300)
    {
color="Green";
     Outlier("General", 0.05);
    };
     R_Date("ElCuco_OxA-27115", 46200, 650)
     {
color="Blue";
       Outlier("General", 0.05);
     };
    R_Date("Arrillor_OxA-22658", 45600, 2300)
     {
color="Green";
       Outlier("General", 0.05);
     };
     R_Date("Arrillor_OxA-21986", 44900, 2100)
     {
color="Green";
       Outlier("General", 0.05);
     };
  R_Date("Amalda_OxA-32500", 44500, 2100)
     {
color="Green";
       Outlier("General", 0.05);
     };
```

```
     R_Date("Gatzarria_OxA-25717", 44300, 1900)
     {
color="Green";
       Outlier("General", 0.05);
     };
    R_Combine("comb:La Guelga")
    {
color="Green";
     R_Date("La Guelga_OxA-19244", 43700, 800)
     {
color="Green";
      Outlier("SSimple", 0.05);
     };
     R_Date("La Guelga_OxA-19245", 44300, 1200)
     {
color="Green ";
      Outlier("SSimple", 0.05);
     };
};
      R_Combine("comb:Esquilleu" )
    {
color="Green";
     R_Date("Esquilleu_OxA-19965", 43700, 1400)
     {
color="Green";
      Outlier("SSimple", 0.05);
     };
     R_Date("Esquilleu_OxA-19966", 44100, 1300)
     {
color="Green";
      Outlier("SSimple", 0.05);
     };
    };
     R_Date("Llonin_ConoPosterior_ANU", 43539, 2419)
     {
color="Green";
       Outlier("General", 0.05);
     };
     R_Date("Covalejos_GrA-33811", 43050, 650)
     {
color="Green";
       Outlier("General", 0.05);
     };
     R_Date("Amalda_OxA-34933", 42600, 1600)
     {
color="Green";
       Outlier("General", 0.05);
     };
    R_Date("ElCuco_oXa-27196", 42350, 700)
     {
color="Blue";
       Outlier("General", 0.05);
     };
   };
   Boundary("Final End Mousterian");
  };
 };
};
```

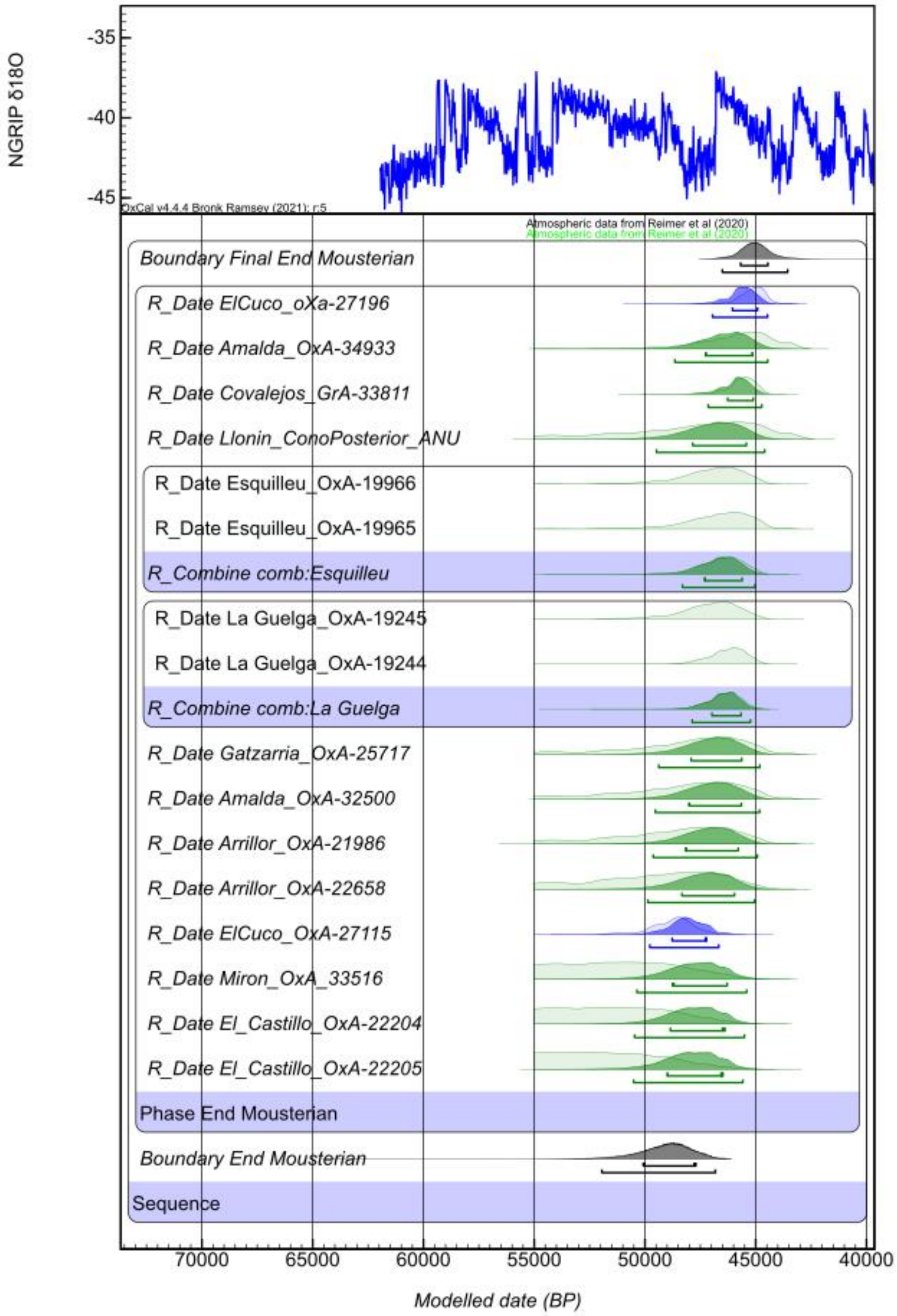

Figure 2. Plot of dated radiocarbon dates from Mousterian assemblages. In blue are indicated the dates obtained from shell remains.

Name	Unmodelled (BP)						Modelled (BP)						Indices				
Amodel 91																	
Aoverall 84.8"																	
	from	to	%	from	to	%	from	to	%	from	to	%	Acomb	A	L	P	C
Boundary Final End Mousterian	Eurosiberian Region						45689	44442	68.26895	46535	43583	95.449974					97.2
R_Date ElCuco_oXa-27196	45537	44506	68.268949	46333	44024	95.449974	46044	44908	68.268949	46953	44477	95.449974		77.6		94.6	99.5
R_Date Amalda_OxA-34933	47160	44027	68.268949	49864	42887	95.449974	47281	45155	68.268949	48662	44467	95.449974		104.8		95.4	99.7
R_Date Covalejos_GrA-33811	46005	44927	68.268949	46837	44556	95.449974	46286	45112	68.268949	47143	44733	95.449974		93.6		95.4	99.6
R_Date Llonin_ConoPosterior_ANU	49598	44241	68.268949	54768	43074	95.449974	47838	45419	68.26895	49474	44593	95.449974		129.3		95.4	99.7
R_Date OxA-19966 Esquilleu VI	47938	45331	68.268949	50169	44489	95.449974											
R_Date OxA-19965 Esquilleu VI	47667	44926	68.268949	50344	44129	95.449974											
R_Combine comb:Esquilleu VI	47313	45407	68.268949	48446	44754	95.449974	47310	45612	68.268949	48287	45032	95.449974		105.3			99.8
R_Date OxA-19245La Guelga D9	47977	45556	68.268949	49922	44696	95.449974											
R_Date OxA-19244La Guelga D9	46885	45309	68.268949	47906	44801	95.449974											
R_Combine comb:La Guelga D9	46915	45550	68.268949	47853	45100	95.449974	46967	45664	68.268949	47859	45239	95.449974		101.8			99.8
R_Date Gatzarria_Cj_OxA-25717	49081	44866	68.268949	54655	44241	95.449973	47906	45631	68.268949	49397	44818	95.449974		127.7		95.5	99.7
R_Date Amalda_VII_OxA-32500	49746	44988	68.268949	54883	44360	95.449974	48006	45655	68.268949	49538	44832	95.449974		128.9		95.5	99.7

R_Date Arrillor_Lmc_OxA-21986	50151	4528 5	68.268 949	54903	4459 7	95.44997 4	48135	45789	68.268 949	49624	44943	95.449 974		126 .9		95 .5	99 .7	
R_Date Arrillor_Smkl-h_OxA-22658	51283	4593 2	68.268 949	...	4520 1	95.44997 3	48327	45963	68.268 949	49827	45058	95.449 974		118 .4		95 .4	99 .7	
R_Date ElCuco_X_OxA-27115	49522	4784 2	68.268 949	50493	4691 7	95.44997 4	48776	47231	68.268 949	49768	46679	95.449 974		93. 3		94 .6	99 .5	
R_Date Miron_130_OxA_33516	54774	4886 2	68.268 949	...	4674 3	95.44997 4	48744	46292	68.268 95	50350	45419	95.449 974		69. 1		95	99 .6	
R_Date El_Castillo_20C_OxA-22204	54959	4989 6	68.268 949	...	4704 8	95.44997 4	48844	46393	68.268 95	50426	45506	95.449 974		60. 6		94 .9	99 .5	
R_Date El_Castillo_20C_OxA-22205	...	5026 6	68.268 949	...	4734 8	95.44997 4	48981	46512	68.268 948	50493	45589	95.449 974		51. 9		94 .8	99 .5	
Phase Mousterian																		
Boundary End Mousterian Eurosiberian					5005 5	47693	68.268 95	51928	46831	95.449 973					95 .1			
Sequence																		
N(0	2)	-2.06	2.06	68.268 949	-4	4	95.449 974											99 .9
Outlier_Model SSimple						...	382	68.268 948	...	1021	95.449 974					96 .8		
U(0	4)	3.99E -17	4	68.268 949	3.99E -17	4	95.449 974	0.06	3.216	68.268 95	5.38E- 17	3.76	95.449 974		10 0			10 0
T(5)	-1.14	1.14	68.268 949	-2.65	2.65	95.44997 4											99 .9	
Outlier_Model General						-279	276	68.268 949	-3097	2288	95.449 974					10 0		
Curve IntCal20																		

b. **Excluding the radiocarbon dates performed on shells (from El Cuco X and XIII)**

```
Options()
 {
  Curve("IntCal20","intcal20.14c");
  BCAD=FALSE;
  SD1=TRUE;
  SD2=TRUE;
  ConvergenceData=TRUE;
  kIterations=300;
 };
Plot()
{
  Outlier_Model("General",T(5),U(0,4),"t");
Outlier_Model("SSimple",N(0,2),0,"s");
  Sequence()
  {
    Boundary("End Mousterian");
    Phase("Mousterian")
{
R_Date("El_Castillo_OxA-22205", 49400, 3700)
    {
color="Green";
      Outlier("General", 0.05);
    };
R_Date("El_Castillo_OxA-22204", 48700, 3400)
    {
color="Green";
Outlier("General", 0.05);
    };
R_Date("Miron_OxA_33516", 48200, 3300)
    {
color="Green";
      Outlier("General", 0.05);
    };
    R_Date("Arrillor_OxA-22658", 45600, 2300)
     {
color="Green ";
       Outlier("General", 0.05);
     };
     R_Date("Arrillor_OxA-21986", 44900, 2100)
     {
color="Green";
       Outlier("General", 0.05);
     };
  R_Date("Amalda_OxA-32500", 44500, 2100)
     {
color="Green";
       Outlier("General", 0.05);
     };
     R_Date("Gatzarria_OxA-25717", 44300, 1900)
     {
color="Green";
       Outlier("General", 0.05);
     };
      R_Combine("comb:La Guelga")
      {
```

```
color="Green";
      R_Date("OxA-19244La Guelga", 43700, 800)
      {
color="Green";
       Outlier("SSimple", 0.05);
      };
      R_Date("OxA-19245La Guelga", 44300, 1200)
      {
color="Green";
       Outlier("SSimple", 0.05);
      };
};
     R_Combine("comb:Esquilleu" )
    {
color="Green";
     R_Date("OxA-19965 Esquilleu", 43700, 1400)
     {
color="Green";
      Outlier("SSimple", 0.05);
     };
     R_Date("OxA-19966 Esquilleu", 44100, 1300)
     {
color="Green";
      Outlier("SSimple", 0.05);
     };
    };
     R_Date("Llonin_ConoPosterior_ANU", 43539, 2419)
     {
color="Green";
       Outlier("General", 0.05);
     };
     R_Date("Covalejos_GrA-33811", 43050, 650)
     {
color="Green";
       Outlier("General", 0.05);
     };
     R_Date("Amalda_OxA-34933", 42600, 1600)
     {
color="Green";
       Outlier("General", 0.05);
     };
   };
   Boundary("Final End Mousterian,");
  };
 };
};
```

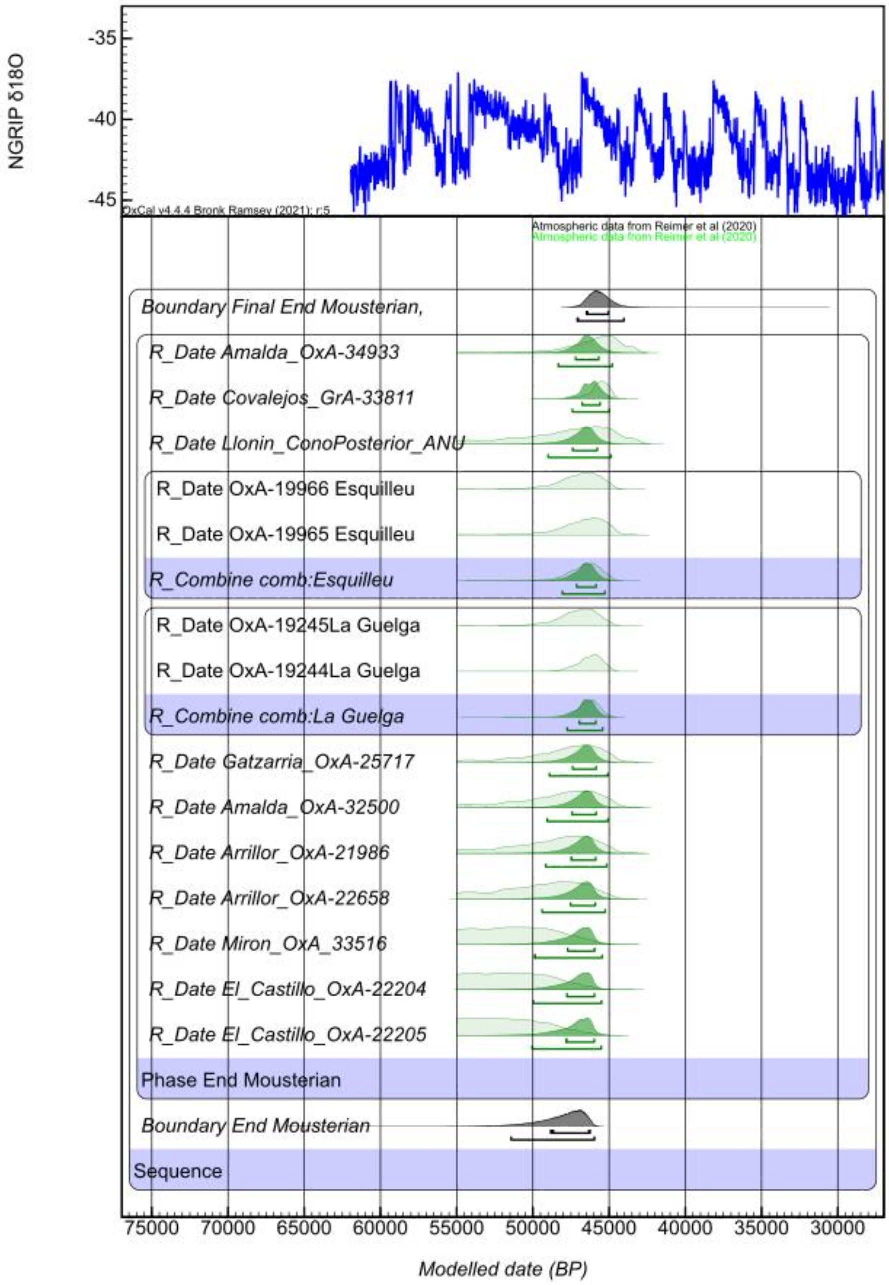

Figure 3. Plot of dated radiocarbon dates from Mousterian assemblages once the radiocarbon dates obtained from shell remains were excluded

Name	Unmodelled (BP)						Modelled (BP)						Indices					
Amodel 76.3																		
Aoverall 72.4"																		
	from	to	%	from	to	%	from	to	%	from	to	%	Acomb	A	L	P	C	
Boundary Final End Mousterian	Eurosiberian Region							46500	45085	68.2689 49	47083	44061	95.4499 74					98.5
R_Date Amalda_VII_OxA-34933	47160	44027	68.2689 49	49864	42887	95.4499 74	47176	45695	68.2689 49	48319	44816	95.4499 74		100.7		95.4	99.9	
R_Date Covalejos_D_GrA-33811	46005	44927	68.2689 49	46837	44556	95.4499 74	46775	45609	68.2689 49	47403	44995	95.4499 74		70		95.1	99.9	
R_Date Llonin_ConoPosterior_ANU	49598	44241	68.2689 49	54768	43074	95.4499 74	47364	45793	68.2689 49	48963	44904	95.4499 74		135.3		95.5	99.9	
R_Date OxA-19966 Esquilleu VI	47938	45331	68.2689 49	50169	44489	95.4499 74												
R_Date OxA-19965 Esquilleu VI	47667	44926	68.2689 49	50344	44129	95.4499 74												
R_Combine P00002 comb:Esquilleu VI	47313	45407	68.2689 49	48446	44754	95.4499 74	47114	45860	68.2689 49	48043	45297	95.4499 74		114.3			99.9	
R_Date OxA-19245La Guelga D9	47977	45556	68.2689 49	49922	44696	95.4499 74												
R_Date OxA-19244La Guelga D9	46885	45309	68.2689 49	47906	44801	95.4499 74												
R_Combine comb:La Guelga D9	46915	45550	68.2689 49	47853	45100	95.4499 74	46949	45881	68.2689 49	47733	45433	95.4499 74		107.8			99.9	
R_Date Gatzarria_Cj_OxA-25717	49081	44866	68.2689 49	54655	44241	95.4499 73	47375	45855	68.2689 49	48880	45087	95.4499 74		136.5		95.6	99.9	
R_Date Amalda_VII_OxA-32500	49746	44988	68.2689 49	54883	44360	95.4499 74	47405	45855	68.2689 49	49016	45084	95.4499 74		136.3		95.5	99.9	
R_Date Arrillor_Lmc_OxA-21986	50151	45285	68.2689 49	54903	44597	95.4499 74	47433	45882	68.2689 49	49112	45162	95.4499 74		132.1		95.5	99.9	
R_Date Arrillor_Smkl-h_OxA-22658	51283	45932	68.2689 49	...	45201	95.4499 73	47498	45910	68.2689 49	49352	45264	95.4499 74		117.2		95.5	99.9	
R_Date Miron_130_OxA_33516	54774	48862	68.2689 49	...	46743	95.4499 74	47673	45955	68.2689 49	49818	45454	95.4499 74		54.9		94.9	99.9	
R_Date El_Castillo_20C_OxA-22204	54959	49896	68.2689 49	...	47048	95.4499 74	47723	45964	68.2689 49	49889	45495	95.4499 74		45.5		94.7	99.9	
R_Date El_Castillo_20C_OxA-22205	...	50266	68.2689 49	...	47348	95.4499 74	47757	45972	68.2689 49	49965	45532	95.4499 74		36.9		94.5	99.9	
Phase Mousterian																		

Boundary End Mousterian							48733	46263	68.2689 49	51320	45953	95.4499 74			97. 1	
Sequence																
N(0	2)	-2.06	2.06	68.2689 49	-4	4	95.4499 74									99. 9
Outlier_Model SSimple							...	298	68.2689 5	...	987	95.4499 74			98. 6	
U(0	4)	3.99E-17	4	68.2689 49	3.99E- 17	4	95.4499 74	5.38E- 17	2.984	68.2689 49	5.38E- 17	3.776	95.4499 74	10 0		10 0
T(5)	-1.14	1.14	68.2689 49	-2.65	2.65	95.4499 74									99. 9	
Outlier_Model General							-268	265	68.2689 49	-3604	2095	95.4499 74			10 0	
Curve IntCal20																

c. Excluding the oldest (OxA-22205) and youngest (oXa-27196) dates.

```
Options()
 {
  Curve("IntCal20","intcal20.14c");
  BCAD=FALSE;
  SD1=TRUE;
  SD2=TRUE;
  ConvergenceData=TRUE;
  kIterations=300;
 };
Plot()
{
  Outlier_Model("General",T(5),U(0,4),"t");
Outlier_Model("SSimple",N(0,2),0,"s");
  Sequence()
  {
    Boundary("End Mousterian");
    Phase("Mousterian")
{
R_Date("El_Castillo_OxA-22204", 48700, 3400)
    {
color="Green";
     Outlier("General", 0.05);
    };
R_Date("Miron_OxA_33516", 48200, 3300)
    {
color="Green";
     Outlier("General", 0.05);
    };
    R_Date("Arrillor_OxA-22658", 45600, 2300)
     {
color="Green";
       Outlier("General", 0.05);
     };
     R_Date("Arrillor_OxA-21986", 44900, 2100)
     {
color="Green";
       Outlier("General", 0.05);
     };
  R_Date("Amalda_OxA-32500", 44500, 2100)
     {
color="Green";
       Outlier("General", 0.05);
     };
     R_Date("Gatzarria_OxA-25717", 44300, 1900)
     {
color="Green";
       Outlier("General", 0.05);
     };
     R_Combine("comb:La Guelga")
     {
color="Green";
      R_Date("OxA-19244La Guelga", 43700, 800)
      {
color="Green";
```

```
 Outlier("SSimple", 0.05);
 };
 R_Date("OxA-19245La Guelga", 44300, 1200)
 {
color="Green";
 Outlier("SSimple", 0.05);
 };
};
 R_Combine("comb:Esquilleu" )
 {
color="Green";
 R_Date("OxA-19965 Esquilleu", 43700, 1400)
 {
color="Green";
 Outlier("SSimple", 0.05);
 };
 R_Date("OxA-19966 Esquilleu", 44100, 1300)
 {
color="Green";
 Outlier("SSimple", 0.05);
 };
 };
 R_Date("Llonin_ConoPosterior_ANU", 43539, 2419)
 {
color="Green";
 Outlier("General", 0.05);
 };
 R_Date("Covalejos_GrA-33811", 43050, 650)
 {
color="Green";
 Outlier("General", 0.05);
 };
 R_Date("Amalda_OxA-34933", 42600, 1600)
 {
color="Green";
 Outlier("General", 0.05);
 };
 };
 Boundary("Final End Mousterian");
 };
 };
};
```

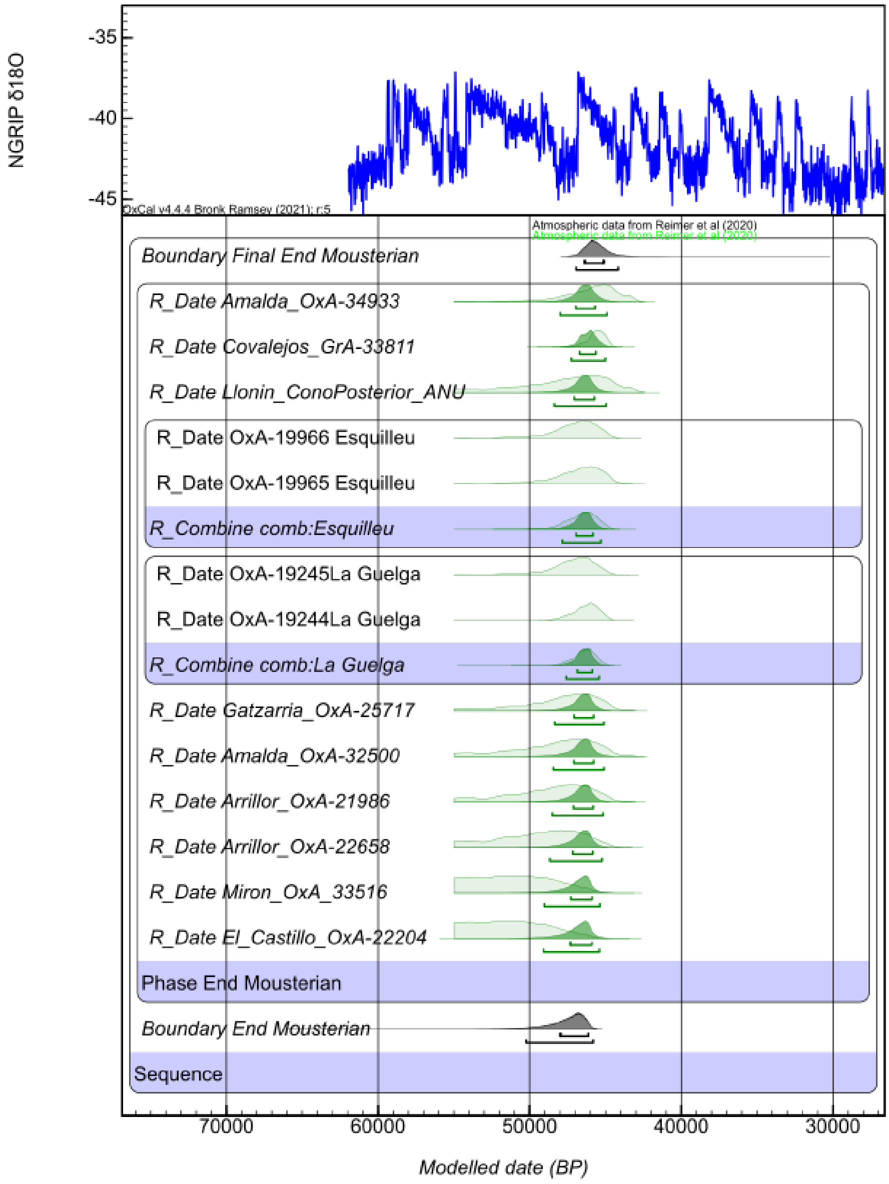

Figure 4. Plot of dated radiocarbon dates from Mousterian assemblages once the oldest and youngest radiocarbon dates were removed

Name	Unmodelled (BP)						Modelled (BP)						Indices					
Amodel 88.8																		
Aoverall 90.9"																		
	from	to	%	from	to	%	from	to	%	from	to	%	Acomb	A	L	P	C	
Boundary Final End Mousterian	Eurosiberian Region							46437	45151	68.26895	46926	44183	95.449974					98.1
R_Date Amalda_VII_OxA-34933	47160	44027	68.268949	49864	42887	95.449974	46941	45680	68.268949	47966	44909	95.449974		106.4		95.5	99.9	
R_Date Covalejos_D_GrA-33811	46005	44927	68.268949	46837	44556	95.449974	46697	45635	68.268949	47263	45027	95.449974		73.5		95.2	99.9	
R_Date Llonin_ConoPosterior_ANU	49598	44241	68.268949	54768	43074	95.449974	47045	45725	68.268949	48355	44974	95.449974		139.4		95.5	99.9	
R_Date OxA-19966 Esquilleu VI	47938	45331	68.268949	50169	44489	95.449974												
R_Date OxA-19965 Esquilleu VI	47667	44926	68.268949	50344	44129	95.449974												
R_Combine comb:Esquilleu VI	47313	45407	68.268949	48446	44754	95.449974	46925	45815	68.268949	47827	45305	95.449974		119.4			99.9	
R_Date OxA-19245La Guelga D9	47977	45556	68.268949	49922	44696	95.449974												
R_Date OxA-19244La Guelga D9	46885	45309	68.268949	47906	44801	95.449974												
R_Combine comb:La Guelga D9	46915	45550	68.268949	47853	45100	95.449974	46833	45859	68.268949	47566	45415	95.449974		113.1			99.9	
R_Date Gatzarria_Cj_OxA-25717	49081	44866	68.268949	54655	44241	95.449973	47058	45774	68.268949	48323	45123	95.449974		139		95.6	99.9	
R_Date Amalda_VII_OxA-32500	49746	44988	68.268949	54883	44360	95.449974	47081	45778	68.268949	48411	45123	95.449974		137.8		95.5	99.9	
R_Date Arrillor_Lmc_OxA-21986	50151	45285	68.268949	54903	44597	95.449974	47108	45801	68.268949	48483	45180	95.449974		131.5		95.5	99.9	
R_Date Arrillor_Smkl-h_OxA-22658	51283	45932	68.268949	...	45201	95.449973	47158	45831	68.268949	48634	45243	95.449974		113		95.4	99.9	
R_Date Miron_130_OxA_33516	54774	48862	68.268949	...	46743	95.449974	47264	45877	68.268949	48999	45375	95.449974		46.4		94.8	99.9	
R_Date El_Castillo_20C_OxA-22204	54959	49896	68.268949	...	47048	95.449974	47291	45890	68.268949	49048	45409	95.449974		37		94.6	99.8	
Phase Mousterian																		
Boundary End Mousterian							48007	46130	68.268948	50183	45790	95.449974					97.4	

Sequence

N(0	2)	-2.06	2.06	68.26894 9	-4	4	95.44997 4									10 0
Outlier_Model SSimple							...	346	68.26895	...	1030	95.44997 3			98. 3	
U(0	4)	3.99E-17	4	68.26894 9	3.99E- 17	4	95.44997 4	5.38E- 17	2.796	68.26894 9	5.38E- 17	3.776	95.44997 4	10 0		10 0
T(5)	-1.14	1.14	68.26894 9	-2.65	2.65	95.44997 4									99. 9	
Outlier_Model General							-232	235	68.26894 9	-3134	1947	95.44997 4			10 0	

Curve IntCal20

1.3. Châtelperronian

2. Including all the radiocarbon dates (in blue, problematic dates, see the "Validation and sensitivity tests" section for details)

```
Options()
 {
  Curve("IntCal20","intcal20.14c");
  BCAD=FALSE;
  SD1=TRUE;
  SD2=TRUE;
  ConvergenceData=TRUE;
  kIterations=300;
 };
Plot()
{
  Outlier_Model("General",T(5),U(0,4),"t");
  Sequence()
  {
    Boundary("Start Châtelperronian Eurosiberian ");
    Phase("Châtelperronian")
{
        Age("TL_Arranblatxa", N(43500, 2900))
     {
color="Green";
       Outlier("General", 0.05);
     };
     R_Date("La Güelga_OxA-27958", 40300, 1200)
     {
color="Green";
       Outlier("General", 0.05);
     };
     R_Date("LabekoKoba_OxA-22563", 38100, 900)
     {
color="Green";
       Outlier("General", 0.05);
     };
     R_Date("LabekoKoba_OxA-22560", 38000, 900)
     {
color="Green";
       Outlier("General", 0.05);
     };
R_Date("LabekoKoba_OxA-22562", 37800, 900)
     {
color="Green";
       Outlier("General", 0.05);
     };
     R_Date("LabekoKoba_OxA-22561", 37400, 800)
     {
color="Green";
       Outlier("General", 0.05);
     };
     R_Date("LaGüelga_COL2014", 37429, 302)
     {
color="Green";
       Outlier("General", 0.05);
     };
        R_Date("Ekain_OxA-34930", 34350, 550)
```

```
    {
color="Green";
     Outlier("General", 0.05);
    };
    };
   Boundary("End Châtelperronian");
  };
 };
};
```

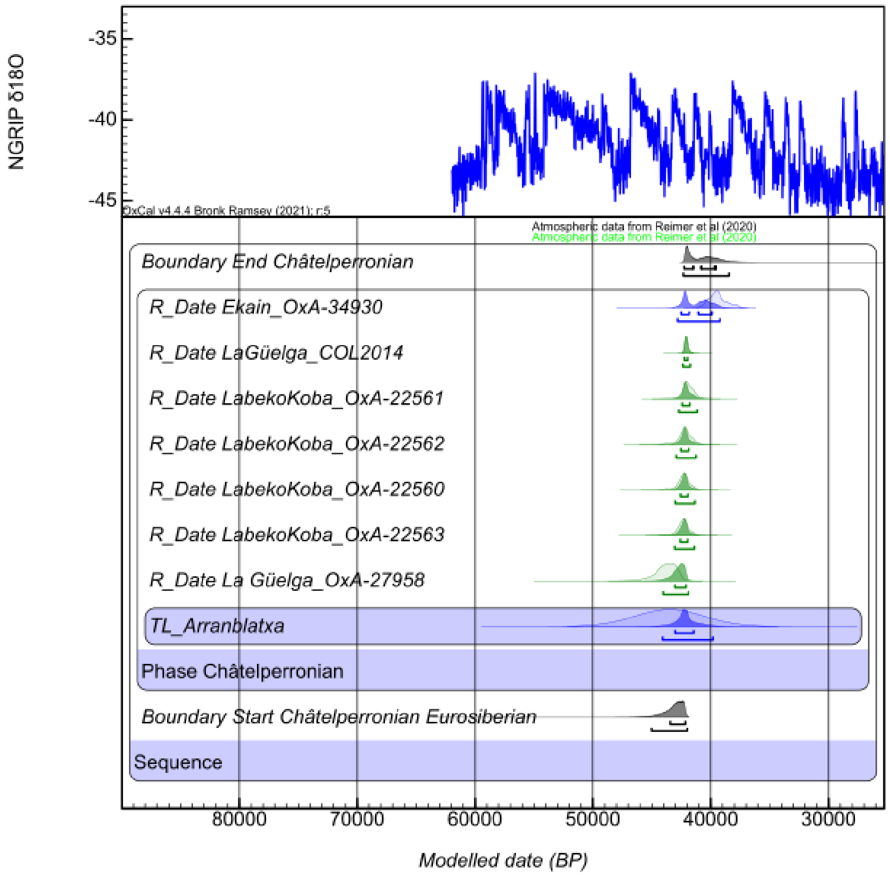

Figure 5. Plot of dated radiocarbon dates from Châtelperronian assemblages in the Eurosiberian region.

Name	Unmodelled (BP)						Modelled (BP)						Indices					
Amodel 79.8																		
Aoverall 88.1"																		
	from	to	%	from	to	%	from	to	%	from	to	%	Acomb	A	L	P	C	
Boundary End ChÃ¢telperronian							42262	39568	68.268949	42333	38444	95.449974					95	
R_Date Ekain_OxA-34930	40371	38960	68.268949	40710	37733	95.449974	42507	39904	68.268949	42804	39217	95.449974		38.8		53.4	97.7	
R_Date LaGÃ¼elga_COL2014	42215	41920	68.268949	42344	41671	95.449974	42241	41957	68.268949	42369	41720	95.449974		103.6		97	99.9	
R_Date LabekoKoba_OxA-22561	42363	41497	68.268949	42777	40986	95.449974	42422	41766	68.268949	42699	41150	95.449974		112		97	99.7	
R_Date LabekoKoba_OxA-22562	42637	41678	68.268949	43249	41012	95.449974	42527	41874	68.268949	42917	41250	95.449974		119.8		97	99.8	
R_Date LabekoKoba_OxA-22560	42750	41824	68.268949	43785	41136	95.449974	42563	41932	68.268949	42999	41343	95.449974		120.4		97	99.8	
R_Date LabekoKoba_OxA-22563	42792	41880	68.268949	43830	41224	95.449974	42581	41957	68.268949	43041	41385	95.449974		120.1		97	99.8	
R_Date La GÃ¼elga_OxA-27958	44396	42776	68.268949	45660	42312	95.449974	43028	42116	68.268949	44029	41895	95.449974		71.4		91	99.2	
N(43500	2900)	40513	46487	68.268949	37700	49300	95.449974	41441	43007	68.268949	39817	44080	95.449974		122.9		95.8	99.6
#¿NOMBRE?							1950	1951	68.268949	1950	1951	95.449974					100	
TL_Arranblatxa	46487	40513	68.268949	49300	37700	95.449974	43007	41441	68.268949	44080	39817	95.449974					99.6	
Phase ChÃ¢telperronian																		
Boundary Start ChÃ¢telperronian Eurosiberian							43452	42163	68.268949	45028	42009	95.449974					97.1	
Sequence																		
U(0	4)	3.99E-17	4	68.268949	3.99E-17	4	95.449974	2.16	4	68.268949	5.38E-17	4	95.449973		100			99.4
T(5)	-1.14	1.14	68.268949	-2.65	2.65	95.449974											99.5	
Outlier_Model General							-205	3409	68.268949	-1861	4303	95.449974					98.7	
Curve IntCal20																		

3. Excluding the oldest (Aranbaltxa) and youngest (OxA-34930) dates.

```
Options()
 {
  Curve("IntCal20","intcal20.14c");
  BCAD=FALSE;
  SD1=TRUE;
  SD2=TRUE;
  ConvergenceData=TRUE;
  kIterations=300;
 };
Plot()
{
  Outlier_Model("General",T(5),U(0,4),"t");
  Sequence()
  {
   Boundary("Start Châtelperronian Eurosiberian ");
   Phase("Châtelperronian")
{
    R_Date("La Güelga_OxA-27958", 40300, 1200)
    {
color="Green";
     Outlier("General", 0.05);
    };
    R_Date("LabekoKoba_OxA-22563", 38100, 900)
    {
color="Green";
     Outlier("General", 0.05);
    };
    R_Date("LabekoKoba_OxA-22560", 38000, 900)
    {
color="Green";
     Outlier("General", 0.05);
    };
R_Date("LabekoKoba_OxA-22562", 37800, 900)
    {
color="Green";
     Outlier("General", 0.05);
    };
    R_Date("LabekoKoba_OxA-22561", 37400, 800)
    {
color="Green";
     Outlier("General", 0.05);
    };
    R_Date("LaGüelga_COL2014", 37429, 302)
    {
color="Green";
     Outlier("General", 0.05);
    };
    };
   Boundary("End Châtelperronian");
  };
 };
 };
```

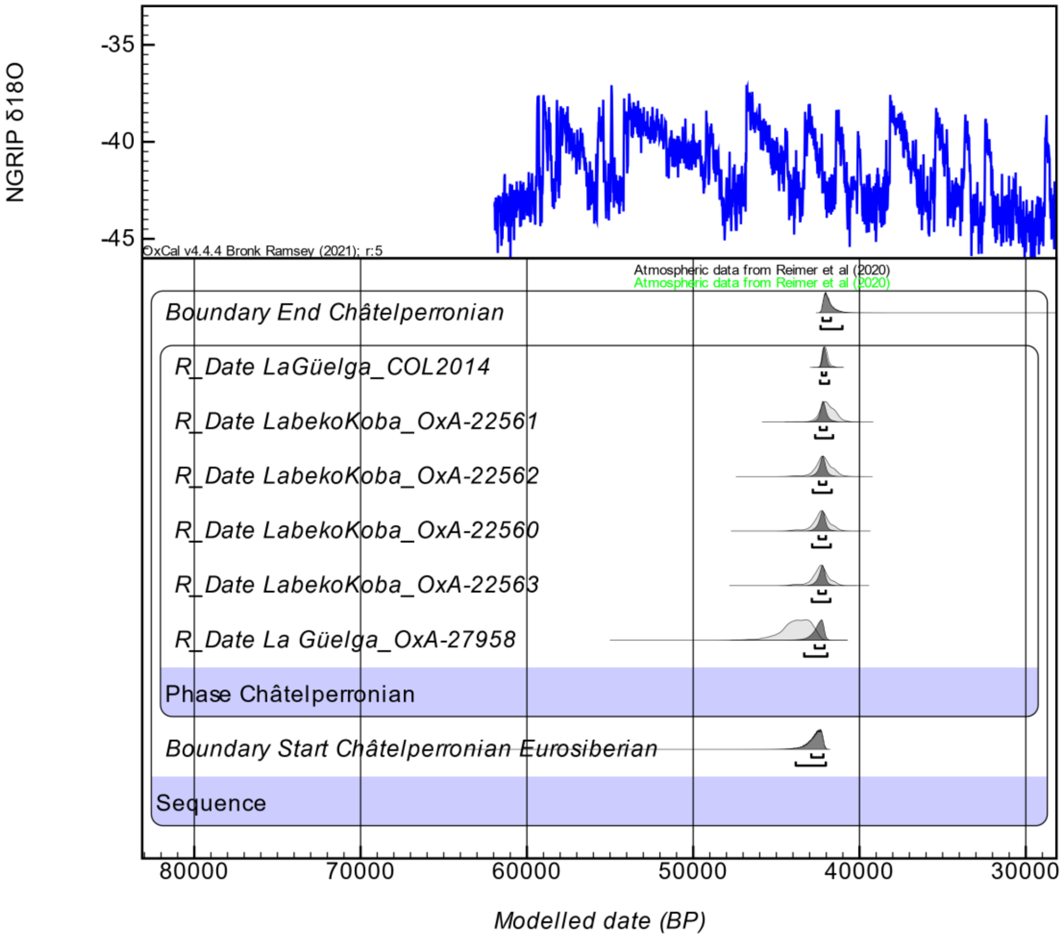

Figure 6. Plot of dated radiocarbon dates from Châtelperronian assemblages in the Eurosiberian region once the oldest and youngest dates were removed.

Name	Unmodelled (BP)						Modelled (BP)						Indices				
Amodel 104.9																	
Aoverall 114.6"																	
	from	to	%	from	to	%	from	to	%	from	to	%	Acomb	A	L	P	C
Boundary End ChÃ¢telperronian							42221	41725	68.268949	42340	41029	95.449974					98.3
R_Date LaGÃ¼elga_COL2014	42215	41920	68.268949	42344	41671	95.449974	42261	42011	68.268949	42370	41835	95.449974		103.6		96.1	100
R_Date LabekoKoba_OxA-22561	42363	41497	68.268949	42777	40986	95.449974	42381	41977	68.268949	42664	41595	95.449974		119.1		96.1	99.9
R_Date LabekoKoba_OxA-22562	42637	41678	68.268949	43249	41012	95.449974	42433	42006	68.268949	42805	41675	95.449974		133.8		96.2	99.9
R_Date LabekoKoba_OxA-22560	42750	41824	68.268949	43785	41136	95.449974	42453	42023	68.268949	42847	41735	95.449974		134.5		96.2	99.9
R_Date LabekoKoba_OxA-22563	42792	41880	68.268949	43830	41224	95.449974	42465	42030	68.268949	42865	41758	95.449974		133.4		96.2	99.9
R_Date La GÃ¼elga_OxA-27958	44396	42776	68.268949	45660	42312	95.449974	42684	42107	68.268949	43325	41935	95.449974		48.4		92.1	99.9
Phase ChÃ¢telperronian																	
Boundary Start ChÃ¢telperronian Eurosiberian							42896	42174	68.268949	43831	42023	95.449974					97.5
Sequence																	
U(0	4)	3.99E-17	4	68.268949	3.99E-17	4	95.449974	5.38E-17	2.904	68.268949	5.38E-17	3.776	95.449974	100			100
T(5)	-1.14	1.14	68.268949	-2.65	2.65	95.449974											99.9
Outlier_Model General							-191	184	68.268949	-1790	698	95.449974					100
Curve IntCal20																	

•

1.4. Aurignacian

a. Including all the radiocarbon dates

```
Options()
 {
  Curve("IntCal20","intcal20.14c");
  BCAD=FALSE;
  SD1=TRUE;
  SD2=TRUE;
  ConvergenceData=TRUE;
  kIterations=300;
 };
Plot()
{
  Outlier_Model("General",T(5),U(0,4),"t");
Outlier_Model("SSimple",N(0,2),0,"s");
  Sequence()
  {
    Boundary("Start Aurignacian");
    Phase("Aurignacian")
{
  R_Date("Isturitz_AA69184",40200,3600)
{
color="Green";
Outlier(0.05);
};
R_Date("ElCastillo_OxA-22200", 38600, 1000)
     {
color="Green";
       Outlier("General", 0.05);
     };
R_Date("Isturitz_OxA-23435",37500,900)
{
color="Green";
Outlier(0.05);
};
R_Date("Isturitz_AA69183",37580,780)
{
color="Green";
Outlier(0.05);
};
R_Date("Isturitz_AA69180",37300,1800)
{
color="Green";
Outlier(0.05);
};
R_Date("Isturitz_OxA-23436",37400,900)
{
color="Green";
Outlier(0.05);
};
R_Date("Isturitz_OxA-23434",37000,800)
{
color="Green";
Outlier(0.05);
};
```

```
R_Date(“Isturitz_OxA-23432”,37000,800)
{
color="Green";
Outlier(0.05);
};
R_Date(“Isturitz_AA69179”,37000,1600)
{
color="Green";
Outlier(0.05);
};
R_Date(“Isturitz_AA69185”,36990,720)
{
color="Green";
Outlier(0.05);
};
R_Date(“Isturitz_AA69181”,36800,860)
{
color="Green";
Outlier(0.05);
};
  R_Date("LabekoKoba_OxA-21766",36850,800)
  {
color="Green";
   Outlier(0.05);
  };
  R_Date("LabekoKoba_OxA-X-2314-43",36500,750)
  {
color="Green";
   Outlier(0.05);
  };
  R_Date("Gatzarria_OxA-22554",36300,700)
  {
color="Green";
   Outlier(0.05);
  };
R_Combine("comb:Labeko")
    {
color="Green";
     R_Date("OxA-21793 Labeko", 35400, 650)
     {
color="Green";
      Outlier("SSimple", 0.05);
     };
     R_Date("OxA-21840 Labeko", 35250, 650)
     {
color="Green";
      Outlier("SSimple", 0.05);
     };
    };
  R_Date("LabekoKoba_OxA-21778", 35100, 600)
  {
color="Green";
   Outlier("General", 0.05);
  };

R_Date("Cobrante_OxA-32505", 35150, 650)
   {
```

```
color="Green";
   Outlier("General", 0.05);
  };
   R_Date("ElCuco_OxA-32502", 35050, 650)
   {
color="Green";
    Outlier("General", 0.05);
   };
R_Date("Covalejos_OxA-32511", 35250, 700)
   {
color="Green";
    Outlier("General", 0.05);
   };
R_Date("Covalejos_OxA-32512", 34850, 600)
   {
color="Green";
    Outlier("General", 0.05);
   };
   R_Date("Covalejos_OxA-32513", 35150, 650)
   {
color="Green";
    Outlier("General", 0.05);
   };
   R_Date("Covalejos_OxA-32549", 34350, 600)
   {
color="Green";
    Outlier("General", 0.05);
   };
  R_Date("LabekoKoba_OxA-21767", 34750, 600)
  {
color="Green";
  Outlier("General", 0.05);
  };
  R_Date("LaGüelga_OxA-30809",34750,600)
  {
color="Green";
  Outlier(0.05);
  };
  R_Date("LabekoKoba_OxA-21779", 34650, 600)
  {
color="Green";
  Outlier("General", 0.05);
  };
  R_Date("Gatzarria_OxA-22555",34400,550)
  {
color="Green";
  Outlier(0.05);
  };
  R_Date("Gatzarria_OxA-22556",34250,550)
  {
color="Green";
  Outlier(0.05);
  };
  R_Date("LaGüelga_COL2579.2.1",34500,763)
  {
color="Green";
  Outlier(0.05);
  };
  R_Date("LaGüelga_COL2578.2.1",34355,751)
```

```
   {
color="Green";
    Outlier(0.05);
   };
   R_Date("LaGüelga_COL2578.1.1",34164,273)
   {
color="Green";
    Outlier(0.05);
   };
   R_Date("LaGüelga_COL2579.1.1",34121,266)
   {
color="Green";
    Outlier(0.05);
   };
   R_Date("LaGüelga_OxA-30810",33900,550)
   {
color="Green";
    Outlier(0.05);
   };
   R_Date("Gatzarria_OxA-22553",33800,550)
   {
color="Green";
    Outlier(0.05);
   };
   R_Date("LaGüelga_Beta-377233",33610,220)
   {
color="Green";
    Outlier(0.05);
   };
    R_Combine("comb:Ekain")
    {
color="Green";
     R_Date("OxA-32423 Ekain", 31140, 400)
     {
color="Green";
      Outlier("SSimple", 0.05);
     };
     R_Date("OxA-32424 Ekain", 31100, 400)
     {
color="Green";
      Outlier("SSimple", 0.05);
     };
     };
    R_Date ("Aitz_OxA_34932", 31130, 390)
    {
color="Green";
     Outlier("General", 0.05);
    };
R_Date ("Aitz_OxA-32416", 30990, 390)
    {
color="Green";
     Outlier("General", 0.05);
    };
  };
   Boundary("End Aurignacian");
  };
  };
 };
```

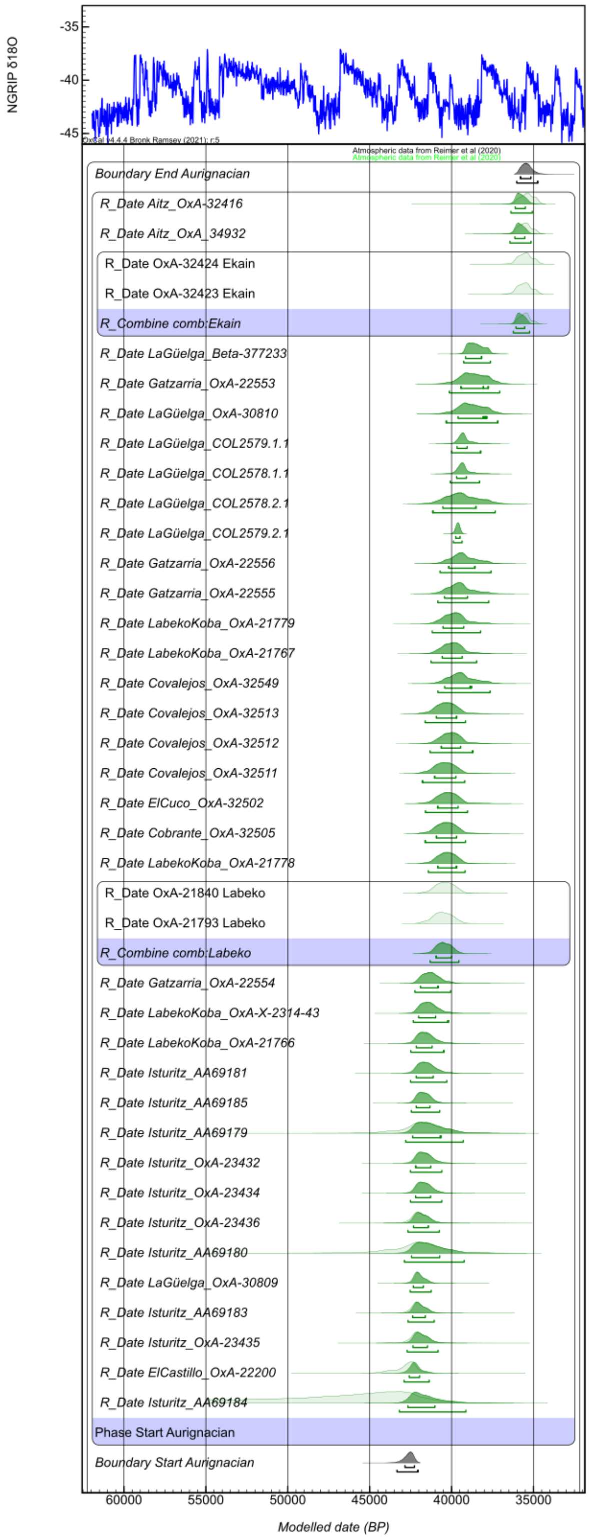

Figure 7. Plot of dated radiocarbon dates from Aurignacian assemblages in the Eurosiberian region

Name	Unmodelled (BP)						Modelled (BP)						Indices					
Amodel 106.8																		
Aoverall 106"																		
	from	to	%	from	to	%	from	to	%	from	to	%	Acomb	A	L	P	C	
Boundary Start Aurignacian	Eurosiberian							35786	35140	68.268949	36012	34735	95.449974					95.9
R_Date Aitz_III_Vb_OxA-32416	35777	34870	68.268949	36156	34616	95.449974	36105	35477	68.268949	36376	35075	95.449974		88.8		94.8	98.7	
R_Date Aitz_III_Vb_OxA_34932	36031	35195	68.268949	36245	34690	95.449974	36128	35523	68.268949	36434	35145	95.449974		100.6		95.2	98.8	
R_Date OxA-32424 Ekain IXb	36030	35153	68.268949	36237	34662	95.449974												
R_Date OxA-32423 Ekain IXb	36046	35197	68.268949	36266	34682	95.449974												
R_Combine Ekain IXb: comb	35898	35244	68.268949	36128	34826	95.449974	36067	35522	68.268949	36228	35237	95.449974		95.2			99	
R_Date LaGÃ¼elga_Dinterior5_Beta-377233	39140	38180	68.268949	39240	37648	95.449974	39146	38171	68.268949	39257	37626	95.449974		101		96.1	99.3	
R_Date Gatzarria_Cjn2_OxA-22553	39415	37815	68.268949	40051	37090	95.449974	39422	37779	68.268949	40145	37048	95.449974		100.6		95.7	99.1	
R_Date LaGÃ¼elga_Dinterior5_OxA-30810	39602	37863	68.268949	40237	37216	95.449974	39613	37805	68.26895	40332	37187	95.449974		100.4		95.5	99.2	
R_Date LaGÃ¼elga_Dinterior5_COL2579.1.1	39643	39066	68.268949	39928	38392	95.449974	39664	39044	68.268949	39995	38216	95.449974		98.1		93.2	99.3	
R_Date LaGÃ¼elga_Dinterior5_COL2578.1.1	39667	39108	68.268949	39981	38451	95.449974	39690	39089	68.268949	40072	38271	95.449974		97.9		93.2	99.3	
R_Date LaGÃ¼elga_Dinterior5_COL2578.2.1	40506	38546	68.268949	41099	37440	95.449974	40524	38483	68.268949	41136	37323	95.449973		100.3		95.4	99.1	
R_Date LaGÃ¼elga_Dinterior5_COL2579.2.1	39743	39484	68.268949	39880	39366	95.449974	39746	39481	68.268949	39888	39361	95.449974		99.9		95	99.7	
R_Date Gatzarria_Cbf_OxA-22556	40121	38594	68.268949	40615	37640	95.449974	40197	38574	68.26895	40698	37571	95.449974		99.8		94.8	99	
R_Date Gatzarria_Cbf_OxA-22555	40395	39055	68.268949	40789	37817	95.449974	40428	39024	68.268949	40850	37722	95.449974		99.5		94.7	99.1	
R_Date LabekoKoba_VI_OxA-21779	40517	39270	68.268949	41168	38269	95.449974	40530	39265	68.268949	41178	38217	95.449973		100.4		95.3	99.1	

R_Date LabekoKoba_VI_OxA-21767	40560	39358	68.268949	41226	38507	95.449974	40578	39353	68.26895	41245	38458	95.449974	100.3	95.3	99.1
R_Date Covalejos_B_ OxA-32549	40425	38853	68.268949	40805	37661	95.449974	40420	38834	68.268949	40820	37643	95.449974	100.4	95.3	99
R_Date Covalejos_B_ OxA-32513	40922	39704	68.268949	41569	39161	95.449974	40930	39698	68.268949	41632	39155	95.449974	100.4	95.4	99.2
R_Date Covalejos_C_OxA-32512	40622	39446	68.268949	41311	38770	95.449974	40641	39444	68.268949	41323	38752	95.449974	100.4	95.4	99
R_Date Covalejos_C_OxA-32511	41036	39749	68.268949	41750	39209	95.449974	41045	39729	68.268949	41779	39192	95.449974	100.4	95.4	99.1
R_Date ElCuco_III_OxA-32502	40841	39608	68.268949	41569	39042	95.449974	40858	39601	68.268949	41610	39028	95.449974	100.4	95.4	99.1
R_Date Cobrante_VI_ OxA-32505	40922	39704	68.268949	41569	39161	95.449974	40943	39697	68.268949	41591	39146	95.449974	100.4	95.4	99
R_Date LabekoKoba_VI_OxA-21778	40836	39693	68.268949	41380	39195	95.449974	40846	39690	68.268949	41424	39172	95.449974	100.4	95.4	99.2
R_Date OxA-21840 Labeko_VII	40998	39790	68.268949	41608	39251	95.449974									
R_Date OxA-21793 Labeko_VII	41114	39914	68.268949	41713	39391	95.449974									
R_Combine Labeko_VII: comb	40929	40008	68.268949	41282	39576	95.449974	40944	39994	68.26895	41299	39553	95.449974	99.2		99.3
R_Date Gatzarria_Cjn2_OxA-22554	41891	40840	68.268949	42218	40133	95.449974	41899	40815	68.268949	42242	40048	95.449974	100.7	95.4	99.1
R_Date LabekoKoba_VII_OxA-X-2314-43	42004	40980	68.268949	42347	40270	95.449974	42017	40978	68.268949	42339	40145	95.449974	100.9	95.4	99.2
R_Date LabekoKoba_VII_OxA-21766	42150	41192	68.268949	42514	40531	95.449974	42150	41186	68.268949	42486	40446	95.449974	102.1	95.4	99.2
R_Date Isturitz_C4c4_AA69181	42165	41115	68.268949	42581	40359	95.449974	42148	41110	68.268949	42509	40280	95.449974	102.5	95.4	99.2
R_Date Isturitz_C4c4_AA69185	42164	41327	68.268949	42480	40793	95.449974	42159	41309	68.268948	42454	40713	95.449974	101.8	95.3	99.2
R_Date Isturitz_C4c4_AA69179	42753	40457	68.268949	44635	39212	95.449974	42370	40668	68.268949	42805	39296	95.449974	115.5	95.8	99
R_Date Isturitz_C4d1j'_OxA-23432	42202	41282	68.268949	42568	40653	95.449974	42189	41275	68.268949	42514	40590	95.449974	102.6	95.3	99.2
R_Date Isturitz_C4d1j'_OxA-23434	42202	41282	68.268949	42568	40653	95.449974	42187	41282	68.268949	42511	40607	95.449974	102.6	95.4	99
R_Date Isturitz_C4d1j'_OxA-23436	42400	41415	68.268949	42925	40775	95.449974	42323	41414	68.268949	42680	40745	95.449974	106.1	95.2	99.1
R_Date Isturitz_C4c4_AA69180	43188	40392	68.268949	45941	39116	95.449974	42442	40705	68.268949	42883	39225	95.449974	120.2	95.9	98.9
R_Date LaGÃ¼elga_Dinterior5_OxA-30809	42365	41745	68.268949	42567	41294	95.449974	42334	41722	68.268949	42524	41245	95.449974	102.2	94.9	99.3

R_Date Isturitz_C4c4_AA69183		42455	41630	68.268949	42831	41125	95.449974	42377	41618	68.268949	42659	41056	95.449974		104.8		95	99.2	
R_Date Isturitz_C4d1j'_OxA-23435		42445	41469	68.268949	42994	40843	95.449974	42357	41469	68.26895	42704	40814	95.449974		106.8		95.1	99	
R_Date ElCastillo_16_OxA-22200		43136	42020	68.268949	44283	41583	95.449974	42592	41955	68.268949	42910	41366	95.449974		107.8		95.1	98.8	
R_Date Isturitz_C4c4_AA69184		48935	41531	68.268949	...	41033	95.449974	42675	41003	68.268949	43186	39158	95.449974		81.1		95	98.6	
Phase Start Aurignacian																			
Boundary End Aurignacian								42843	42265	68.268949	43330	42059	95.449974					95.4	
Sequence																			
N(0	2)		-2.06	2.06	68.268949	-4	4	95.449974											100
Outlier_Model SSimple								-516	782	68.26895	...	...	95.449974					99.5	
U(0	4)		3.99E-17	4	68.268949	3.99E-17	4	95.449974	5.38E-17	3.112	68.268949	5.38E-17	3.744	95.449974		100			99.9
T(5)		-1.14	1.14	68.268949	-2.65	2.65	95.449974											99.6	
Outlier_Model General								-218	233	68.268949	-2034	1970	95.449974					100	
Curve IntCal20																			

b. Excluding the oldest (AA69184) and youngest (OxA-32416) dates.

```
Options()
 {
  Curve("IntCal20","intcal20.14c");
  BCAD=FALSE;
  SD1=TRUE;
  SD2=TRUE;
  ConvergenceData=TRUE;
  kIterations=300;
 };
Plot()
{
  Outlier_Model("General",T(5),U(0,4),"t");
Outlier_Model("SSimple",N(0,2),0,"s");
  Sequence()
  {
    Boundary("Start Aurignacian");
    Phase("Aurignacian")
{
R_Date("ElCastillo_OxA-22200", 38600, 1000)
     {
color="Green";
       Outlier("General", 0.05);
     };
R_Date("Isturitz_OxA-23435",37500,900)
{
color="Green";
Outlier(0.05);
};
R_Date("Isturitz_AA69183",37580,780)
{
color="Green";
Outlier(0.05);
};
    R_Date("LaGüelga_OxA-30809",37500,600)
    {
color="Green";
     Outlier(0.05);
    };
R_Date("Isturitz_AA69180",37300,1800)
{
color="Green";
Outlier(0.05);
};
R_Date("Isturitz_OxA-23436",37400,900)
{
color="Green";
Outlier(0.05);
};
R_Date("Isturitz_OxA-23434",37000,800)
{
color="Green";
Outlier(0.05);
};
```

```
R_Date("Isturitz_OxA-23432",37000,800)
{
color="Green";
Outlier(0.05);
};
R_Date("Isturitz_AA69179",37000,1600)
{
color="Green";
Outlier(0.05);
};
R_Date("Isturitz_AA69185",36990,720)
{
color="Green";
Outlier(0.05);
};
R_Date("Isturitz_AA69181",36800,860)
{
color="Green";
Outlier(0.05);
};
   R_Date("LabekoKoba_OxA-21766",36850,800)
   {
color="Green";
    Outlier(0.05);
   };
   R_Date("LabekoKoba_OxA-X-2314-43",36500,750)
   {
color="Green";
    Outlier(0.05);
   };
   R_Date("Gatzarria_OxA-22554",36300,700)
   {
color="Green";
    Outlier(0.05);
   };
R_Combine("comb:Labeko")
     {
color="Green";
      R_Date("OxA-21793 Labeko", 35400, 650)
      {
color="Green";
       Outlier("SSimple", 0.05);
      };
      R_Date("OxA-21840 Labeko", 35250, 650)
      {
color="Green";
       Outlier("SSimple", 0.05);
      };
     };
   R_Date("LabekoKoba_OxA-21778", 35100, 600)
   {
color="Green";
    Outlier("General", 0.05);
   };

R_Date("Cobrante_OxA-32505", 35150, 650)
    {
```

```
color="Green";
     Outlier("General", 0.05);
    };
     R_Date("ElCuco_OxA-32502", 35050, 650)
     {
color="Green";
       Outlier("General", 0.05);
     };
R_Date("Covalejos_OxA-32511", 35250, 700)
     {
color="Green";
       Outlier("General", 0.05);
     };
R_Date("Covalejos_OxA-32512", 34850, 600)
     {
color="Green";
       Outlier("General", 0.05);
     };
     R_Date("Covalejos_OxA-32513", 35150, 650)
     {
color="Green";
       Outlier("General", 0.05);
     };
     R_Date("Covalejos_OxA-32549", 34350, 600)
     {
color="Green";
       Outlier("General", 0.05);
     };
   R_Date("LabekoKoba_OxA-21767", 34750, 600)
   {
color="Green";
    Outlier("General", 0.05);
   };
   R_Date("LabekoKoba_OxA-21779", 34650, 600)
   {
color="Green";
    Outlier("General", 0.05);
   };
   R_Date("Gatzarria_OxA-22555",34400,550)
   {
color="Green";
    Outlier(0.05);
   };
   R_Date("Gatzarria_OxA-22556",34250,550)
   {
color="Green";
    Outlier(0.05);
   };
   R_Date("LaGüelga_COL2579.2.1",34500,76.)
   {
color="Green";
    Outlier(0.05);
   };
   R_Date("LaGüelga_COL2578.2.1",34355,751)
   {
color="Green";
    Outlier(0.05);
   };
   R_Date("LaGüelga_COL2578.1.1",34164,273)
```

```
   {
color="Green";
    Outlier(0.05);
   };
   R_Date("LaGüelga_COL2579.1.1",34121,266)
   {
color="Green";
    Outlier(0.05);
   };
   R_Date("LaGüelga_OxA-30810",33900,550)
   {
color="Green";
    Outlier(0.05);
   };
   R_Date("Gatzarria_OxA-22553",33800,550)
   {
color="Green";
    Outlier(0.05);
   };
   R_Date("LaGüelga_Beta-377233",33610,220)
   {
color="Green";
    Outlier(0.05);
   };
    R_Combine("comb:Ekain")
    {
color="Green";
     R_Date("OxA-32423 Ekain", 31140, 400)
     {
color="Green";
      Outlier("SSimple", 0.05);
     };
     R_Date("OxA-32424 Ekain", 31100, 400)
     {
color="Green";
      Outlier("SSimple", 0.05);
     };
     };
    R_Date ("Aitz_OxA_34932", 31130, 390)
    {
color="Green";
     Outlier("General", 0.05);
    };
  };
   Boundary("End Aurignacian");
  };
 };
};
```

Name	Unmodelled (BP)						Modelled (BP)						Indices				
Amodel 91.2																	
Aoverall 86.3"																	
	from	to	%	from	to	%	from	to	%	from	to	%	Acomb	A	L	P	C
Boundary End Aurignacian							39069	35422	68.268949	39211	34901	95.449974					99.3
R_Date Aitz_OxA_34932	36031	35195	68.268949	36245	34690	95.449974	40684	35449	68.268949	42243	35257	95.449974		34.1		30.8	99.4
R_Date OxA-32424 Ekain	36030	35153	68.268949	36237	34662	95.449974											
R_Date OxA-32423 Ekain	36046	35197	68.268949	36266	34682	95.449974											
R_Combine comb:Ekain	35898	35244	68.268949	36128	34826	95.449974	39220	35751	68.268949	39390	35283	95.449974		28.2			99.8
R_Date LaGÃ¼elga_Beta-377233	39140	38180	68.268949	39240	37648	95.449974	39247	38637	68.268949	39381	37798	95.449973		104.3		95.9	99.9
R_Date Gatzarria_OxA-22553	39415	37815	68.268949	40051	37090	95.449974	39753	38585	68.268949	40475	37531	95.449974		102.5		95.4	99.9
R_Date LaGÃ¼elga_OxA-30810	39602	37863	68.268949	40237	37216	95.449974	39820	38642	68.268949	40515	37604	95.449974		105.5		95.5	99.9
R_Date LaGÃ¼elga_COL2579.1.1	39643	39066	68.268949	39928	38392	95.449974	39623	39091	68.268949	39963	38511	95.449974		105		94.2	99.9
R_Date LaGÃ¼elga_COL2578.1.1	39667	39108	68.268949	39981	38451	95.449974	39657	39121	68.268949	40027	38551	95.449974		103.8		94.2	99.9
R_Date LaGÃ¼elga_COL2578.2.1	40506	38546	68.268949	41099	37440	95.449974	40483	38961	68.268949	41240	37808	95.449974		109.7		95.9	99.9
R_Date LaGÃ¼elga_COL2579.2.1	39743	39484	68.268949	39880	39366	95.449974	39746	39482	68.268949	39889	39361	95.449974		100		95	100
R_Date Gatzarria_OxA-22556	40121	38594	68.268949	40615	37640	95.449974	40124	38919	68.268949	40873	38068	95.449974		109.8		95.4	99.9
R_Date Gatzarria_OxA-22555	40395	39055	68.268949	40789	37817	95.449974	40297	39111	68.268949	40979	38353	95.449974		107.8		95.4	99.9
R_Date LabekoKoba_OxA-21779	40517	39270	68.268949	41168	38269	95.449974	40486	39293	68.268949	41161	38645	95.449973		106.5		97.2	99.9
R_Date LabekoKoba_OxA-21767	40560	39358	68.268949	41226	38507	95.449974	40540	39370	68.268949	41227	38815	95.449974		105.2		97.2	99.9
R_Date Covalejos_OxA-32549	40425	38853	68.268949	40805	37661	95.449974	40322	39050	68.268949	41021	38178	95.449974		111		97	99.9

R_Date Covalejos_OxA-32513	40922	39704	68.268949	41569	39161	95.449974	40932	39707	68.268949	41539	39165	95.449974	102.9	97.2	99.9
R_Date Covalejos_OxA-32512	40622	39446	68.268949	41311	38770	95.449974	40610	39454	68.268949	41279	38957	95.449974	104.1	97.2	99.9
R_Date Covalejos_OxA-32511	41036	39749	68.268949	41750	39209	95.449974	41046	39758	68.268949	41718	39216	95.449974	103	97.2	99.9
R_Date ElCuco_OxA-32502	40841	39608	68.268949	41569	39042	95.449974	40844	39609	68.268949	41482	39080	95.449974	103.4	97.2	99.9
R_Date Cobrante_OxA-32505	40922	39704	68.268949	41569	39161	95.449974	40924	39702	68.268949	41539	39167	95.449974	102.9	97.2	99.9
R_Date LabekoKoba_OxA-21778	40836	39693	68.268949	41380	39195	95.449974	40829	39683	68.268949	41390	39191	95.449974	102.7	97.2	99.9
R_Date OxA-21840 Labeko	40998	39790	68.268949	41608	39251	95.449974									
R_Date OxA-21793 Labeko	41114	39914	68.268949	41713	39391	95.449974									
R_Combine comb:Labeko	40929	40008	68.268949	41282	39576	95.449974	40937	39999	68.268949	41303	39558	95.449974	99.2		99.9
R_Date Gatzarria_OxA-22554	41891	40840	68.268949	42218	40133	95.449974	41888	40818	68.268949	42197	40069	95.449974	101.7	95.7	99.9
R_Date LabekoKoba_OxA-X-2314-43	42004	40980	68.268949	42347	40270	95.449974	41985	40971	68.268949	42285	40193	95.449974	102.5	95.8	99.9
R_Date LabekoKoba_OxA-21766	42150	41192	68.268949	42514	40531	95.449974	42098	41174	68.268949	42398	40473	95.449974	103.8	95.7	99.9
R_Date Isturitz_AA69181	42165	41115	68.268949	42581	40359	95.449974	42105	41106	68.268949	42420	40290	95.449974	104.4	95.8	99.9
R_Date Isturitz_AA69185	42164	41327	68.268949	42480	40793	95.449974	42108	41299	68.26895	42384	40717	95.449974	103.4	95.5	99.9
R_Date Isturitz_AA69179	42753	40457	68.268949	44635	39212	95.449974	42240	40653	68.268949	42554	39417	95.449974	117.6	96.5	99.9
R_Date Isturitz_OxA-23432	42202	41282	68.268949	42568	40653	95.449974	42132	41258	68.26895	42425	40597	95.449974	104.3	95.6	99.9
R_Date Isturitz_OxA-23434	42202	41282	68.268949	42568	40653	95.449974	42134	41260	68.268949	42427	40589	95.449974	104.3	95.6	99.9
R_Date Isturitz_OxA-23436	42400	41415	68.268949	42925	40775	95.449974	42239	41389	68.268949	42543	40714	95.449974	106.2	95.3	99.9
R_Date Isturitz_AA69180	43188	40392	68.268949	45941	39116	95.449974	42287	40661	68.268949	42606	39395	95.449974	121	96.5	99.9
R_Date LaGÃ¼elga_OxA-30809	42365	41745	68.268949	42567	41294	95.449974	42268	41655	68.268949	42461	41221	95.449974	100.1	94.7	99.9
R_Date Isturitz_AA69183	42455	41630	68.268949	42831	41125	95.449974	42285	41544	68.268949	42558	41025	95.449974	102.7	94.9	99.9
R_Date Isturitz_OxA-23435	42445	41469	68.268949	42994	40843	95.449974	42262	41431	68.268949	42581	40780	95.449974	105.8	95.2	99.9

R_Date ElCastillo_OxA-22200	43136	42020	68.268949	44283	41583	95.449974	42479	41776	68.268949	42869	41109	95.449974		86.5		93.6	99.9	
Phase Start Aurignacian																		
Boundary Start Aurignacian							42636	42080	68.268949	43070	41825	95.449974					99.7	
Sequence																		
N(0	2)	-2.06	2.06	68.268949	-4	4	95.449974											100
Outlier_Model SSimple							...	648	68.268948	...	1010	95.449973					99.9	
U(0	4)	3.99E-17	4	68.268949	3.99E-17	4	95.449974	3.164	4	68.268949	5.38E-17	4	95.449973		100			99.5
T(5)	-1.14	1.14	68.268949	-2.65	2.65	95.449974											99.5	
Outlier_Model General							-166	6154	68.26895	-1735	6947	95.449974					99	
Curve IntCal20																		

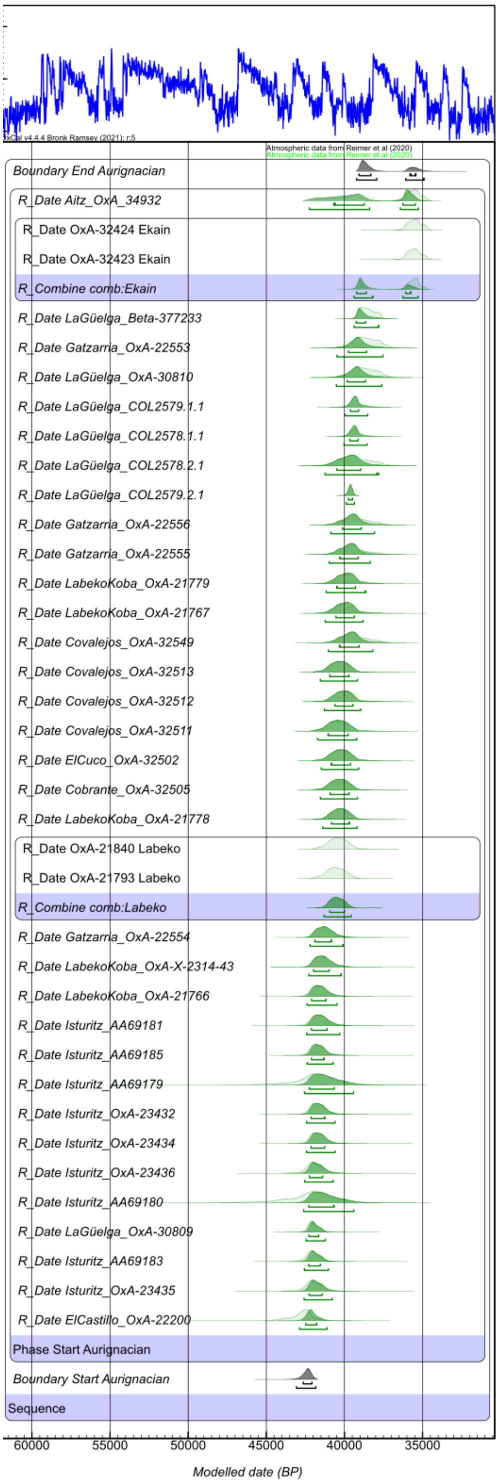

Figure 8. Plot of dated radiocarbon dates from Aurignaican assemblages in the Eurosiberian region once the oldest and youngest dates were removed.

1.5. Summary

Validation and sensitivity tests

When the oldest and youngest dates of each techno-complex is removed from the model, the start and end boundaries of all cultures remains similar, with the exception of the Châtelperronian model. It can be observed that the start and end boundaries of the Châtelperronian techno-complex are sensitive to the oldest and youngest dates (Table 1). In this regard, it should be noted that the TL date obtained in Arranblatxa has a large standard error (2,900 years); furthermore, the date obtained from level Xa of Ekain might be problematic. In this connection, it has been proposed that "*The new date for Level Xa is 34 ka uncal BP, which is far too young for the regional Châtelperronian, dated at 37-38k uncal BP in nearby Labeko Koba. The location of the bone sampled at the site and the significant presence of cave bears do not rule out the possibility of admixture caused either by those carnivores or by other post-depositional processes not identified during the excavation of Ekain"*[7]. For all these reasons, we consider more parsimounious to exclude these oldest (Arranbatza) and youngest (Labeko Koba) dates of the sequence for the Câtelperronian. On the other hand, it can be observed that if the radiocarbon dates performed on shells from level X of El Cuco are included in the model, the end of the Mousterian changes in 480 years. Thus, to avoid the uncertainties related with the reservoir effect, we decided to exclude these dates from the regional model. After performing the sensitivity test, the start and end boundaries of all techno-complexes remain similar (Table 2).

Eurosiberian region

Culture	Boundary	Model	68.2% prob.		95.54% prob.		A. model
			From	to	From	to	
Mousterian	End	a. All dates	45.68	44.44	46.53	43.58	91
		b. Excluding dates obtained from shells	46.5	45.08	47.08	44.06	76.3
		c. Excluding the oldest and youngest dates	46.43	45.15	46.92	44.18	88.8
Châtelperronian	Start	a. All dates	43.45	42.16	45.02	42.00	79.8
		b. Excluding the oldest and youngest dates	42.89	42.17	43.83	42.02	104.9
	End	a. All dates	42.26	39.56	42.33	38.44	79.8
		b. Excluding the oldest and youngest dates	42.22	41.72	42.34	41.02	104.9
Aurignacian	Start	a. All dates	42.84	42.65	43.33	42.05	106.8
		b. Excluding the oldest and youngest dates	42.63	42.08	43.07	41.82	91.2
	End	a. All dates	37.86	35.14	36.01	34.73	106.8
		b. Excluding the	39.06	35.42	39.21	34.90	91.2

		oldest and youngest dates

Table 1. Results of the 68.2% and 95.4% PDF rage of the boundaries between each technocomplex.

Eurosiberian region

**Culture**	**Boundary**	**Model**	**68.2% prob.**		**95.54% prob.**		**A. model**
			From	to	From	to	
Mousterian	End	1	46.5	45.08	47.08	44.06	76.3
		2	46.48	45.05	47.08	44.02	77.1
		3	46.55	45.12	47.09	44.07	76.5
		4	46.39	45.12	46.93	44.12	89.1
Châtelperronian	Start	1	42.89	42.17	43.83	42.02	104.9
		2	42.77	42.10	43.78	41.99	100.1
		3	42.81	42.15	43.81	42.02	111.6
		4	42.91	42.12	43.88	42.06	102.7
	End	1	42.22	41.72	42.34	41.02	104.9
		2	42.27	41.69	42.24	41.16	100.1
		3	42.21	41.77	42.22	41.09	111.6
		4	42.19	41.66	42.42	41.07	102.7
Aurignacian	Start	1	42.84	42.65	43.33	42.05	106.8
		2	42.82	42.36	43.32	42.05	106.7
		3	42.84	42.26	43.33	42.04	107.3
		4	42.81	42.64	43.33	42.02	106
	End	1	35.80	35.15	36.02	34.73	106.8
		2	35.81	35.15	36.05	34.71	106.7
		3	35.79	35.09	36.04	34.72	107.3
		4	35.84	35.12	36.10	34.66	106

Table 2. Results obtained when the Bayesian age model for the Mousterian, Châtelperronian and Aurignacian techno-complesexes if the each model is run four different times.

2. Mediterranean region

2.1. Excluded sites, levels or dates

- Jarama VI

Different radiocarbon dates were recovered from Jarama VI.2 ranging from 29,500±2,700 BP to > 52,000 BP[8,9]. However, it has been claimed that no bones from level VI.2 preserved enough nitrogen content, which hampers dating the final Mousterian in this site [8]. Moreover, independent ages by means of dating the sediments using infrared stimulated luminescence revealed that level VI.2 was close to, or beyond the limit of radiocarbon daing (> 50 ka cal BP) [10]. For all these reasons the level VI.2 of the Jarama site was not included.

- Portalón de la Tejadilla

Portalón de la Tejadilla provides valuable insights into the fauna composition in the inner areas of Iberia during the MIS 3. However, the lithic remains recovered

are significantly low, not completely diagnostic, and they do not indicate clear occupation horizons, as the authors acknowledge: "*the scarce lithic remains that have been recovered here are either compatible with Middle Palaeolithic technology or they are undiagnostic. Although these scarce pieces are not indicative of occupation horizons, they could indicate the scattered presence of human populations. Nevertheless, the lithic evidence, found in the bioturbated unit, could reflect a slightly older human presence from our datings based on the dates of the disappearance of the Mousterian in central Iberia and adjacent areas*" [11]

- Cova Gran

We incorporated the Mousterian and Aurignacian levels of Cova Gran in the regional Bayesian age models performed in this study. However, we discarded the dates that the authors consider less reliable. Radiocarbon dates obtained from layer SID were interpreted as minimum ages, and the authors stated: "*We suggest the demise of the Mousterian tradition seen in the Cova Gran sequence occurred during the temporal range provided by the Beta-224299 sample from S1B, suggesting a minimum age of 42 ka cal BP. Similarly, we assign the emergence of the EUP to the temporal range provided by AA 68834 from 497D, treated with ABOX protocol, indicating a date ca. 39–38 ka cal BP, while samples treated with AAA and ABA protocols indicate a minimum age ca. 37.5–36 ka cal BP*" [12] Following the stratigraphic and chronometric analyses performed by these authors[12], we included the dates that they consider indicative for the end of the Mousterian and the arrival of the Aurignacian to the site.

- Carihuela

New radiocarbon dates, obtained from coprolites, bulk sediments, and bone remains have been recently obtained from Carihuela [13] The youngest dates were obtained from bulk sediments, so it has been proposed that these samples are probably biased by younger introduced carbon during the soil formation [14]. Despite the authors of this study suggest that evidence recovered from Carihuela demonstrates the persistence of Neanderthal populations in this region in the late MIS 3, they acknowledge the difficulties of offering a precise and reliable chronology for the last Mousterian occupation and claim that more dating programs are required [13].

- El Niño

Two radiocarbon dates obtained from bone remains (UGAMS-7737 and UGAMS-7739) suggest that levels VI and III from El Niño site were accumulated at the end of the MIS 3 [15]. However, the low nitrogen content hampers the chronological demarcation of the Mousterian levels from El Niño and, as the authors acknowledge, these dates should not be considered reliable [15].

- Zafarraya

Zafarraya was considered a key site for the last presence of Neanderthals in southern Iberia. U-series, ESR and radiocarbon dates demonstrate that Neanderthals occupied this sie in the late MIS 3[16], but the temporal range of the

occupation (30-46 ka cal BP)[16] is large. One bone from the Mousterian level (OxA-8999, 33,300± 1200 BP) was re-dated to > 46,700 BP[8] and, as explained in details in [17], most of the bone had low nitrogen content, so they consider that "…*this allows us to remove, with confidence, the remaining evidence for a post-42 ka cap BP Neanderthal occupation*" [17]

- Sima de las Palomas

Two radiocarbon dates (OxA-15423 and OxA-10666) from burnt bone remains recovered in the Mousterian level of Sima de las Palomas provided ages of 35030±270 and 34450±600 [18,19]. However, luminescence and U-series dates set an age of 45.3 BP in the base of this level [19].

- Gorham

Gorham cave is commonly used as evidence that Mousterian survived until c.30-32 ka BP. In level IV, 22 radiocarbon dates on charcoal, some of which were taken from a hearth, have been used to argue for the survival of the Middle Palaeolithic, and thus Neanderthals, until 28 BP[20] Zilhão and Pettitt[21] doubt the reliability of these dates, questioning both the ability of the pre-treatment applied to remove contamination and the association of the charcoal fragments with the small assemblage of lithic tools. Previous analyses had suggested admixture in level IV between Middle and Upper Paleolithic remains[22,23]. Besides the uncertainties regarding the stratigraphic sequence and the level formation, other studies have re-analysed the chronology of level IV and commonly led to the conclusion that "*there is little evidence in the published chronology from Gorham's Cave that Neanderthals survived beyond 40 ka BP*"[24]

- Abrigo del Molino

We excluded two radiocarbon dates from level 2 of Abrigo del Molino (COL2715 and COL2715.3.1) and three radiocarbon dates from levels G and K (COL4018.1.1, COL4019.1.1, COL4020.1.1) because the authors of this study reported a low collagen yield from these samples [25].

- Gruta da Oliveira

Radiocarbon dates obtained from Levels 8 of Gruta da Oliveira (see Supplementary Data 1) provide evidence for the persistence of Neanderthals beyond 41.5 ka cal BP in this region. U-series outcomes suggest that level 8 was formed between 34-40 ka cal BP[26], which is consistent with radiocarbon dates, despite the low chronological precision. However, recent OSL and thermoluminescence dates show that these layers correspond with the MIS5 [27]

- Gruta da Columbeira

Gruta da Columbeira has a poorly defined chronological sequence. In the 80s', this site was considered the evidence for the late survival of Neanderthals in Portugal on the basis of two radiocarbon dates obtained from levels 7 and 8 that reported an age between 26,400 and 28,900 BP[28]. Posterior U-series analyses showed that a

human-modified bone from level 8 was much older (87.1±6.3)[29], so this site is not considered as evidence of late Neanderthal survival in Iberia anymore[30]

- Higeral del Valleja

The latest Mousterian occupation in Higheral de Valleja was found in level V. Radiocarbon dates obtained from small charcoal remain produced an age of 20,780±80, so the authors consider that "*the dated charcoal could conceivably have migrated into lager V from above and could tie in with Solutren occupation layers. One also has to explain the OSL date in layer IV of 33,200±3,100 BP*"[31]. These results suggest stratigraphic inconsistencies and low chronological reliability.

2.2. Supramediterranean region: End Mousterian

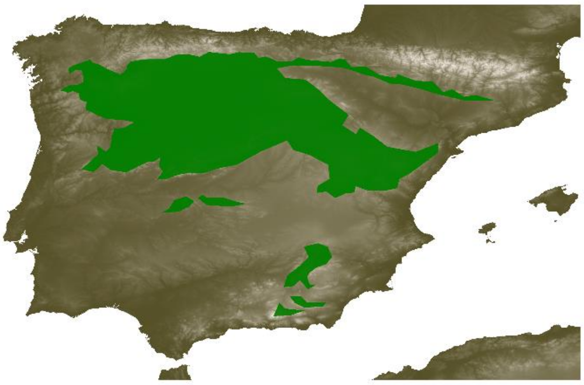

Figure 9. The Iberian Peninsula with the Submediterranean/Supramediterranean biogeographic region shaded in green.

a. **Including all radiocarbon dates**

```
Options()
 {
  Curve("IntCal20","intcal20.14c");
  BCAD=FALSE;
  SD1=TRUE;
  SD2=TRUE;
  ConvergenceData=TRUE;
  kIterations=300;
 };
 Plot()
 {
  Outlier_Model("General",T(5),U(0,4),"t");
  Outlier_Model("SSimple",N(0,2),0,"s");
  Sequence()
  {
```

```
 Boundary("End Mousteria");
 Phase("Mousterian")
 {
  R_Date("AbrigodelMolino_COL2716.1.1", 40100, 1600)
  {
   Outlier("General", 0.05);
  };
  R_Date("AbrigodelMolino_COL2714.1.1", 39500, 600)
  {
   Outlier("General", 0.05);
  };
  R_Date("Casares_COL4208.1.1", 39494, 850)
  {
   Outlier("General", 0.05);
  };
  R_Date("CuevaMillan_GrN-11021", 37600, 700)
  {
   Outlier("General", 0.05);
  };
  R_Date("CuevaMilla_GrN-11161", 37450, 650)
  {
   Outlier("General", 0.05);
  };
 };
 Boundary("Final End Mousterian");
};
};
```

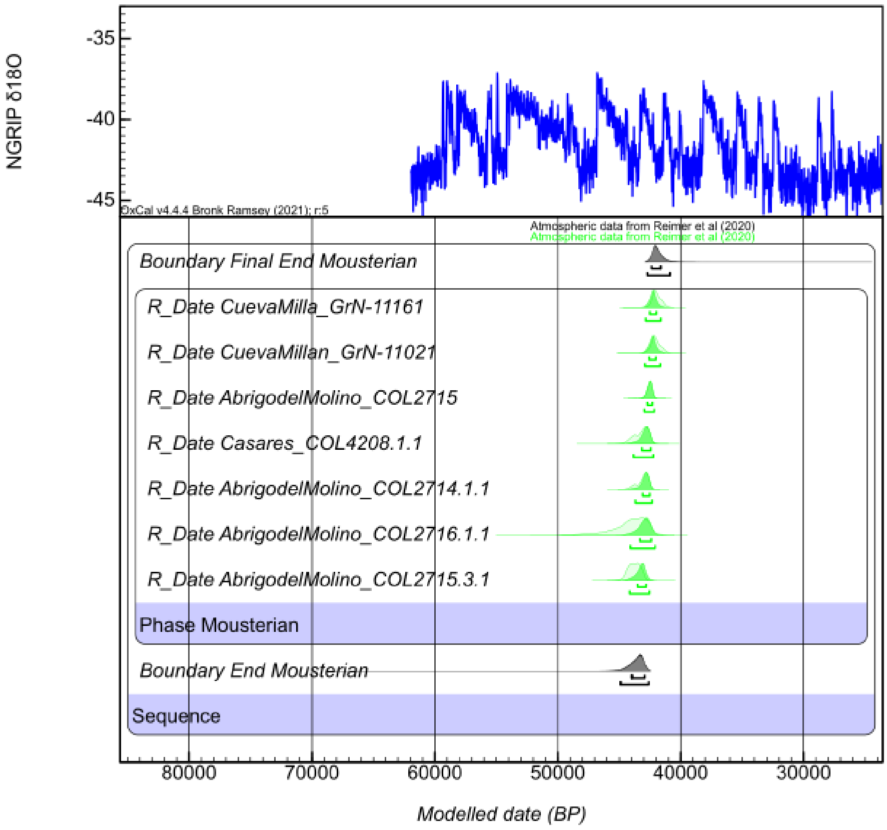

Figure 10. Plot of dated radiocarbon dates from Mousterian assemblages in the Submediterranean region.

**Name**	**Unmodelled (BP)**						**Modelled (BP)**						**Indices**					
Amodel 114.8																		
Aoverall 109.9"																		
	from	to	%	from	to	%	from	to	%	from	to	%	Acomb	A	L	P	C	
Boundary Final End Mousterian							42471	41568	68.268949	42761	40489	95.449974					97.9	
R_Date CuevaMilla_GrN-11161	42356	41665	68.268949	42597	41211	95.449974	42548	41986	68.268949	42810	41575	95.449974		92.1		95.4	99.8	
R_Date CuevaMillan_GrN-11021	42441	41727	68.268949	42733	41239	95.449974	42587	42027	68.268949	42861	41619	95.449974		98.3		95.7	99.9	
R_Date Casares_COL4208.1.1	43809	42440	68.268949	44383	42267	95.449974	42968	42376	68.268949	43724	42182	95.449974		114.5		95.8	99.8	
R_Date AbrigodelMolino_COL2714.1.1	43281	42492	68.268949	44067	42380	95.449974	42953	42449	68.268949	43536	42266	95.449974		111.4		95.8	99.8	
R_Date AbrigodelMolino_COL2716.1.1	44660	42454	68.268949	47310	41921	95.449974	43059	42300	68.268949	44029	42000	95.449974		109.3		95.6	99.8	
Phase Mousterian																		
Boundary End Mousteria							43572	42505	68.268949	45070	42343	95.449974					97	
Sequence																		
N(0	2)	-2.06	2.06	68.268949	-4	4	95.449974											100
Outlier_Model SSimple																	100	
U(0	4)	3.99E-17	4	68.268949	3.99E-17	4	95.449974	5.38E-17	2.696	68.268949	5.38E-17	3.784	95.449974		100			100
T(5)	-1.14	1.14	68.268949	-2.65	2.65	95.449974											99.9	
Outlier_Model General							-145	145	68.268949	-1048	1053	95.449974					100	
Curve IntCal20																		

b. Excluding the oldest and youngest dates

```
Options()
 {
  Curve("IntCal20","intcal20.14c");
  BCAD=FALSE;
  SD1=TRUE;
  SD2=TRUE;
  ConvergenceData=TRUE;
  kIterations=300;
 };
 Plot()
 {
  Outlier_Model("General",T(5),U(0,4),"t");
  Outlier_Model("SSimple",N(0,2),0,"s");
  Sequence()
  {
   Boundary("End Mousteria");
   Phase("Mousterian")
   {
    R_Date("AbrigodelMolino_COL2714.1.1", 39500, 600)
    {
     Outlier("General", 0.05);
    };
    R_Date("Casares_COL4208.1.1", 39494, 850)
    {
     Outlier("General", 0.05);
    };
    R_Date("CuevaMillan_GrN-11021", 37600, 700)
    {
     Outlier("General", 0.05);
    };
   };
   Boundary("Final End Mousterian");
  };
 };
```

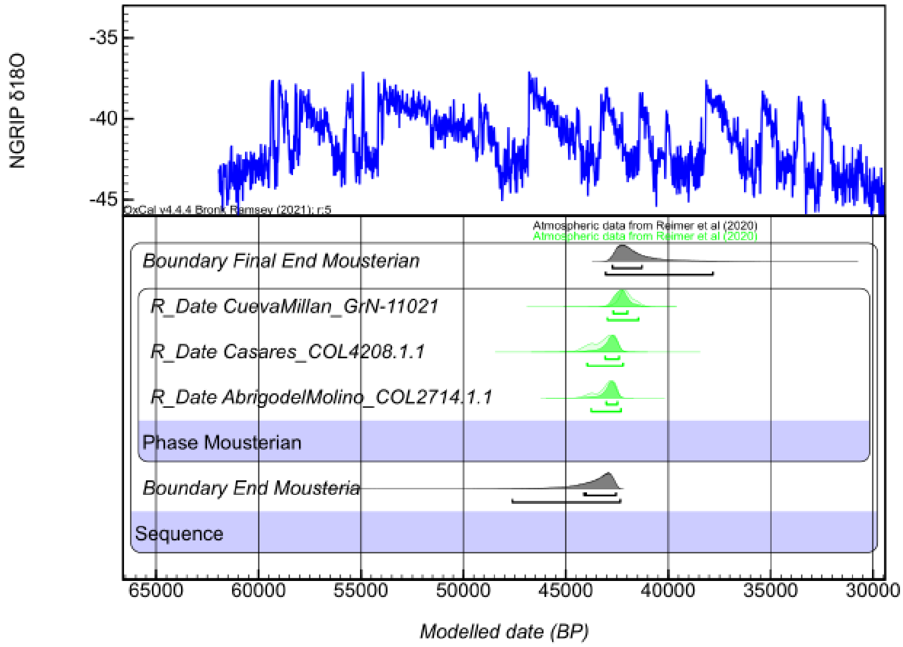

Figure 11. Plot of dated radiocarbon dates from Mousterian assemblages in the Submediterranean region once the oldest and youngest dates were removed.

Name	Unmodelled (BP)						Modelled (BP)						Indices					
Amodel 106.4																		
Aoverall 108"																		
	from	to	%	from	to	%	from	to	%	from	to	%	Acomb	A	L	P	C	
Boundary Final End Mousterian							42718	41286	68.268949	43063	37819	95.449974					97.6	
R_Date CuevaMillan_GrN-11021	42441	41727	68.268949	42733	41239	95.449974	42678	41986	68.268949	42969	41463	95.449974		89.4		95.2	99.5	
R_Date Casares_COL4208.1.1	43809	42440	68.268949	44383	42267	95.449974	43085	42404	68.268949	43958	42217	95.449974		115.4		95.8	99.7	
R_Date AbrigodelMolino_COL2714.1.1	43281	42492	68.268949	44067	42380	95.449974	43031	42481	68.268949	43762	42316	95.449974		113.1		95.8	99.8	
Phase Mousterian																		
Boundary End Mousteria							44105	42551	68.268949	47613	42348	95.449974					96.9	
Sequence																		
N(0	2)	-2.06	2.06	68.268949	-4	4	95.449974											100
Outlier_Model SSimple																	100	
U(0	4)	3.99E-17	4	68.268949	3.99E-17	4	95.449974	0.028	2.824	68.268949	5.38E-17	3.788	95.449974		100			100
T(5)	-1.14	1.14	68.268949	-2.65	2.65	95.449974											99.5	
Outlier_Model General							-166	158	68.268949	-1062	1268	95.449974					99.9	
Curve IntCal20																		

2.2.1. Summary

Validation and sensitivity tests

When the oldest and youngest dates of the Mousterian are removed from the model, the end boundary remains similar regarding the outcomes ontained at 68.2% CI, but the time interval for the end boundary at 95.4% CI is significantly wider (Table 3). This is due to the low sample size in this region. After removing the oldest and youngest dates, only three radiocarbon determinations are used in the model. Therefore, removing the oldest and youngest dates has a signifcant effect on the chronology obtained. However, it is worth noting that the chronolgy for the end of the Mousterian should be considered older than 40 ka cal BP because all individual radiocarbon determinations recovered in this regions are older than 40 ka cal BP (Figure 9) at 95%CI.

Suprasmediterranean

Culture	Boundary	Model	68.2% prob.		95.54% prob.		A. model
			From	to	From	to	
Mousterian	End	a. All dates	42.47	41.56	42.76	40.48	114.8
		c. Excluding the oldest and youngest dates	42.71	41.28	43.06	37.81	106.4

Table 3. Results of the 68.2% and 95.4% PDF rage of the Mousterian techno-complex in the Supramediterranean region.

Submediterranean region

Culture	Boundary	Model	68.2% prob.		95.54% prob.		A. model
			From	to	From	to	
Mousterian	End	1	42.47	41.56	42.76	40.48	114.8
		2	42.51	41.55	42.75	40.50	115.1
		3	42.48	41.54	42.76	40.47	109.4
		4	42.45	41.59	42.78	40.49	110.2

Table 4. Results obtained when the Bayesian age model for the Mousterian are replicated 4 different times.

2.3. Mesomediterranean

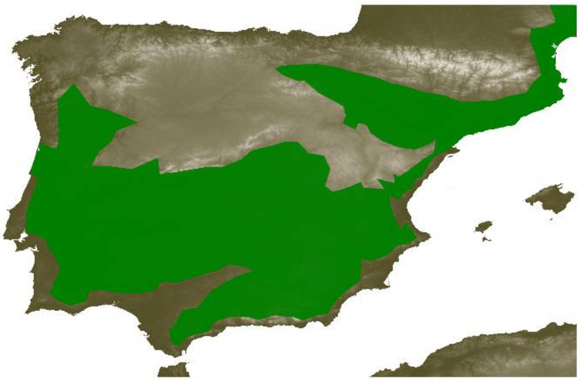

Figure 12. The Iberian Peninsula with the Mesomediterranean biogeographic region shaded in green.

2.3.1. End Mousterian

a. All dates

```
Options()
 {
  Curve("IntCal20","intcal20.14c");
  BCAD=FALSE;
  SD1=TRUE;
  SD2=TRUE;
  ConvergenceData=TRUE;
  kIterations=300;
 };
Plot()
{
  Outlier_Model("General",T(5),U(0,4),"t");
Outlier_Model("SSimple",N(0,2),0,"s");
  Sequence()
  {
    Boundary("End Mousterian");
    Phase("End Mousterian")
{
R_Date("LosMoros_ GrN-12809", 46500, 4400)
     {
color="Green";
        Outlier("General", 0.05);
     };
R_Date("PeñaMiel_GrN-12123", 45500, 1400)
     {
color="Green";
        Outlier("General", 0.05);
     };
R_Date("Aguilon_MAMS-28122", 44560, 480)
     {
color="Green";
        Outlier("General", 0.05);
```

```
    };
Age("AbricRomani_NZA-2315", N (44560, 480))
    {
color="Green";
      Outlier("General", 0.05);
    };
    R_Date("Arbreda_OxA-21702", 44400, 1900)
    {
color="Green";
      Outlier("General", 0.05);
    };
  Age("FozdoEnxarrique_052201 PCI", N(44000, 3000))
    {
color="Green";
      Outlier("General", 0.05);
    };
Age("FozdoEnxarrique_052202 PC2", N(43000, 3000))
    {
color="Green";
      Outlier("General", 0.05);
    };
Age("SaltodoBoi_172209", N(42900, 1900))
    {
color="Green";
      Outlier("General", 0.05);
    };

R_Date("Teixoners_S-EVA26854", 42250, 359)
    {
color="Green";
      Outlier("General", 0.05);
    };
R_Date("Teixoners_S-EVA27839", 42020, 370)
    {
color="Green";
      Outlier("General", 0.05);
    };
R_Date("Teixoners_S-EVA26774", 41560, 337)
    {
color="Green";
      Outlier("General", 0.05);
    };
R_Date("Aguilon_Beta-313364", 41510, 550)
    {
color="Green";
      Outlier("General", 0.05);
    };
R_Date("LapadoPicareiro_UG-07769", 41480, 220)
    {
color="Green";
      Outlier("General", 0.05);
    };
R_Date("Teixoners_S-EVA26769", 41270, 327)
    {
color="Green";
      Outlier("General", 0.05);
    };
R_Date("Teixoners_S-EVA27840", 40610, 340)
    {
```

```
color="Green";
      Outlier("General", 0.05);
    };
R_Date("Elsermitons_GrA-33813", 40580, 550)
    {
color="Green";
      Outlier("General", 0.05);
    };
R_Date("PeñaMiel_OxA-5519", 40300, 1600)
    {
color="Green";
      Outlier("General", 0.05);
    };
R_Date("LapadoPicareiro_Wk-28844", 40100, 1200)
    {
color="Green";
      Outlier("General", 0.05);
    };
R_Date("CollVerdaguer_OxA-23636", 39950, 650)
    {
color="Green";
      Outlier("General", 0.05);
    };
Age("SaltodoBoi_172210", N(39500, 1800))
    {
color="Green";
      Outlier("General", 0.05);
    };
    R_Date("Arbreda_OxA-21704", 39200, 1000)
    {
color="Green";
      Outlier("General", 0.05);
    };
R_Date("RocadelsBous_BAA-6481", 38800, 120)
    {
color="Green";
      Outlier("General", 0.05);
    };
R_Date("CovaBeneito_AA-1387", 38800, 1900)
    {
color="Green";
      Outlier("General", 0.05);
    };
    R_Date("CovaGran_Beta-224299", 38640, 440)
    {
color="Green";
      Outlier("General", 0.05);
    };
R_Date("FuentesdeSanCristobal__OxA-19934", 38550, 450)
    {
color="Green";
      Outlier("General", 0.05);
    };
    R_Date("Arbreda_OxA-19994", 38350, 400)
    {
color="Green";
      Outlier("General", 0.05);
    };
    R_Date("CollVerdaguer_OxA-23638", 38000, 550)
```

```
    {
color="Green";
      Outlier("General", 0.05);
    };
    R_Date("CollVerdaguer_OxA-23637", 37600, 550)
    {
color="Green";
      Outlier("General", 0.05);
    };
    R_Date("FuentesdeSanCristobal_OxA-33904", 37330, 490)
    {
color="Green";
      Outlier("General", 0.05);
    };
    R_Date("Arbreda_OxA-21662", 37300, 800)
    {
color="Green";
      Outlier("General", 0.05);
    };
    R_Date("GrutadoCaldeirao_MAMS-41871", 36490, 390)
    {
color="Green";
      Outlier("General", 0.05);
    };
R_Date("FuentesdeSanCristobal_OxA-36200", 36200, 350)
    {
color="Green";
      Outlier("General", 0.05);
    };
    R_Date("GrutadoCaldeirao_MAMS-41874", 33810, 290)
    {
color="Green";
      Outlier("General", 0.05);
    };
R_Date("GrutadoCaldeirao_MAMS-41876", 32890, 260)
    {
color="Green";
      Outlier("General", 0.05);
    };
   };
   Boundary("Final End Mousterian");
  };
 };
};
```

Name	Unmodelled (BP)						Modelled (BP)						Indices					
Amodel 48.8																		
Aoverall 27.1"																		
	from	to	%	from	to	%	from	to	%	from	to	%	Acomb	A	L	P	C	
Boundary Final End Mousterian	Mesomediterranean Region							41671	41045	68.268949	41983	40316	95.449974					98.8
R_Date GrutadoCaldeirao_6_MAMS-41876	37685	36875	68.268949	38525	36552	95.449974	43922	41334	68.268949	45998	38801	95.449974		6		2.7	99.5	
R_Date GrutadoCaldeirao_5_MAMS-41874	39352	38372	68.268949	39521	37674	95.449974	43988	41362	68.26895	45868	38958	95.449973		9.1		3.3	99.2	
R_Date FuentesdeSanCristobal_G_OxA-36200	41560	40950	68.268949	41874	40679	95.449974	41853	41295	68.268949	42273	40718	95.449974		80.3		93.7	99.5	
R_Date GrutadoCaldeirao_M_MAMS-41871	41796	41199	68.268949	42044	40911	95.449974	41926	41416	68.268949	42147	41069	95.449974		101.3		96.6	99.6	
R_Date Arbreda_I_OxA-21662	42319	41442	68.268949	42730	40912	95.449974	42339	41599	68.268949	42780	41179	95.449974		110.8		97.8	99.9	
R_Date FuentesdeSanCristobal_F_OxA-33904	42262	41717	68.268949	42401	41331	95.449974	42259	41755	68.268949	42431	41373	95.449974		107.4		97.9	99.9	
R_Date CollVerdaguer_I_OxA-23637	42375	41851	68.268949	42569	41404	95.449974	42371	41866	68.268949	42605	41437	95.449974		106		98	99.9	
R_Date CollVerdaguer_I_OxA-23638	42494	42049	68.268949	42801	41756	95.449974	42498	42046	68.268949	42829	41723	95.449974		103.6		98	100	
R_Date Arbreda_I_OxA-19994	42549	42238	68.268949	42750	42095	95.449974	42553	42235	68.268949	42778	42074	95.449974		103.2		98.2	99.9	
R_Date FuentesdeSanCristobal_G_OxA-19934	42650	42291	68.268949	42875	42139	95.449974	42655	42289	68.268949	42910	42110	95.449974		103		98	100	
R_Date CovaGran_SIB_Beta-224299	42681	42326	68.268949	42902	42176	95.449974	42685	42322	68.268949	42938	42150	95.449974		103		98	100	
R_Date CovaBeneito_D1_AA-1387	44523	41635	68.268949	48047	40382	95.449974	44105	41958	68.268949	45245	41301	95.449974		120.9		97.8	99.9	
R_Date RocadelsBous_a_BAA-6481	42618	42460	68.268949	42709	42392	95.449974	42620	42459	68.268949	42722	42382	95.449974		103.3		98.3	100	
R_Date Arbreda_I_OxA-21704	43795	42283	68.268949	44504	42037	95.449974	43803	42283	68.26895	44507	42034	95.449974		103.6		98	99.9	
N(39500	1800)	37646	41354	68.268949	35900	43100	95.449974	41246	42776	68.268949	40688	44414	95.449974		52.1		95	99.8
#¿NOMBRE?							1950	1951	68.268949	1950	1951	95.449974					100	
SaltodoBoi_c5d_172210	41354	37646	68.268949	43100	35900	95.449974	42776	41246	68.268949	44414	40688	95.449974					99.8	

R_Date CollVerdaguer_I_OxA-23636	43858	42771	68.268949	44336	42544	95.449974	43857	42771	68.268949	44374	42522	95.449974		103		98	99.9	
R_Date LapadoPicareiro_JJ_Wk-28844	44287	42690	68.268949	45515	42242	95.449974	44243	42721	68.268949	45074	42274	95.449974		107.5		97.9	99.9	
R_Date PeÃ±aMiel_e_OxA-5519	44783	42521	68.268949	47446	42029	95.449974	44491	42701	68.268949	45516	42126	95.449974		117		97.8	99.9	
R_Date Elsermitons_IV_GrA-33813	44129	43179	68.268949	44506	42895	95.449974	44136	43174	68.268949	44541	42871	95.449974		103.1		97.9	99.9	
R_Date Teixoners_III_S-EVA27840	44045	43278	68.268949	44336	43055	95.449974	44053	43273	68.268949	44363	43030	95.449974		103		98	99.9	
R_Date Teixoners_III_S-EVA26769	44570	44024	68.268949	44720	43385	95.449974	44576	44017	68.268949	44739	43351	95.449974		103.3		98	99.9	
R_Date LapadoPicareiro_JJ_UG-07769	44595	44271	68.268949	44765	44040	95.449974	44596	44267	68.268949	44795	44000	95.449974		103.3		98	100	
R_Date AguilonP5_e_Beta-313364	44895	43985	68.268949	45274	43274	95.449974	44847	43984	68.268949	45166	43241	95.449974		106.4		98	99.9	
R_Date Teixoners_III_S-EVA26774	44702	44240	68.268949	45050	43893	95.449974	44697	44236	68.268949	45062	43503	95.449974		104.5		98	99.9	
R_Date Teixoners_III_S-EVA27839	45005	44480	68.268949	45405	44277	95.449974	44935	44460	68.268949	45351	44208	95.449974		108.3		97.8	99.9	
R_Date Teixoners_III_S-EVA26854	45158	44598	68.268949	45538	44418	95.449974	45038	44548	68.268949	45485	44336	95.449974		107.1		97.4	99.8	
N(42900	1900)	40943	44857	68.268949	39100	46700	95.449974	41760	44230	68.268949	41152	45408	95.449974		119.2		95	99.9
#¿NOMBRE?							1950	1951	68.268949	1950	1951	95.449974					100	
SaltodoBoi_c5d_172209	44857	40943	68.268949	46700	39100	95.449974	44230	41760	68.268949	45408	41152	95.449974					99.9	
N(43000	3000)	39910	46090	68.268949	37000	49000	95.449974	41800	44500	68.268949	41080	45820	95.449974		129.3		95	99.9
#¿NOMBRE?							1950	1951	68.268949	1950	1951	95.449974					100	
FozdoEnxarrique_T6_052202 PC2	46090	39910	68.268949	49000	37000	95.449974	44500	41800	68.268949	45820	41080	95.449974					99.9	
N(44000	3000)	40910	47090	68.268949	38000	50000	95.449974	42020	44870	68.268949	41150	45980	95.449974		128.3		95	99.9
#¿NOMBRE?							1950	1951	68.268949	1950	1951	95.449974					100	
FozdoEnxarrique_T6_052201 PCI	47090	40910	68.268949	50000	38000	95.449974	44870	42020	68.268949	45980	41150	95.449974					99.9	
R_Date Arbreda_I_OxA-21702	49213	44955	68.268949	54674	44320	95.449974	45739	44213	68.268949	46795	42996	95.449974		71.5		90.4	99.1	
N(44560	480)	44065.6	45054.4	68.268949	43600	45520	95.449974	44056	44934.4	68.268949	43571.2	45347.2	95.449974		104.4		95	99.9

#¿NOMBRE?							1950	1951	68.2689<br>49	1950	1951	95.4499<br>74					10<br>0	
AbricRomani_H_ NZA-2315	4505<br>4	44066	68.2689<br>49	45520	43600	95.4499<br>74	44934	4405<br>6	68.2689<br>49	45347	43571	95.4499<br>74					99.<br>9	
R_Date Aguilon_P5_MAMS-28122	4736<br>8	46187	68.2689<br>49	48040	45936	95.4499<br>74	46600	4283<br>7	68.2689<br>49	46824	41563	95.4499<br>74		30.5		33.<br>6	97	
R_Date PeÃ±aMiel_e_GrN-12123	4964<br>7	46237	68.2689<br>49	54359	45308	95.4499<br>73	46500	4388<br>9	68.2689<br>49	46801	41659	95.4499<br>74		26.1		53.<br>3	98	
R_Date LosMoros_ GrN-12809	5431<br>6	46299	68.2689<br>49	...	44903	95.4499<br>74	45543	4342<br>7	68.2689<br>49	46653	42656	95.4499<br>74		45.9		91.<br>1	99.<br>2	
Phase End Mousterian																		
Boundary End Mousterian Mesomediterranean Iberia							46514	4469<br>7	68.2689<br>49	47102	44602	95.4499<br>74					97.<br>1	
Sequence																		
N(0	2)	-2.06	2.06	68.2689<br>49	-4	4	95.4499<br>74											10<br>0
Outlier_Model SSimple							-2	3	68.2689<br>49	-2	3	95.4499<br>74					10<br>0	
U(0	4)	3.99E-<br>17	4	68.2689<br>49	3.99E-<br>17	4	95.4499<br>74	3.42<br>4	3.848	68.2689<br>49	3.22	4	95.4499<br>74		100			98.<br>5
T(5)	-1.14	1.14	68.2689<br>49	-2.65	2.65	95.4499<br>74											99.<br>4	
Outlier_Model General							-4626	6613	68.2689<br>49	-6002	7800	95.4499<br>74					96.<br>2	
Curve IntCal20																		

b. Excluding outliers

```
Options()
 {
  Curve("IntCal20","intcal20.14c");
  BCAD=FALSE;
  SD1=TRUE;
  SD2=TRUE;
  ConvergenceData=TRUE;
  kIterations=300;
 };
Plot()
{
  Outlier_Model("General",T(5),U(0,4),"t");
Outlier_Model("SSimple",N(0,2),0,"s");
  Sequence()
  {
    Boundary("End Mousterian");
    Phase("End Mousterian")
{
Age("AbricRomani_NZA-2315", N (44560, 480))
     {
color="Green";
       Outlier("General", 0.05);
     };
     R_Date("Arbreda_OxA-21702", 44400, 1900)
     {
color="Green";
       Outlier("General", 0.05);
     };
  Age("FozdoEnxarrique_052201 PCI", N(44000, 3000))
     {
color="Green";
       Outlier("General", 0.05);
     };
Age("FozdoEnxarrique_052202 PC2", N(43000, 3000))
     {
color="Green";
       Outlier("General", 0.05);
     };
Age("SaltodoBoi_172209", N(42900, 1900))
     {
color="Green";
       Outlier("General", 0.05);
     };

R_Date("Teixoners_S-EVA26854", 42250, 359)
     {
color="Green";
       Outlier("General", 0.05);
     };
R_Date("Teixoners_S-EVA27839", 42020, 370)
     {
color="Green";
       Outlier("General", 0.05);
     };
R_Date("Teixoners_S-EVA26774", 41560, 337)
     {
```

```
color="Green";
       Outlier("General", 0.05);
     };
R_Date("Aguilon_Beta-313364", 41510, 550)
     {
color="Green";
       Outlier("General", 0.05);
     };
R_Date("LapadoPicareiro_UG-07769", 41480, 220)
     {
color="Green";
       Outlier("General", 0.05);
     };
R_Date("Teixoners_S-EVA26769", 41270, 327)
     {
color="Green";
       Outlier("General", 0.05);
     };
R_Date("Teixoners_S-EVA27840", 40610, 340)
     {
color="Green";
       Outlier("General", 0.05);
     };
R_Date("Elsermitons_GrA-33813", 40580, 550)
     {
color="Green";
       Outlier("General", 0.05);
     };
R_Date("PeñaMiel_OxA-5519", 40300, 1600)
     {
color="Green";
       Outlier("General", 0.05);
     };
R_Date("LapadoPicareiro_Wk-28844", 40100, 1200)
     {
color="Green";
       Outlier("General", 0.05);
     };
R_Date("CollVerdaguer_OxA-23636", 39950, 650)
     {
color="Green";
       Outlier("General", 0.05);
     };
Age("SaltodoBoi_172210", N(39500, 1800))
     {
color="Green";
       Outlier("General", 0.05);
     };
     R_Date("Arbreda_OxA-21704", 39200, 1000)
     {
color="Green";
       Outlier("General", 0.05);
     };
R_Date("RocadelsBous_BAA-6481", 38800, 120)
     {
color="Green";
       Outlier("General", 0.05);
     };
R_Date("CovaBeneito_AA-1387", 38800, 1900)
```

```
    {
color="Green";
      Outlier("General", 0.05);
    };
    R_Date("CovaGran_Beta-224299", 38640, 440)
    {
color="Green";
      Outlier("General", 0.05);
    };
R_Date("FuentesdeSanCristobal_OxA-19934", 38550, 450)
    {
color="Green";
      Outlier("General", 0.05);
    };
    R_Date("Arbreda_OxA-19994", 38350, 400)
    {
color="Green";
      Outlier("General", 0.05);
    };
    R_Date("CollVerdaguer_OxA-23638", 38000, 550)
    {
color="Green";
      Outlier("General", 0.05);
    };
    R_Date("CollVerdaguer_OxA-23637", 37600, 550)
    {
color="Green";
      Outlier("General", 0.05);
    };
    R_Date("FuentesdeSanCristobal_OxA-33904", 37330, 490)
    {
color="Green";
      Outlier("General", 0.05);
    };
    R_Date("Arbreda_OxA-21662", 37300, 800)
    {
color="Green";
      Outlier("General", 0.05);
    };
    R_Date("GrutadoCaldeirao_MAMS-41871", 36490, 390)
    {
color="Green";
      Outlier("General", 0.05);
    };
R_Date("FuentesdeSanCristobal_OxA-36200", 36200, 350)
    {
color="Green";
      Outlier("General", 0.05);
    };
   };
   Boundary("Final End Mousterian");
  };
```

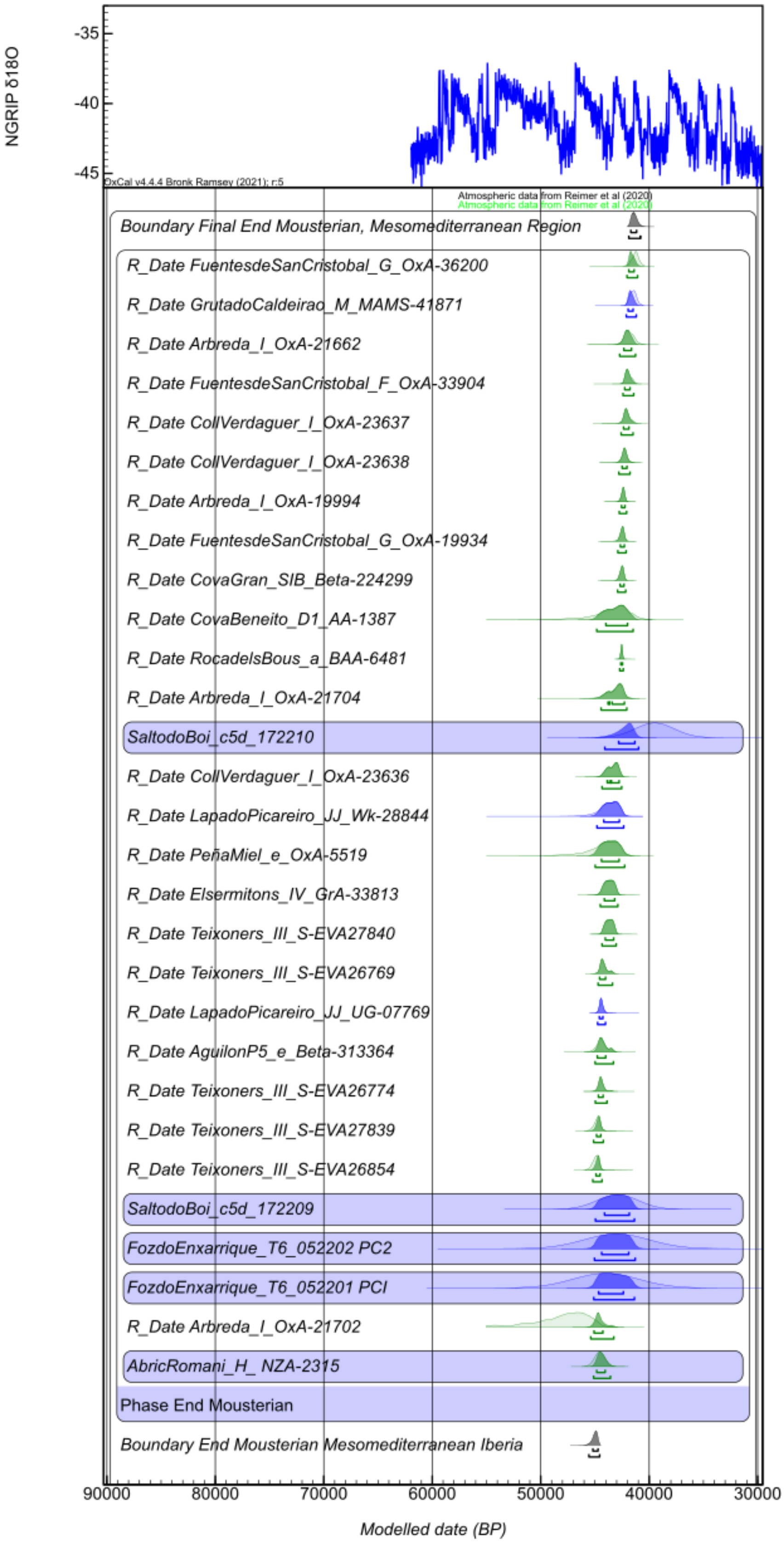

Figure 13 Plot of dated radiocarbon dates from Mousterian assemblages once the outlier dates (see the "summary" section for details) were removed. Sites located in the Mediterranean façade are shown in green while sites located in the Atlantic façade are shown in blue.

Name	Unmodelled (BP)						Modelled (BP)						Indices					
Amodel 100.7																		
Aoverall 100.2"																		
	from	to	%	from	to	%	from	to	%	from	to	%	Acomb	A	L	P	C	
Boundary Final End Mousterian	Mesomediterranean Region							41690	41165	68.268949	41880	40808	95.449974					95.7
R_Date FuentesdeSanCristobal_G_OxA-36200	41560	40950	68.268949	41874	40679	95.449974	41879	41390	68.268949	42046	41071	95.449974		72.9		93.7	99.4	
R_Date GrutadoCaldeirao_M_MAMS-41871	41796	41199	68.268949	42044	40911	95.449974	41941	41485	68.268949	42110	41190	95.449974		97.8		95.1	99.6	
R_Date Arbreda_I_OxA-21662	42319	41442	68.268949	42730	40912	95.449974	42337	41638	68.268949	42722	41261	95.449974		109.5		95.5	99.7	
R_Date FuentesdeSanCristobal_F_OxA-33904	42262	41717	68.268949	42401	41331	95.449974	42254	41771	68.268949	42414	41428	95.449974		106.2		95.6	99.7	
R_Date CollVerdaguer_I_OxA-23637	42375	41851	68.268949	42569	41404	95.449974	42363	41873	68.268949	42585	41489	95.449974		104.3		95.6	99.8	
R_Date CollVerdaguer_I_OxA-23638	42494	42049	68.268949	42801	41756	95.449974	42494	42050	68.268949	42800	41770	95.449974		101.3		95.6	99.8	
R_Date Arbreda_I_OxA-19994	42549	42238	68.268949	42750	42095	95.449974	42552	42236	68.268949	42761	42085	95.449974		100.7		95.7	99.8	
R_Date FuentesdeSanCristobal_G_OxA-19934	42650	42291	68.268949	42875	42139	95.449974	42655	42290	68.268949	42890	42126	95.449974		100.5		95.5	99.8	
R_Date CovaGran_SIB_Beta-224299	42681	42326	68.268949	42902	42176	95.449974	42683	42324	68.268949	42917	42167	95.449974		100.6		95.6	99.8	
R_Date CovaBeneito_D1_AA-1387	44523	41635	68.268949	48047	40382	95.449974	43991	41999	68.268949	44834	41482	95.449974		122.8		95.5	99.8	
R_Date RocadelsBous_a_BAA-6481	42618	42460	68.268949	42709	42392	95.449974	42619	42460	68.268949	42718	42386	95.449974		100.7		95.8	99.8	
R_Date Arbreda_I_OxA-21704	43795	42283	68.268949	44504	42037	95.449974	43799	42285	68.26895	44438	42069	95.449974		101.6		95.5	99.8	
N(39500	1800)	37646	41354	68.268949	35900	43100	95.449974	41318	42812	68.268949	40976	44090	95.449974		48		95	99.8
#¿NOMBRE?							1950	1951	68.268949	1950	1951	95.449974					100	
SaltodoBoi_c5d_172210	41354	37646	68.268949	43100	35900	95.449974	42812	41318	68.268949	44090	40976	95.449974					99.8	
R_Date CollVerdaguer_I_OxA-23636	43858	42771	68.268949	44336	42544	95.449974	43862	42773	68.268948	44344	42539	95.449974		100.6		95.5	99.8	
R_Date LapadoPicareiro_JJ_Wk-28844	44287	42690	68.268949	45515	42242	95.449974	44188	42750	68.268949	44812	42351	95.449974		107.8		95.5	99.8	

R_Date PeÃ±aMiel_e_OxA-5519	44783	42521	68.268949	47446	42029	95.449974	44400	42775	68.268949	44977	42259	95.449974		119.3		95.5	99.8	
R_Date Elsermitons_IV_GrA-33813	44129	43179	68.268949	44506	42895	95.449974	44134	43181	68.268949	44509	42892	95.449974		100.8		95.5	99.8	
R_Date Teixoners_III_S-EVA27840	44045	43278	68.268949	44336	43055	95.449974	44047	43275	68.268949	44344	43045	95.449974		100.5		95.5	99.8	
R_Date Teixoners_III_S-EVA26769	44570	44024	68.268949	44720	43385	95.449974	44571	44025	68.268949	44709	43385	95.449974		101.3		95.6	99.8	
R_Date LapadoPicareiro_JJ_UG-07769	44595	44271	68.268949	44765	44040	95.449974	44593	44273	68.268949	44755	44030	95.449974		101.7		95.7	99.8	
R_Date AguilonP5_e_Beta-313364	44895	43985	68.268949	45274	43274	95.449974	44785	44007	68.268949	44984	43295	95.449974		107.5		95.5	99.7	
R_Date Teixoners_III_S-EVA26774	44702	44240	68.268949	45050	43893	95.449974	44682	44247	68.268949	44964	43886	95.449974		104.2		95.6	99.8	
R_Date Teixoners_III_S-EVA27839	45005	44480	68.268949	45405	44277	95.449974	44853	44458	68.268949	45126	44227	95.449974		110.6		95.5	99.5	
R_Date Teixoners_III_S-EVA26854	45158	44598	68.268949	45538	44418	95.449974	44906	44529	68.268949	45200	44348	95.449974		105.8		95.1	99.3	
N(42900	1900)	40943	44857	68.268949	39100	46700	95.449974	41836	44116	68.268949	41361	44952	95.449974		123.9		95	99.9
#¿NOMBRE?							1950	1951	68.268949	1950	1951	95.449974					100	
SaltodoBoi_c5d_172209	44857	40943	68.268949	46700	39100	95.449974	44116	41836	68.268949	44952	41361	95.449974					99.9	
N(43000	3000)	39910	46090	68.268949	37000	49000	95.449974	41890	44380	68.268949	41290	45040	95.449974		133.2		95	99.9
#¿NOMBRE?							1950	1951	68.268949	1950	1951	95.449974					100	
FozdoEnxarrique_T6_052202 PC2	46090	39910	68.268949	49000	37000	95.449974	44380	41890	68.268949	45040	41290	95.449974					99.9	
N(44000	3000)	40910	47090	68.268949	38000	50000	95.449974	42380	44660	68.268949	41360	45110	95.449974		130.2		95	99.9
#¿NOMBRE?							1950	1951	68.268949	1950	1951	95.449974					100	
FozdoEnxarrique_T6_052201 PCI	47090	40910	68.268949	50000	38000	95.449974	44660	42380	68.268949	45110	41360	95.449974					99.9	
R_Date Arbreda_I_OxA-21702	49213	44955	68.268949	54674	44320	95.449974	45083	44272	68.268949	45385	43263	95.449974		45.6		93.3	99	
N(44560	480)	44065.6	45054.4	68.268949	43600	45520	95.449974	44060.8	44838.4	68.268949	43571.2	45126.4	95.449974		108		95	99.8
#¿NOMBRE?							1950	1951	68.268949	1950	1951	95.449974					100	
AbricRomani_H_ NZA-2315	45054	44066	68.268949	45520	43600	95.449974	44838	44061	68.268949	45126	43571	95.449974					99.8	

Phase End Mousterian																
Boundary End Mousterian Mesomediterranean Iberia							45190	44716	68.268949	45557	44568	95.449974			95.6	
Sequence																
N(0	2)	-2.06	2.06	68.268949	-4	4	95.449974									99.9
Outlier_Model SSimple							-2	3	68.268949	-2	3	95.449974			100	
U(0	4)	3.99E-17	4	68.268949	3.99E-17	4	95.449974	5.38E-17	2.472	68.268949	5.38E-17	3.652	95.449974	100		99.9
T(5)	-1.14	1.14	68.268949	-2.65	2.65	95.449974									99.9	
Outlier_Model General							-149	149	68.268949	-1576	1545	95.449973			100	
Curve IntCal20																

c. Excluding younger and oldest dates

```
Options()
 {
  Curve("IntCal20","intcal20.14c");
  BCAD=FALSE;
  SD1=TRUE;
  SD2=TRUE;
  ConvergenceData=TRUE;
  kIterations=300;
 };
Plot()
{
  Outlier_Model("General",T(5),U(0,4),"t");
Outlier_Model("SSimple",N(0,2),0,"s");
  Sequence()
  {
    Boundary("End Mousterian");
    Phase("End Mousterian")
{
      R_Date("Arbreda_OxA-21702", 44400, 1900)
      {
color="Green";
        Outlier("General", 0.05);
      };
  Age("FozdoEnxarrique_052201 PCI", N(44000, 3000))
      {
color="Green";
        Outlier("General", 0.05);
      };
Age("FozdoEnxarrique_052202 PC2", N(43000, 3000))
      {
color="Green";
        Outlier("General", 0.05);
      };
Age("SaltodoBoi_172209", N(42900, 1900))
      {
color="Green";
        Outlier("General", 0.05);
      };

R_Date("Teixoners_S-EVA26854", 42250, 359)
      {
color="Green";
        Outlier("General", 0.05);
      };
R_Date("Teixoners_S-EVA27839", 42020, 370)
      {
color="Green";
        Outlier("General", 0.05);
      };
R_Date("Teixoners_S-EVA26774", 41560, 337)
      {
color="Green";
        Outlier("General", 0.05);
      };
R_Date("Aguilon_Beta-313364", 41510, 550)
```

```
    {
color="Green";
      Outlier("General", 0.05);
    };
R_Date("LapadoPicareiro_UG-07769", 41480, 220)
    {
color="Green";
      Outlier("General", 0.05);
    };
R_Date("Teixoners_S-EVA26769", 41270, 327)
    {
color="Green";
      Outlier("General", 0.05);
    };
R_Date("Teixoners_S-EVA27840", 40610, 340)
    {
color="Green";
      Outlier("General", 0.05);
    };
R_Date("Elsermitons_GrA-33813", 40580, 550)
    {
color="Green";
      Outlier("General", 0.05);
    };
R_Date("PeñaMiel_OxA-5519", 40300, 1600)
    {
color="Green";
      Outlier("General", 0.05);
    };
R_Date("LapadoPicareiro_Wk-28844", 40100, 1200)
    {
color="Green";
      Outlier("General", 0.05);
    };
R_Date("CollVerdaguer_OxA-23636", 39950, 650)
    {
color="Green";
      Outlier("General", 0.05);
    };
Age("SaltodoBoi_172210", N(39500, 1800))
    {
color="Green";
      Outlier("General", 0.05);
    };
    R_Date("Arbreda_OxA-21704", 39200, 1000)
    {
color="Green";
      Outlier("General", 0.05);
    };
R_Date("RocadelsBous_BAA-6481", 38800, 120)
    {
color="Green";
      Outlier("General", 0.05);
    };
R_Date("CovaBeneito_AA-1387", 38800, 1900)
    {
color="Green";
      Outlier("General", 0.05);
    };
```

```
    R_Date("CovaGran_Beta-224299", 38640, 440)
    {
color="Green";
      Outlier("General", 0.05);
    };
R_Date("FuentesdeSanCristobal_OxA-19934", 38550, 450)
    {
color="Green";
      Outlier("General", 0.05);
    };
    R_Date("Arbreda_OxA-19994", 38350, 400)
    {
color="Green";
      Outlier("General", 0.05);
    };
    R_Date("CollVerdaguer_OxA-23638", 38000, 550)
    {
color="Green";
      Outlier("General", 0.05);
    };
    R_Date("CollVerdaguer_OxA-23637", 37600, 550)
    {
color="Green";
      Outlier("General", 0.05);
    };
    R_Date("FuentesdeSanCristobal_OxA-33904", 37330, 490)
    {
color="Green";
      Outlier("General", 0.05);
    };
    R_Date("Arbreda_OxA-21662", 37300, 800)
    {
color="Green";
      Outlier("General", 0.05);
    };
    R_Date("GrutadoCaldeirao_MAMS-41871", 36490, 390)
    {
color="Green";
      Outlier("General", 0.05);
    };
   };
   Boundary("Final End Mousterian");
  };
 };
};
```

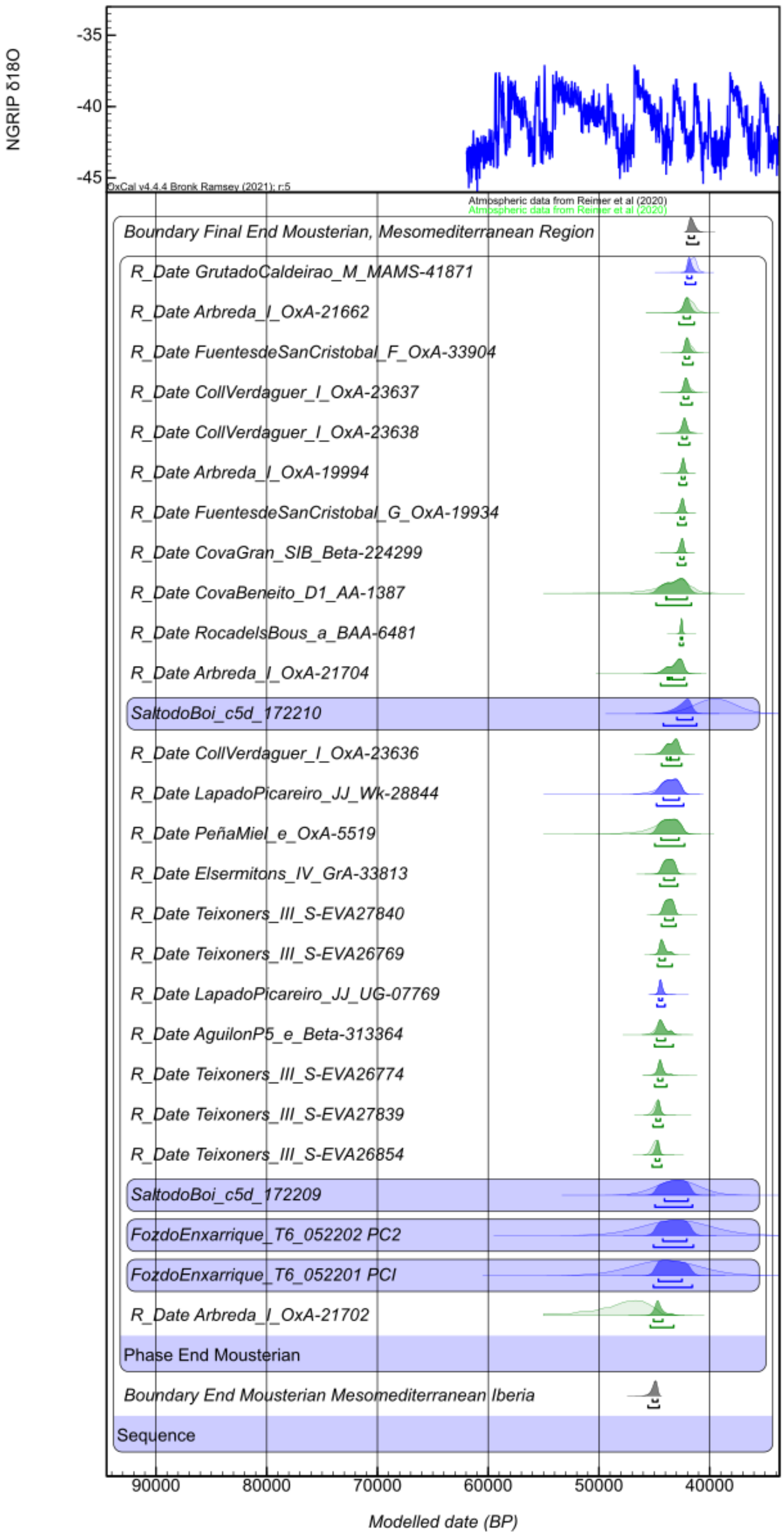

Figure 14. Plot of dated radiocarbon dates from Mousterian assemblages once the youngest and oldest dates were removed. Sites located in the Mediterranean façade are shown in green while sites located in the Atlantic façade are shown in blue.

Name	Unmodelled (BP)						Modelled (BP)						Indices					
Amodel 100.5																		
Aoverall 100.6"																		
	from	to	%	from	to	%	from	to	%	from	to	%	Acomb	A	L	P	C	
Boundary Final End Mousterian	Mesomediterranean Region							41898	41392	68.268949	42064	41010	95.449974					96.4
R_Date GrutadoCaldeirao_M_MAMS-41871	41796	41199	68.268949	42044	40911	95.449974	42038	41619	68.268949	42206	41270	95.449974		80.1		93.4	99.3	
R_Date Arbreda_I_OxA-21662	42319	41442	68.268949	42730	40912	95.449974	42370	41758	68.268949	42770	41389	95.449974		110.5		95.5	99.7	
R_Date FuentesdeSanCristobal_F_OxA-33904	42262	41717	68.268949	42401	41331	95.449974	42255	41835	68.268949	42434	41515	95.449974		110.6		95.6	99.8	
R_Date CollVerdaguer_I_OxA-23637	42375	41851	68.268949	42569	41404	95.449974	42358	41912	68.268949	42600	41586	95.449974		108.5		95.6	99.8	
R_Date CollVerdaguer_I_OxA-23638	42494	42049	68.268949	42801	41756	95.449974	42490	42056	68.268949	42789	41817	95.449974		102.9		95.6	99.8	
R_Date Arbreda_I_OxA-19994	42549	42238	68.268949	42750	42095	95.449974	42552	42236	68.268949	42765	42085	95.449974		100.6		95.6	99.8	
R_Date FuentesdeSanCristobal_G_OxA-19934	42650	42291	68.268949	42875	42139	95.449974	42651	42290	68.268949	42890	42126	95.449974		100.6		95.5	99.8	
R_Date CovaGran_SIB_Beta-224299	42681	42326	68.268949	42902	42176	95.449974	42686	42326	68.268949	42915	42165	95.449974		100.6		95.5	99.8	
R_Date CovaBeneito_D1_AA-1387	44523	41635	68.268949	48047	40382	95.449974	43953	42033	68.26895	44834	41647	95.449974		124.8		95.5	99.8	
R_Date RocadelsBous_a_BAA-6481	42618	42460	68.268949	42709	42392	95.449974	42620	42460	68.268949	42719	42385	95.449974		100.7		95.7	99.9	
R_Date Arbreda_I_OxA-21704	43795	42283	68.268949	44504	42037	95.449974	43801	42283	68.26895	44425	42080	95.449974		101.9		95.5	99.7	
N(39500	1800)	37646	41354	68.268949	35900	43100	95.449974	41534	42956	68.268949	41174	44180	95.449974		42		95	99.9
#¿NOMBRE?							1950	1951	68.268949	1950	1951	95.449974					100	
SaltodoBoi_c5d_172210	41354	37646	68.268949	43100	35900	95.449974	42956	41534	68.268949	44180	41174	95.449974					99.9	
R_Date CollVerdaguer_I_OxA-23636	43858	42771	68.268949	44336	42544	95.449974	43869	42771	68.268948	44337	42536	95.449974		100.7		95.5	99.8	
R_Date LapadoPicareiro_JJ_Wk-28844	44287	42690	68.268949	45515	42242	95.449974	44189	42753	68.268949	44794	42350	95.449974		108.1		95.5	99.8	
R_Date PeÃ±aMiel_e_OxA-5519	44783	42521	68.268949	47446	42029	95.449974	44392	42777	68.268949	44951	42265	95.449974		119.7		95.5	99.8	

R_Date Elsermitons_IV_GrA-33813	44129	43179	68.268949	44506	42895	95.449974	44129	43182	68.268949	44505	42894	95.449974		100.9	95.5	99.8	
R_Date Teixoners_III_S-EVA27840	44045	43278	68.268949	44336	43055	95.449974	44047	43276	68.268949	44345	43045	95.449974		100.5	95.5	99.8	
R_Date Teixoners_III_S-EVA26769	44570	44024	68.268949	44720	43385	95.449974	44568	44023	68.268949	44708	43384	95.449974		101.5	95.6	99.8	
R_Date LapadoPicareiro_JJ_UG-07769	44595	44271	68.268949	44765	44040	95.449974	44591	44273	68.268949	44755	44032	95.449974		101.7	95.7	99.8	
R_Date AguilonP5_e_Beta-313364	44895	43985	68.268949	45274	43274	95.449974	44781	44007	68.268949	44971	43296	95.449974		107.7	95.6	99.8	
R_Date Teixoners_III_S-EVA26774	44702	44240	68.268949	45050	43893	95.449974	44680	44247	68.268949	44960	43875	95.449974		104.5	95.6	99.8	
R_Date Teixoners_III_S-EVA27839	45005	44480	68.268949	45405	44277	95.449974	44841	44452	68.268949	45115	44217	95.449974		110.3	95.5	99.6	
R_Date Teixoners_III_S-EVA26854	45158	44598	68.268949	45538	44418	95.449974	44897	44521	68.268949	45185	44335	95.449974		104.5	95.1	99.5	
N(42900	1900)	40943	44857	68.268949	39100	46700	95.449974	41950	44078	68.268949	41551	44933	95.449974	124.9		95	100
#¿NOMBRE?							1950	1951	68.268949	1950	1951	95.449974				100	
SaltodoBoi_c5d_172209	44857	40943	68.268949	46700	39100	95.449974	44078	41950	68.268949	44933	41551	95.449974				100	
N(43000	3000)	39910	46090	68.268949	37000	49000	95.449974	42070	44230	68.268949	41470	45070	95.449974	133.8		95	99.9
#¿NOMBRE?							1950	1951	68.268949	1950	1951	95.449974				100	
FozdoEnxarrique_T6_052202 PC2	46090	39910	68.268949	49000	37000	95.449974	44230	42070	68.268949	45070	41470	95.449974				99.9	
N(44000	3000)	40910	47090	68.268949	38000	50000	95.449974	42500	44630	68.268949	41570	45080	95.449974	131.6		95	99.9
#¿NOMBRE?							1950	1951	68.268949	1950	1951	95.449974				100	
FozdoEnxarrique_T6_052201 PCI	47090	40910	68.268949	50000	38000	95.449974	44630	42500	68.268949	45080	41570	95.449974				99.9	
R_Date Arbreda_I_OxA-21702	49213	44955	68.268949	54674	44320	95.449974	45065	44245	68.268949	45356	43249	95.449974		44.4	93.2	99.4	
Phase End Mousterian																	
Boundary End Mousterian Mesomediterranean Iberia							45157	44695	68.268949	45556	44553	95.449974				95.9	
Sequence																	
N(0	2)	-2.06	2.06	68.268949	-4	4	95.449974										99.9

Outlier_Model SSimple							-2	3	68.268949	-2	3	95.449974			100	
U(0	4)	3.99E-17	4	68.268949	3.99E-17	4	95.449974	5.38E-17	2.516	68.268949	5.38E-17	3.652	95.449974	100		99.9
T(5)	-1.14	1.14	68.268949	-2.65	2.65	95.449974									99.9	
Outlier_Model General							-149	151	68.268949	-1564	1495	95.449974			100	

Curve IntCal20

2.3.2. Aurignacian

a. All dates

```
Options()
 {
  Curve("IntCal20","intcal20.14c");
  BCAD=FALSE;
  SD1=TRUE;
  SD2=TRUE;
  ConvergenceData=TRUE;
  kIterations=300;
 };
Plot()
{
  Outlier_Model("General",T(5),U(0,4),"t");
Outlier_Model("SSimple",N(0,2),0,"s");
  Sequence()
  {
    Boundary("Start Aurignacian");
    Phase("Aurignacian")
{
     R_Date("CovaBeneito_Gif-8307", 38200, 1600)
     {
color="Green";
       Outlier("General", 0.05);
     };
            R_Date("Arbreda_AA-3379", 37700, 1000)
     {
color="Green";
       Outlier("General", 0.05);
     };
R_Date("AbricRomani_AA-6608", 36740, 640)
     {
color="Green";
       Outlier("General", 0.05);
     };
     R_Date("LapadoPicareiro_MAMS-42282", 36670, 220)
     {
color="Green";
       Outlier("General", 0.05);
     };
     R_Date("AbricRomani_NzA-2311",36590, 640)
     {
color="Green";
       Outlier("General", 0.05);
     };
R_Date("LapadoPicareiro_MAMS-42278", 36390, 210)
     {
color="Green";
       Outlier("General", 0.05);
     };
R_Date("Arbreda_OxA-21784", 36000, 700)
     {
color="Green";
       Outlier("General", 0.05);
```

```
    };
    R_Date("AbricRomani_OxA-11967", 35900, 600)
    {
color="Green";
      Outlier("General", 0.05);
    };
R_Date("Arbreda_OxA-21664", 35900, 650)
    {
color="Green";
      Outlier("General", 0.05);
    };
    R_Date("Arbreda_SANU-29019", 35900, 860)
    {
color="Green";
      Outlier("General", 0.05);
    };
    R_Date("Arbreda_OxA-21665", 35850, 700)
    {
color="Green";
      Outlier("General", 0.05);
    };
    R_Date("Arbreda_SANU-29016", 35700, 830)
    {
color="Green";
      Outlier("General", 0.05);
    };
R_Date("AbricRomani_AA-8037A", 35400, 810)
    {
color="Green";
      Outlier("General", 0.05);
    };
R_Date("AbricRomani_USGS-2840", 35000, 500)
    {
color="Green";
      Outlier("General", 0.05);
    };
    R_Date("RieradelsCanyars_OxA-2416-44", 34980, 350)
    {
color="Green";
      Outlier("General", 0.05);
    };
    R_Date("RieradelsCanyars_OxA-24057", 34900, 340)
    {
color="Green";
      Outlier("General", 0.05);
    };
R_Date("RieradelsCanyars_OxA-23644", 34810, 360)
    {
color="Green";
      Outlier("General", 0.05);
    };
R_Date("RieradelsCanyars_OxA-23643", 34540, 330)
    {
color="Green";
      Outlier("General", 0.05);
    };
R_Date("CovaGran_AA-68834-ABOX", 34179, 247)
    {
color="Green";
```

```
        Outlier("General", 0.05);
      };
R_Date("CovaGran_AA68834c", 34174, 247)
      {
color="Green";
        Outlier("General", 0.05);
      };
R_Date("LapadoPicareiro_MAMS-42277", 33910, 160)
      {
color="Green";
        Outlier("General", 0.05);
      };
R_Date("CovaBeneito_AA-1388", 33900, 1100)
      {
color="Green";
        Outlier("General", 0.05);
      };
R_Date("LapadoPicareiro_MAMS-44445", 33880, 160)
      {
color="Green";
        Outlier("General", 0.05);
      };
      R_Date("Arbreda_OxA-21674", 33800, 550)
      {
color="Green";
        Outlier("General", 0.05);
      };
      R_Date("RieradelsCanyars_Beta-273965", 33800, 350)
      {
color="Green";
        Outlier("General", 0.05);
      };
R_Date("LapadoPicareiro_MAMS-42281", 33790, 190)
      {
color="Green";
        Outlier("General", 0.05);
      };
      R_Date("Mollet_OxA-3728", 33780, 730)
      {
color="Green";
        Outlier("General", 0.05);
      };
R_Date("CovaGran_AA68834b", 33068, 261)
      {
color="Green";
        Outlier("General", 0.05);
      };
      R_Date("Arbreda_OxA-21666", 32750, 450)
      {
color="Green";
        Outlier("General", 0.05);
      };
R_Date("CovaGran_Beta-207578", 32630, 450)
      {
color="Green";
        Outlier("General", 0.05);
      };
R_Date("CovaGran_AA68834a", 32368, 241)
      {
```

```
color="Green";
       Outlier("General", 0.05);
     };
     R_Date("Arbreda_OxA-21667", 32250, 450)
     {
color="Green";
       Outlier("General", 0.05);
     };
     R_Date("Arbreda_OxA-21783", 32100, 450)
     {
color="Green";
       Outlier("General", 0.05);
     };
     R_Date("S Arbreda_ANU-29018", 32100, 540)
     {
color="Green";
       Outlier("General", 0.05);
     };
     R_Date("Arbreda_SANU-29014", 31900, 530)
     {
color="Green";
       Outlier("General", 0.05);
     };
    };
    Boundary("End Aurignacian");
   };
  };
 };
```

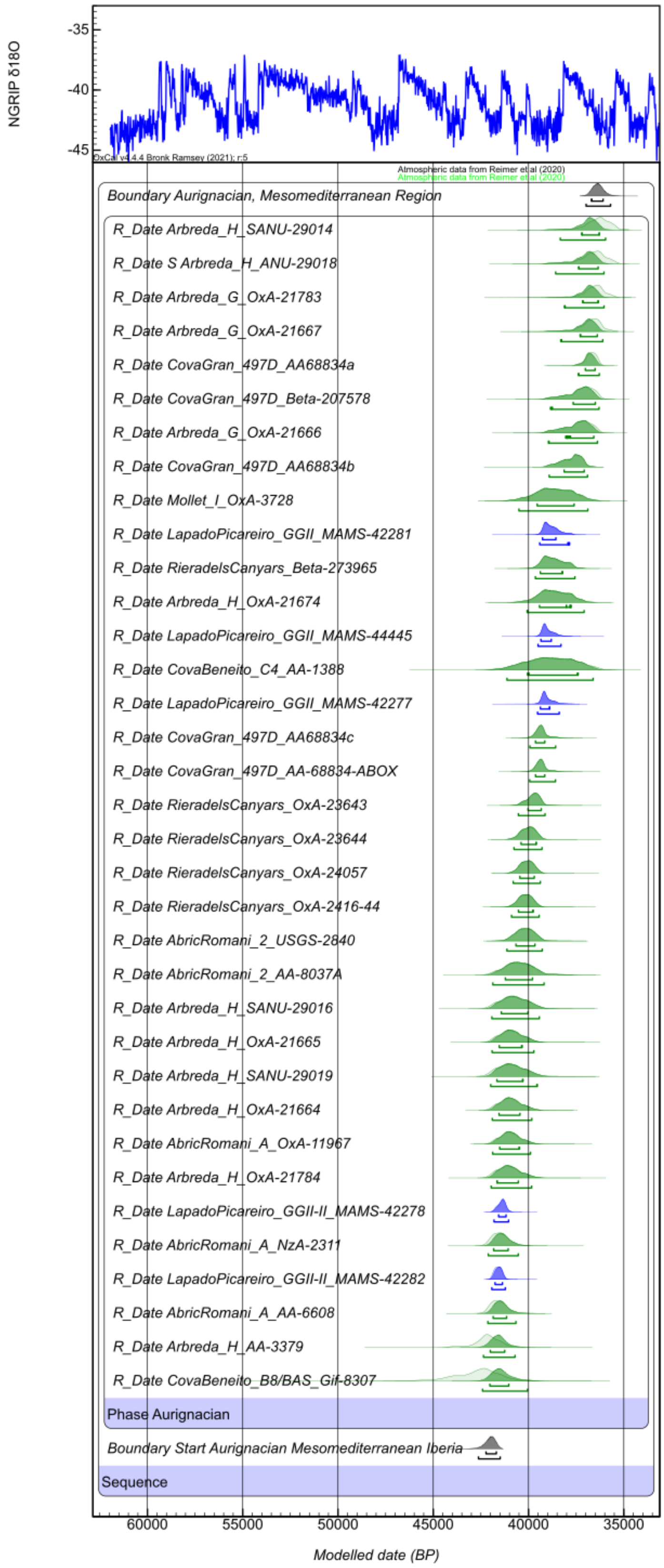

Figure 15. Plot of dated radiocarbon dates from Mousterian assemblages in the Mesomediterranean region. Sites located in the Mediterranean façade are shown in green while sites located in the Atlantic façade are shown in blue.

Name	Unmodelled (BP)						Modelled (BP)						Indices					
Amodel 100																		
Aoverall 96.5"																		
	from	to	%	from	to	%	from	to	%	from	to	%	Acomb	A	L	P	C	
Boundary Aurignacian	Mesomediterranean Region							36696	36089	68.268949	36977	35696	95.449974					95.6
R_Date Arbreda_H_SANU-29014	36852	35675	68.268949	37718	35165	95.449974	37205	36279	68.268949	38332	35957	95.449974		82.8		94.9	98.6	
R_Date S Arbreda_H_ANU-29018	37125	35873	68.268949	38226	35326	95.449974	37366	36346	68.268949	38576	36040	95.449974		95		95.2	98.8	
R_Date Arbreda_G_OxA-21783	37000	36035	68.268949	37610	35430	95.449974	37157	36335	68.268949	38116	36037	95.449973		95.3		95.1	98.9	
R_Date Arbreda_G_OxA-21667	37115	36138	68.268949	38075	35562	95.449974	37292	36391	68.26895	38308	36104	95.449974		98.8		95.3	98.9	
R_Date CovaGran_497D_AA68834a	36939	36401	68.268949	37262	36192	95.449974	37016	36505	68.268949	37378	36285	95.449973		98.9		95.5	99.3	
R_Date CovaGran_497D_Beta-207578	37596	36372	68.268949	38794	36156	95.449974	37655	36494	68.268949	38848	36299	95.449973		102.2		95.4	99.2	
R_Date Arbreda_G_OxA-21666	37814	36425	68.268949	38911	36260	95.449974	38051	36575	68.268948	38955	36382	95.449974		102.5		95.4	99.2	
R_Date CovaGran_497D_AA68834b	38130	37083	68.268949	38906	36910	95.449974	38131	37077	68.268949	38916	36904	95.449974		100.4		95.3	99.2	
R_Date Mollet_I_OxA-3728	39551	37604	68.268949	40525	36859	95.449974	39547	37607	68.268949	40503	36888	95.449974		100.8		95.3	99.4	
R_Date LapadoPicareiro_GGII_MAMS-42281	39264	38566	68.268949	39410	37897	95.449974	39265	38565	68.268949	39413	37849	95.449974		100.4		95.2	99.4	
R_Date RieradelsCanyars_Beta-273965	39375	38224	68.268949	39630	37576	95.449974	39381	38210	68.268949	39641	37567	95.449974		100.2		95.3	99.3	
R_Date Arbreda_H_OxA-21674	39415	37815	68.268949	40051	37090	95.449974	39425	37763	68.268949	40074	37082	95.449973		100.4		95.3	99.3	
R_Date LapadoPicareiro_GGII_MAMS-44445	39347	38828	68.268949	39484	38333	95.449974	39350	38806	68.268949	39501	38299	95.449974		100.2		95.3	99.4	
R_Date CovaBeneito_C4_AA-1388	40055	37363	68.268949	41207	36376	95.449974	40049	37398	68.268949	41131	36609	95.449974		102.5		95.3	99.3	
R_Date LapadoPicareiro_GGII_MAMS-42277	39375	38897	68.268949	39508	38411	95.449974	39377	38889	68.26895	39525	38388	95.449974		100.4		95.3	99.5	
R_Date CovaGran_497D_AA68834c	39625	39148	68.268949	39920	38600	95.449974	39634	39147	68.268949	39927	38579	95.449974		100.3		95.3	99.5	
R_Date CovaGran_497D_AA-68834-ABOX	39628	39152	68.268949	39926	38615	95.449974	39632	39149	68.268949	39939	38587	95.449974		100.1		95.3	99.5	

R_Date RieradelsCanyars_OxA-23643	40020	39335	68.268949	40512	39150	95.449974	40027	39332	68.268949	40532	39133	95.449974	100.2	95.3	99.4	
R_Date RieradelsCanyars_OxA-23644	40389	39609	68.268949	40740	39304	95.449974	40392	39594	68.268949	40762	39292	95.449974	100.3	95.3	99.3	
R_Date RieradelsCanyars_OxA-24057	40451	39708	68.268949	40785	39393	95.449974	40456	39699	68.268949	40805	39380	95.449974	100.3	95.3	99.5	
R_Date RieradelsCanyars_OxA-2416-44	40523	39769	68.268949	40879	39454	95.449974	40529	39759	68.268949	40892	39444	95.449974	100.3	95.2	99.3	
R_Date AbricRomani_2_USGS-2840	40655	39666	68.268949	41116	39287	95.449974	40659	39663	68.268949	41133	39279	95.449974	100.3	95.3	99.3	
R_Date AbricRomani_2_AA-8037A	41222	39770	68.268949	41985	39190	95.449974	41214	39791	68.268949	41874	39184	95.449974	101.9	95.3	99.3	
R_Date Arbreda_H_SANU-29016	41464	40003	68.268949	42070	39429	95.449974	41431	40027	68.268949	41923	39434	95.449974	102.9	95.3	99.3	
R_Date Arbreda_H_OxA-21665	41599	40345	68.268949	42020	39727	95.449974	41537	40337	68.268949	41913	39720	95.449974	102.8	95.4	99.3	
R_Date Arbreda_H_SANU-29019	41770	40315	68.268949	42173	39558	95.449974	41660	40289	68.268949	41984	39543	95.449973	103.7	95.3	99.3	
R_Date Arbreda_H_OxA-21664	41599	40442	68.268949	42002	39834	95.449974	41549	40452	68.268949	41900	39829	95.449974	102.8	95.4	99.4	
R_Date AbricRomani_A_OxA-11967	41541	40469	68.268949	41961	39900	95.449974	41513	40474	68.26895	41884	39897	95.449974	102.4	95.4	99.3	
R_Date Arbreda_H_OxA-21784	41722	40535	68.268949	42087	39855	95.449974	41656	40535	68.268949	41960	39839	95.449974	103.3	95.4	99.3	
R_Date LapadoPicareiro_GGII-II_MAMS-42278	41588	41181	68.268949	41826	41055	95.449974	41569	41180	68.268949	41811	41045	95.449974	102.5	95.6	99.6	
R_Date AbricRomani_A_NzA-2311	41976	41126	68.268949	42273	40597	95.449974	41830	41069	68.268949	42112	40537	95.449974	103.6	95.4	99.3	
R_Date LapadoPicareiro_GGII-II_MAMS-42282	41808	41401	68.268949	41965	41240	95.449974	41757	41370	68.268949	41928	41215	95.449974	102.4	95.6	99.4	
R_Date AbricRomani_A_AA-6608	42032	41226	68.268949	42319	40726	95.449974	41856	41151	68.268949	42134	40661	95.449974	102.9	95.4	99.3	
R_Date Arbreda_H_AA-3379	42606	41495	68.268949	43783	40767	95.449973	42006	41251	68.268949	42364	40707	95.449974	86.1	94.9	99.1	
R_Date CovaBeneito_B8/BAS_Gif-8307	43822	41324	68.26895	45715	40323	95.449974	42029	41036	68.268949	42413	40053	95.449974	83.8	94.7	98.9	
Phase Aurignacian																
Boundary Start Aurignacian Mesomediterranean Iberia							42231	41690	68.268949	42627	41488	95.449974			95.3	
Sequence																
N(0	2)	-2.06	2.06	68.268949	-4	4	95.449974									100

Outlier_Model SSimple														100	
U(0 4)		3.99E-17	4	68.268949	3.99E-17	4	95.449974	5.38E-17	2.36	68.268949	5.38E-17	3.536	95.449974	100		99.8
T(5)	-1.14	1.14	68.268949	-2.65	2.65	95.449974								99.9	
Outlier_Model General						-132	132	68.268949	-1479	1355	95.449974			100	

Curve IntCal20

b. **Without Oldest and Youngest dates**

```
Options()
 {
  Curve("IntCal20","intcal20.14c");
  BCAD=FALSE;
  SD1=TRUE;
  SD2=TRUE;
  ConvergenceData=TRUE;
  kIterations=300;
 };
Plot()
{
  Outlier_Model("General",T(5),U(0,4),"t");
Outlier_Model("SSimple",N(0,2),0,"s");
  Sequence()
  {
    Boundary("Start Aurignacian");
    Phase("Aurignacian")
{
              R_Date("Arbreda_AA-3379", 37700, 1000)
     {
color="Green";
       Outlier("General", 0.05);
     };
R_Date("AbricRomani_AA-6608", 36740, 640)
     {
color="Green";
       Outlier("General", 0.05);
     };
     R_Date("LapadoPicareiro-II_MAMS-42282", 36670, 220)
     {
color="Green";
       Outlier("General", 0.05);
     };
     R_Date("AbricRomani_NzA-2311",36590, 640)
     {
color="Green";
       Outlier("General", 0.05);
     };
R_Date("LapadoPicareiro_MAMS-42278", 36390, 210)
     {
color="Green";
       Outlier("General", 0.05);
     };
R_Date("Arbreda_H_OxA-21784", 36000, 700)
     {
color="Green";
       Outlier("General", 0.05);
     };
     R_Date("AbricRomani_OxA-11967", 35900, 600)
     {
color="Green";
       Outlier("General", 0.05);
     };
R_Date("Arbreda_OxA-21664", 35900, 650)
     {
color="Green";
```

```
      Outlier("General", 0.05);
     };
     R_Date("Arbreda_SANU-29019", 35900, 860)
     {
color="Green";
      Outlier("General", 0.05);
     };
     R_Date("Arbreda_OxA-21665", 35850, 700)
     {
color="Green";
      Outlier("General", 0.05);
     };
     R_Date("Arbreda_SANU-29016", 35700, 830)
     {
color="Green";
      Outlier("General", 0.05);
     };
R_Date("AbricRomani_AA-8037A", 35400, 810)
     {
color="Green";
      Outlier("General", 0.05);
     };
R_Date("AbricRomani_USGS-2840", 35000, 500)
     {
color="Green";
      Outlier("General", 0.05);
     };
     R_Date("RieradelsCanyars_OxA-2416-44", 34980, 350)
     {
color="Green";
      Outlier("General", 0.05);
     };
     R_Date("RieradelsCanyars_OxA-24057", 34900, 340)
     {
color="Green";
      Outlier("General", 0.05);
     };
R_Date("RieradelsCanyars_OxA-23644", 34810, 360)
     {
color="Green";
      Outlier("General", 0.05);
     };
R_Date("RieradelsCanyars_OxA-23643", 34540, 330)
     {
color="Green";
      Outlier("General", 0.05);
     };
R_Date("CovaGran_AA-68834-ABOX", 34179, 247)
     {
color="Green";
      Outlier("General", 0.05);
     };
R_Date("CovaGran_AA68834c", 34174, 247)
     {
color="Green";
      Outlier("General", 0.05);
     };
R_Date("LapadoPicareiro_MAMS-42277", 33910, 160)
     {
```

```
color="Green";
       Outlier("General", 0.05);
     };
R_Date("CovaBeneito_AA-1388", 33900, 1100)
     {
color="Green";
       Outlier("General", 0.05);
     };
R_Date("LapadoPicareiro_MAMS-44445", 33880, 160)
     {
color="Green";
       Outlier("General", 0.05);
     };
     R_Date("Arbreda_OxA-21674", 33800, 550)
     {
color="Green";
       Outlier("General", 0.05);
     };
     R_Date("RieradelsCanyars_Beta-273965", 33800, 350)
     {
color="Green";
       Outlier("General", 0.05);
     };
R_Date("LapadoPicareiro_MAMS-42281", 33790, 190)
     {
color="Green";
       Outlier("General", 0.05);
     };
     R_Date("Mollet_OxA-3728", 33780, 730)
     {
color="Green";
       Outlier("General", 0.05);
     };
R_Date("CovaGran_AA68834b", 33068, 261)
     {
color="Green";
       Outlier("General", 0.05);
     };
     R_Date("Arbreda_OxA-21666", 32750, 450)
     {
color="Green";
       Outlier("General", 0.05);
     };
R_Date("CovaGran_497D_Beta-207578", 32630, 450)
     {
color="Green";
       Outlier("General", 0.05);
     };
R_Date("CovaGran_AA68834a", 32368, 241)
     {
color="Green";
       Outlier("General", 0.05);
     };
     R_Date("Arbreda_OxA-21667", 32250, 450)
     {
color="Green";
       Outlier("General", 0.05);
     };
     R_Date("Arbreda_OxA-21783", 32100, 450)
```

```
      {
color="Green";
        Outlier("General", 0.05);
      };
      R_Date("S Arbreda_ANU-29018", 32100, 540)
      {
color="Green";
        Outlier("General", 0.05);
      };
    };
    Boundary("End Aurignacian ");
   };
  };
 };
```

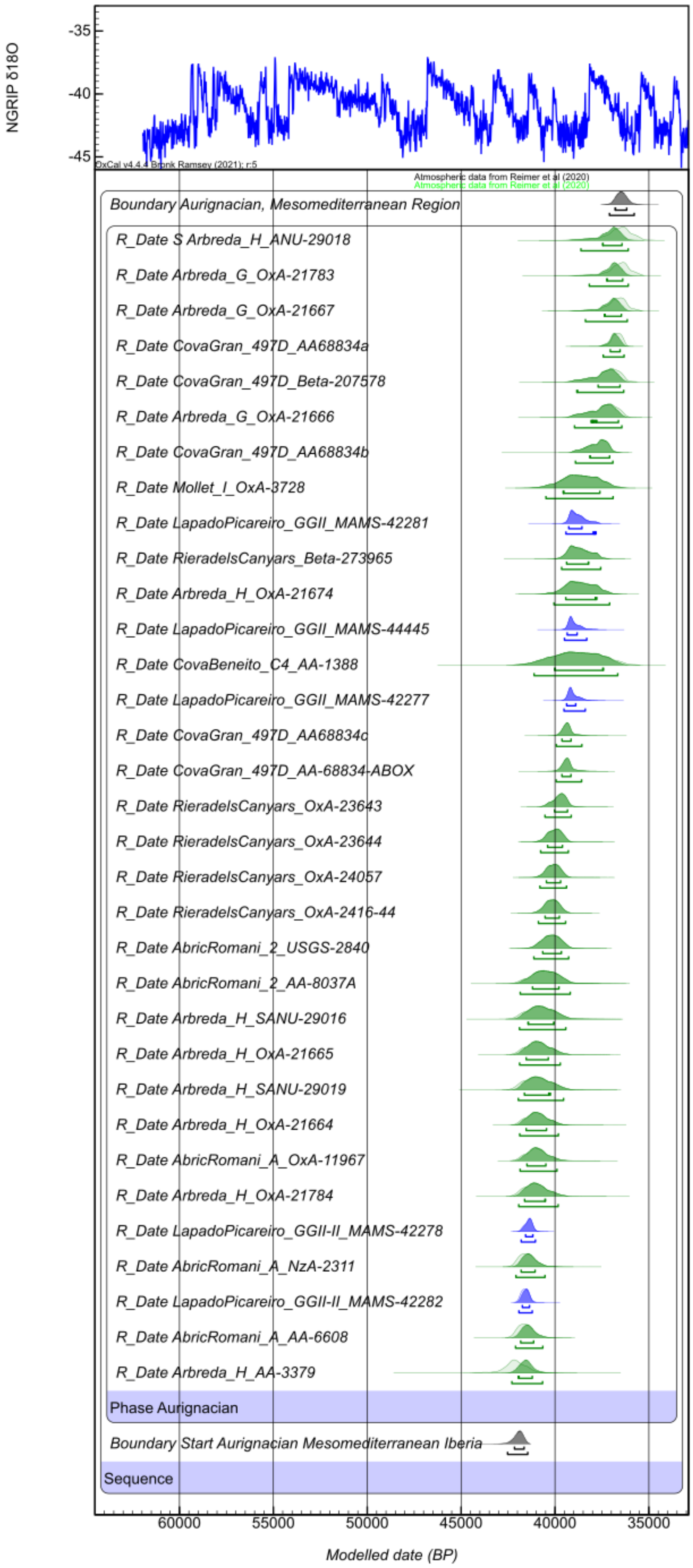

Figure 16. Plot of dated radiocarbon dates from Mousterian assemblages in the Mesomediterranean region after excluding the oldest and youngest dates. Sites located in the Mediterranean façade are shown in green while sites located in the Atlantic façade are shown in blue.

Name	Unmodelled (BP)						Modelled (BP)						Indices					
Amodel 102.5																		
Aoverall 98.7"																		
	from	to	%	from	to	%	from	to	%	from	to	%	Acomb	A	L	P	C	
Boundary Aurignacian			Mesomediterranean Region					36793	36180	68.26895	37097	35781	95.449974					97.1
R_Date S Arbreda_H_ANU-29018	37125	35873	68.268949	38226	35326	95.449974	37459	36431	68.268949	38618	36100	95.449974		89.5		95.1	99	
R_Date Arbreda_G_OxA-21783	37000	36035	68.268949	37610	35430	95.449974	37238	36394	68.268949	38173	36098	95.449974		88.9		95	99	
R_Date Arbreda_G_OxA-21667	37115	36138	68.268949	38075	35562	95.449974	37370	36464	68.268949	38378	36150	95.449974		94.2		95.2	99.2	
R_Date CovaGran_497D_AA68834a	36939	36401	68.268949	37262	36192	95.449974	37055	36534	68.268949	37432	36319	95.449974		94.9		95.1	99.2	
R_Date CovaGran_497D_Beta-207578	37596	36372	68.268949	38794	36156	95.449974	37702	36536	68.268949	38837	36341	95.449974		101.3		95.3	99.2	
R_Date Arbreda_G_OxA-21666	37814	36425	68.268949	38911	36260	95.449974	38077	36619	68.268949	38958	36440	95.449974		102.4		95.4	99.1	
R_Date CovaGran_497D_AA68834b	38130	37083	68.268949	38906	36910	95.449974	38137	37090	68.268949	38909	36916	95.449974		100.9		95.3	99.4	
R_Date Mollet_I_OxA-3728	39551	37604	68.268949	40525	36859	95.449974	39564	37610	68.268949	40481	36906	95.449974		101.1		95.3	99.3	
R_Date LapadoPicareiro_GGII_MAMS-42281	39264	38566	68.268949	39410	37897	95.449974	39265	38563	68.268949	39417	37816	95.449973		100.3		95.3	99.4	
R_Date RieradelsCanyars_Beta-273965	39375	38224	68.268949	39630	37576	95.449974	39379	38213	68.268949	39646	37565	95.449974		100.2		95.2	99.3	
R_Date Arbreda_H_OxA-21674	39415	37815	68.268949	40051	37090	95.449974	39426	37774	68.268949	40054	37091	95.449974		100.5		95.3	99.4	
R_Date LapadoPicareiro_GGII_MAMS-44445	39347	38828	68.268949	39484	38333	95.449974	39350	38822	68.268949	39495	38309	95.449974		100.4		95.4	99.4	
R_Date CovaBeneito_C4_AA-1388	40055	37363	68.268949	41207	36376	95.449974	40023	37419	68.268949	41114	36659	95.449974		102.9		95.3	99.3	
R_Date LapadoPicareiro_GGII_MAMS-42277	39375	38897	68.268949	39508	38411	95.449974	39379	38895	68.268949	39522	38387	95.449974		100.2		95.3	99.6	
R_Date CovaGran_497D_AA68834c	39625	39148	68.268949	39920	38600	95.449974	39627	39144	68.268949	39928	38569	95.449974		100.3		95.2	99.5	
R_Date CovaGran_497D_AA-	39628	39152	68.268949	39926	38615	95.449	39630	39148	68.268	39937	38584	95.449		100		95.	99.	

68834-ABOX						974			949			974	.3	3	5	
R_Date RieradelsCanyars_OxA-23643	40020	39335	68.268949	40512	39150	95.449<br>974	40026	39333	68.268<br>949	40531	39136	95.449<br>974	100<br>.3	95.<br>3	99.<br>6	
R_Date RieradelsCanyars_OxA-23644	40389	39609	68.268949	40740	39304	95.449<br>974	40394	39605	68.268<br>949	40762	39289	95.449<br>974	100<br>.3	95.<br>3	99.<br>4	
R_Date RieradelsCanyars_OxA-24057	40451	39708	68.268949	40785	39393	95.449<br>974	40457	39702	68.268<br>949	40803	39385	95.449<br>974	100<br>.3	95.<br>3	99.<br>4	
R_Date RieradelsCanyars_OxA-2416-44	40523	39769	68.268949	40879	39454	95.449<br>974	40526	39766	68.268<br>949	40884	39434	95.449<br>974	100<br>.4	95.<br>4	99.<br>4	
R_Date AbricRomani_2_USGS-2840	40655	39666	68.268949	41116	39287	95.449<br>974	40653	39660	68.268<br>949	41125	39270	95.449<br>974	100<br>.4	95.<br>3	99.<br>4	
R_Date AbricRomani_2_AA-8037A	41222	39770	68.268949	41985	39190	95.449<br>974	41195	39791	68.268<br>949	41851	39189	95.449<br>974	102<br>.3	95.<br>3	99.<br>3	
R_Date Arbreda_H_SANU-29016	41464	40003	68.268949	42070	39429	95.449<br>974	41437	40047	68.268<br>949	41892	39426	95.449<br>974	103<br>.5	95.<br>3	99.<br>3	
R_Date Arbreda_H_OxA-21665	41599	40345	68.268949	42020	39727	95.449<br>974	41534	40354	68.268<br>949	41885	39719	95.449<br>974	103<br>.4	95.<br>4	99.<br>4	
R_Date Arbreda_H_SANU-29019	41770	40315	68.268949	42173	39558	95.449<br>974	41628	40244	68.268<br>949	41954	39544	95.449<br>974	104<br>.2	95.<br>3	99.<br>3	
R_Date Arbreda_H_OxA-21664	41599	40442	68.268949	42002	39834	95.449<br>974	41536	40452	68.268<br>949	41879	39820	95.449<br>974	103<br>.2	95.<br>4	99.<br>1	
R_Date AbricRomani_A_OxA-11967	41541	40469	68.268949	41961	39900	95.449<br>974	41493	40485	68.268<br>949	41857	39895	95.449<br>974	103<br>.1	95.<br>4	99.<br>3	
R_Date Arbreda_H_OxA-21784	41722	40535	68.268949	42087	39855	95.449<br>974	41617	40511	68.268<br>949	41930	39832	95.449<br>974	103<br>.8	95.<br>4	99.<br>5	
R_Date LapadoPicareiro_GGII-II_MAMS-42278	41588	41181	68.268949	41826	41055	95.449<br>974	41564	41180	68.268<br>949	41800	41045	95.449<br>974	103<br>.4	95.<br>7	99.<br>7	
R_Date AbricRomani_A_NzA-2311	41976	41126	68.268949	42273	40597	95.449<br>974	41804	41060	68.268<br>949	42080	40532	95.449<br>974	103<br>.5	95.<br>4	99.<br>4	
R_Date LapadoPicareiro_GGII-II_MAMS-42282	41808	41401	68.268949	41965	41240	95.449<br>974	41737	41360	68.268<br>949	41919	41211	95.449<br>974	102<br>.4	95.<br>6	99.<br>5	
R_Date AbricRomani_A_AA-6608	42032	41226	68.268949	42319	40726	95.449<br>974	41829	41136	68.268<br>949	42100	40649	95.449<br>974	102<br>.4	95.<br>4	99.<br>4	
R_Date Arbreda_H_AA-3379	42606	41495	68.268949	43783	40767	95.449<br>973	41945	41209	68.268<br>949	42295	40661	95.449<br>974	81.<br>4	94.<br>7	99.<br>2	
Phase Aurignacian																
	Boundary Start Aurignacian Mesomediterranean Iberia						42157	41646	68.268<br>949	42523	41452	95.449<br>974			97.<br>2	
Sequence																
N(0	2)	-2.06	2.06	68.268<br>949	-4	4	95.449<br>974									10<br>0
Outlier_Model SSimple															10	

																0	
U(0	4)		3.99E-17	4	68.268949	3.99E-17	4	95.449974	5.38E-17	2.3	68.268949	5.38E-17	3.54	95.449974	100		99.8
T(5)		-1.14	1.14	68.268949	-2.65	2.65	95.449974									99.9	
Outlier_Model General								-129	127	68.268949	-1382	1303	95.449973			100	

Curve IntCal20

Summary

Validation and sensitivity tests

After running the model with all dates (Model a), three outliers are detected: Aguilon_P5_MAMS-28122 (A=30.5), PeñaMiel_e_GrN-12123 (A= 26.1), R_Date LosMoros_ GrN-12809 (A=45.9%), and particularly GrutadoCaldeirao_5_MAMS-41874 (A=9.1%) and R_Date GrutadoCaldeirao_6_MAMS-41876 (A=6%) (Table 5). Accordingly, the model agreement is low (A=48.8). However, if these outlier dates are removed from the model, the boundary for the end of the Mousterian technocomplex remains similar (from 40.31 to 40.80 ka cal BP) (Table 5), but the general model agreement is higher (Table 5). If the oldest and youngest dates are removed, the end of the Mousterian is 300 years older. Therefore, these results support that the Mousterian technocomplex disappeared during the transition between the Greenland Interstadial 10 and the Greenland Stadial 10. Likewise, the Aurignacian culture arrives during the transition between the Greenland Stadial 9 and the Greenland Interstadial 10, even if the oldest and youngest dates are excluded from the models (Table 5).

Mesomediterranean region

**Culture**	**Boundary**	**Model**	**68.2% prob.**		**95.54% prob.**		**A. model**
			From	to	From	to	
Mousterian	End	a. All dates	41.67	41.04	41.98	40.31	48.8
		b. Excluding outliers	41.69	41.16	41.88	40.80	100.7
		c. Excluding the oldest and youngest dates	41.89	41.39	42.06	41.01	100.5
Aurignacian	Start	a. All dates	41.23	41.69	42.62	41.48	100
		b. Excluding the oldest and youngest dates	42.15	41.64	42.52	41.45	102.5
	End	a. All dates	36.69	36.08	36.97	35.69	100
		b. Excluding the oldest and youngest dates	36.79	36.18	37.09	35.78	102.5

Table 5. Results of the 68.2% and 95.4% PDF rage of each techno-complex in the Mesomediterranean region.

Mesomediterranean region

**Culture**	**Boundary**	**Model**	**68.2% prob.**		**95.54% prob.**		**A. model**
			From	to	From	to	
Mousterian	End	1	41.69	41.16	41.88	40.80	100.7
		2	41.72	41.18	41.95	40.99	95.5
		3	41.66	41.16	41.91	40.77	104.3

		4	41.70	41.21	41.85	40.81	99.4
Aurignacian	Start	1	41.23	41.69	42.62	41.48	100
		2	41.20	41.74	42.57	41.50	98.2
		3	41.22	41.70	42.60	41.51	104.1
		4	41.25	41.67	42.61	41.47	99.9
	End	1	36.69	36.08	36.97	35.69	100
		2	36.72	36.09	36.96	35.71	98.2
		3	36.66	36.06	36.95	35.70	104.1
		4	36.68	36.07	36.98	35.66	99.9

Table 6. Results obtained when the Bayesian age model for the Mousterian, Châtelperronian and Aurignacian techno-complesexes if the each model is run four different times.

2.4. Thermomediterranean

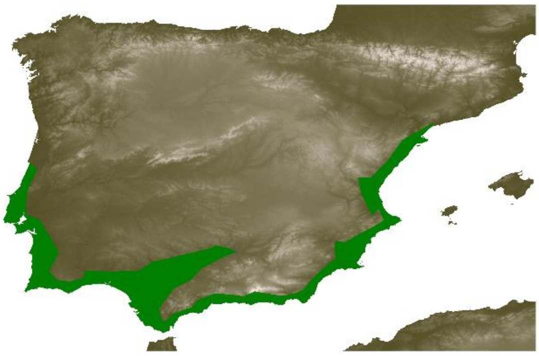

Figure 17. The Iberian Peninsula with the Thermomediterranean biogeographic region shaded in green.

2.4.1. End Mousterian

a. All dates

```
Options()
 {
  Curve("IntCal20","intcal20.14c");
  BCAD=FALSE;
  SD1=TRUE;
  SD2=TRUE;
  ConvergenceData=TRUE;
  kIterations=300;
 };
Plot()
{
  Outlier_Model("General",T(5),U(0,4),"t");
Outlier_Model("SSimple",N(0,2),0,"s");
  Sequence()
  {
   Boundary("End Mousterian");
```

```
   Phase("End Mousterian")
{
R_Date("Bajondillo_CNA-3822.1.1",48410,2420)
{
Outlier("General", 0.05);
};
R_Date("Bajondillo_CNA-4168.1.1",46890,180)
{
Outlier("General", 0.05);
};
R_Date("VanguardCave_OxA-8526",46700,1900)
{
Outlier("General", 0.05);
};
R_Date("Bajondillo_CNA-3880.1.1",46610,1740)
{
Outlier("General", 0.05);
};
R_Date("Zafarraya_OxA-21810", 46300,2500)
{
Outlier("General", 0.05);
};
Age("ElSalt_V_OSL2", N(45200, 3400))
   {
    Outlier("General", 0.05);
   };
Age("ElSalt_V_OSL1", N(44700, 3200))
   {
    Outlier("General", 0.05);
   };
R_Date("Bajondillo_CNA-3875.1.1",44020,1290)
{
Outlier("General", 0.05);
};
R_Date("Bajondillo_CNA-3211.1.1", 43840, 1300)
{
Outlier("General", 0.05);
};
R_Date("GorhamCave_OxA-8525",43800,1300)
{
Outlier("General", 0.05);
};
R_Date("Bajondillo_CNA-3881.1.1", 42710, 1120)
{
Outlier("General", 0.05);
};
R_Date("GorhamCave_ OxA-7791",42200,1100)
{
Outlier("General", 0.05);
};
R_Date("CuevaAnton_OxA-18672",39650,550)
{
Outlier("General", 0.05);
};
R_Date("LaBoja_VERA-5855",33170,450)
{
Outlier("General", 0.05);
};
R_Date("CuevaAnton_OxA-21244",32890,200)
```

```
{
Outlier(“General”, 0.05);
};
R_Date(“LaBoja_VERA-5850”,32890,410)
{
Outlier(“General”, 0.05);
};
R_Date(“CuevaAnton_OxA-22019”,32390,280)
{
Outlier(“General”, 0.05);
};
R_Date(“CuevaAnton_OxA-22625”,32330,250)
{
Outlier(“General”, 0.05);
};
R_Date(“CuevaAnton_OxA-26346”,31790,270)
{
Outlier(“General”, 0.05);
};
   };
   Boundary("Final End Mousterian");
  };
 };
};
```

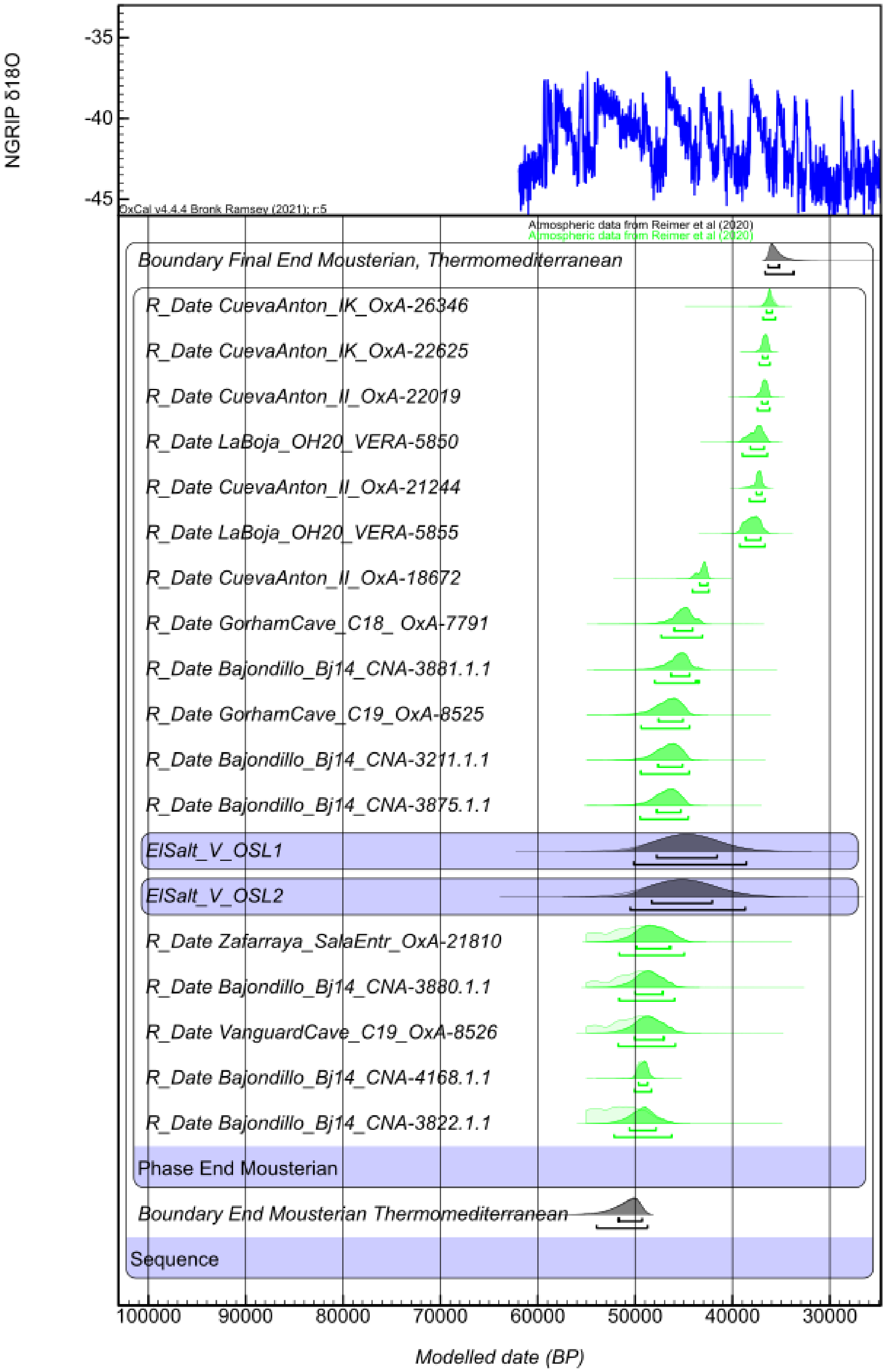

Figure 18. Plot of dated radiocarbon dates from Mousterian assemblages in the Thermomediterranean region

Name	Unmodelled (BP)						Modelled (BP)						Indices					
Amodel 107.7																		
Aoverall 107.9"																		
	from	to	%	from	to	%	from	to	%	from	to	%	Acomb	A	L	P	C	
Boundary Final End Mousterian	Thermomediterranean							36355	35184	68.268949	36655	33696	95.449974					98.8
R_Date CuevaAnton_IK_OxA-26346	36416	35821	68.268949	36737	35489	95.449974	36518	35947	68.268949	36875	35607	95.449974		99.4		95.1	99.7	
R_Date CuevaAnton_IK_OxA-22625	36910	36368	68.268949	37239	36161	95.449974	36920	36378	68.268949	37262	36165	95.449974		101.1		95.7	99.8	
R_Date CuevaAnton_II_OxA-22019	36992	36380	68.268949	37427	36184	95.449974	36997	36385	68.268949	37455	36191	95.449974		101.1		95.6	99.8	
R_Date LaBoja_OH20_VERA-5850	38158	36757	68.268949	38980	36425	95.449974	38166	36754	68.268949	38994	36423	95.449974		100.5		95.4	99.7	
R_Date CuevaAnton_II_OxA-21244	37557	37001	68.268949	38225	36690	95.449974	37561	36999	68.268949	38245	36669	95.449974		100.6		95.5	99.9	
R_Date LaBoja_OH20_VERA-5855	38611	37110	68.268949	39250	36699	95.449974	38666	37109	68.26895	39265	36674	95.449974		100.4		95.3	99.7	
R_Date CuevaAnton_II_OxA-18672	43365	42560	68.268949	44083	42460	95.449974	43382	42555	68.268949	44119	42447	95.449974		100.1		95.2	99.8	
R_Date GorhamCave_C18_ OxA-7791	45984	44128	68.268949	47307	43113	95.449974	46000	44115	68.268949	47316	43103	95.449974		100.2		95.1	99.6	
R_Date Bajondillo_Bj14_CNA-3881.1.1	46325	44420	68.268949	48004	43427	95.449973	46352	44423	68.268949	47984	43434	95.449973		100.4		95.2	99.6	
R_Date GorhamCave_C19_OxA-8525	47629	45077	68.268949	49869	44377	95.449974	47600	45109	68.268949	49386	44430	95.449974		102.6		95.2	99.5	
R_Date Bajondillo_Bj14_CNA-3211.1.1	47674	45115	68.268949	49902	44395	95.449974	47638	45152	68.268949	49426	44457	95.449974		102.6		95.2	99.5	
R_Date Bajondillo_Bj14_CNA-3875.1.1	47852	45281	68.268949	50025	44477	95.449974	47824	45316	68.26895	49483	44563	95.449974		103		95.2	99.5	
N(44700	3200)	41404	47996	68.268949	38300	51100	95.449974	41596	47804	68.268949	38588	50140	95.449974		103.4		95.2	100
#¿NOMBRE?							1950	1951	68.268949	1950	1951	95.449974					100	
ElSalt_V_OSL1	47996	41404	68.268949	51100	38300	95.449974	47804	41596	68.268949	50140	38588	95.449974					100	
N(45200	3400)	41698	48702	68.268949	38400	52000	95.449974	42106	48294	68.268949	38706	50504	95.449974		104.9		95.2	100
#¿NOMBRE?							1950	1951	68.2689	1950	1951	95.4499					10	

									49			74					0	
ElSalt_V_OSL2	48702	41698	68.268949	52000	38400	95.449974	48294	42106	68.268949	50504	38706	95.449974					100	
R_Date Zafarraya_SalaEntr_OxA-21810	52150	46593	68.268949	...	45920	95.449974	49897	46338	68.268949	51637	44957	95.449974		108.3		95.1	99.4	
R_Date Bajondillo_Bj14_CNA-3880.1.1	51851	47360	68.268949	...	46277	95.449973	49990	47152	68.268949	51653	45964	95.449973		112.3		95.1	99.4	
R_Date VanguardCave_C19_OxA-8526	52069	47328	68.268949	...	46511	95.449973	50022	47054	68.268948	51753	45876	95.449974		110.3		95.1	99.4	
R_Date Bajondillo_Bj14_CNA-4168.1.1	49734	48803	68.268949	50116	48371	95.449974	49650	48731	68.268949	50056	48330	95.449974		101.1		95.4	99.8	
R_Date Bajondillo_Bj14_CNA-3822.1.1	54795	49540	68.26895	...	47753	95.449974	50573	47863	68.268949	52179	46253	95.449974		84.8		94.9	99.3	
Phase End Mousterian																		
Boundary End Mousterian Thermomediterranean							51758	49290	68.268949	53979	48739	95.449974					97.4	
Sequence																		
N(0	2)	-2.06	2.06	68.268949	-4	4	95.449974											99.9
Outlier_Model SSimple							-2	3	68.268949	-2	3	95.449974					100	
U(0	4)	3.99E-17	4	68.268949	3.99E-17	4	95.449974	5.38E-17	2.62	68.268949	5.38E-17	3.748	95.449974		100			100
T(5)	-1.14	1.14	68.268949	-2.65	2.65	95.449974											99.9	
Outlier_Model General							-223	222	68.268949	-2944	2713	95.449974					100	
Curve IntCal20																		

b. **Excluding dates performed on shells**

```
Options()
 {
  Curve("IntCal20","intcal20.14c");
  BCAD=FALSE;
  SD1=TRUE;
  SD2=TRUE;
  ConvergenceData=TRUE;
  kIterations=300;
 };
Plot()
{
  Outlier_Model("General",T(5),U(0,4),"t");
Outlier_Model("SSimple",N(0,2),0,"s");
  Sequence()
  {
    Boundary("End Mousterian");
    Phase("End Mousterian")
{
R_Date(“VanguardCave_OxA-8526”,46700,1900)
{
Outlier(“General”, 0.05);
};
R_Date(“Zafarraya_OxA-21810”, 46300,2500)
{
Outlier(“General”, 0.05);
};
Age("ElSalt_V_OSL2", N(45200, 3400))
      {
        Outlier("General", 0.05);
      };
Age("ElSalt_V_OSL1", N(44700, 3200))
      {
        Outlier("General", 0.05);
      };
R_Date(“GorhamCave_OxA-8525”,43800,1300)
{
Outlier(“General”, 0.05);
};
R_Date(“GorhamCave_OxA-7791”,42200,1100)
{
Outlier(“General”, 0.05);
};
R_Date(“CuevaAnton_OxA-18672”,39650,550)
{
Outlier(“General”, 0.05);
};
R_Date(“LaBoja_VERA-5855”,33170,450)
{
Outlier(“General”, 0.05);
};
R_Date(“CuevaAnton_OxA-21244”,32890,200)
{
Outlier(“General”, 0.05);
};
R_Date(“LaBoja_VERA-5850”,32890,410)
```

```
{
Outlier(“General”, 0.05);
};
R_Date(“CuevaAnton_OxA-22019”,32390,280)
{
Outlier(“General”, 0.05);
};
R_Date(“CuevaAnton_OxA-22625”,32330,250)
{
Outlier(“General”, 0.05);
};
R_Date(“CuevaAnton_OxA-26346”,31790,270)
{
Outlier(“General”, 0.05);
};
   };
   Boundary("Final End Mousterian");
  };
 };
};
```

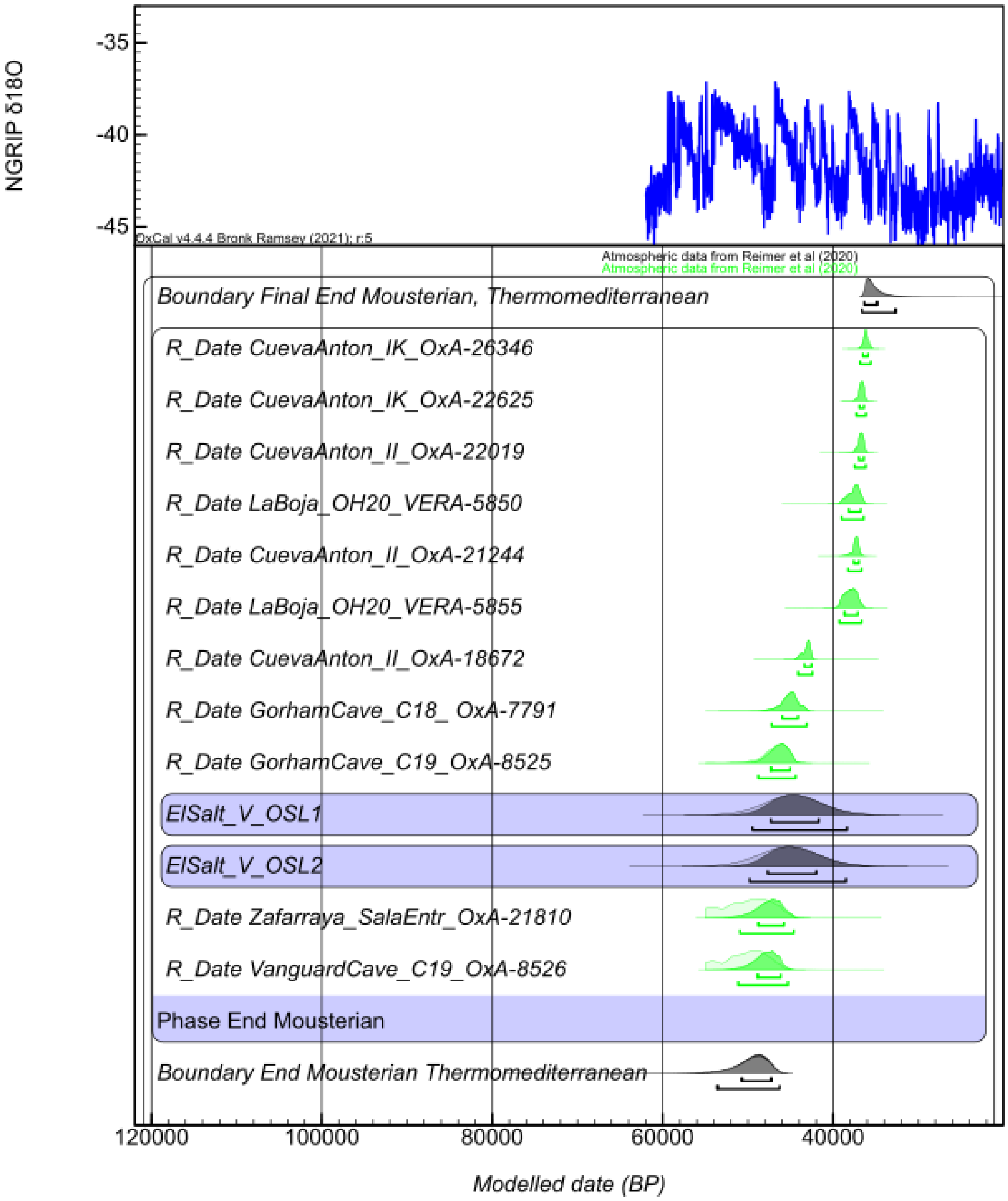

Figure 19. Plot of dated radiocarbon dates from Mousterian assemblages in the Thermomediterranean region after excluding the oldest and youngest dates.

Name	Unmodelled (BP)						Modelled (BP)						Indices					
Amodel 105.3																		
Aoverall 105.3"																		
	from	to	%	from	to	%	from	to	%	from	to	%	Acomb	A	L	P	C	
Boundary Final End Mousterian	Thermomediterran<br>ean							36324	34846	68.2689<br>5	36628	32649	95.4499<br>74					97.<br>8
R_Date CuevaAnton_IK_OxA-<br>26346	36416	35821	68.2689<br>49	36737	35489	95.4499<br>74	36498	35920	68.2689<br>49	36845	35580	95.4499<br>74		100.<br>7		95.<br>5	99.<br>8	
R_Date CuevaAnton_IK_OxA-<br>22625	36910	36368	68.2689<br>49	37239	36161	95.4499<br>74	36916	36373	68.2689<br>49	37265	36160	95.4499<br>74		101.<br>1		95.<br>7	99.<br>9	
R_Date CuevaAnton_II_OxA-<br>22019	36992	36380	68.2689<br>49	37427	36184	95.4499<br>74	36999	36387	68.2689<br>49	37457	36186	95.4499<br>74		101		95.<br>5	99.<br>8	
R_Date LaBoja_OH20_VERA-<br>5850	38158	36757	68.2689<br>49	38980	36425	95.4499<br>74	38178	36757	68.2689<br>49	39000	36412	95.4499<br>74		100.<br>5		95.<br>3	99.<br>7	
R_Date CuevaAnton_II_OxA-<br>21244	37557	37001	68.2689<br>49	38225	36690	95.4499<br>74	37561	36997	68.2689<br>49	38251	36664	95.4499<br>74		100.<br>6		95.<br>5	99.<br>8	
R_Date LaBoja_OH20_VERA-<br>5855	38611	37110	68.2689<br>49	39250	36699	95.4499<br>74	38640	37114	68.2689<br>49	39264	36661	95.4499<br>74		100.<br>3		95.<br>3	99.<br>7	
R_Date CuevaAnton_II_OxA-<br>18672	43365	42560	68.2689<br>49	44083	42460	95.4499<br>74	43380	42556	68.2689<br>49	44124	42445	95.4499<br>74		100.<br>2		95.<br>2	99.<br>7	
R_Date GorhamCave_C18_ OxA-<br>7791	45984	44128	68.2689<br>49	47307	43113	95.4499<br>74	45978	44143	68.2689<br>49	47227	43101	95.4499<br>74		101.<br>2		95.<br>2	99.<br>6	
R_Date GorhamCave_C19_OxA-<br>8525	47629	45077	68.2689<br>49	49869	44377	95.4499<br>74	47353	45063	68.2689<br>5	48803	44393	95.4499<br>74		107.<br>5		95.<br>2	99.<br>5	
N(44700	3200)	41404	47996	68.2689<br>49	38300	51100	95.4499<br>74	41692	47324	68.2689<br>49	38364	49468	95.4499<br>74		106.<br>2	95.<br>2		10<br>0
#¿NOMBRE?							1950	1951	68.2689<br>49	1950	1951	95.4499<br>74					10<br>0	
ElSalt_V_OSL1	47996	41404	68.2689<br>49	51100	38300	95.4499<br>74	47324	41692	68.2689<br>49	49468	38364	95.4499<br>74					10<br>0	
N(45200	3400)	41698	48702	68.2689<br>49	38400	52000	95.4499<br>74	41970	47682	68.2689<br>49	38502	49790	95.4499<br>74		107.<br>5	95.<br>3		10<br>0
#¿NOMBRE?							1950	1951	68.2689<br>49	1950	1951	95.4499<br>74					10<br>0	
ElSalt_V_OSL2	48702	41698	68.2689<br>49	52000	38400	95.4499<br>74	47682	41970	68.2689<br>49	49790	38502	95.4499<br>74					10<br>0	
R_Date Zafarraya_SalaEntr_OxA-<br>21810	52150	46593	68.2689<br>49	...	45920	95.4499<br>74	48780	45779	68.2689<br>5	50935	44627	95.4499<br>74		99.7		94.<br>9	99.<br>3	
R_Date VanguardCave_C19_OxA-	52069	47328	68.2689	...	46511	95.4499	48908	46166	68.2689	51147	45317	95.4499		94		94.	99.	

8526				49			73			49			74			5	2	
Phase End Mousterian																		
Boundary End Mousterian Thermomediterranean								50781	47232	68.268949	53617	46287	95.449973				96	
Sequence																		
N(0	2)		-2.06	2.06	68.268949	-4	4	95.449974										99.9
Outlier_Model SSimple								-2	3	68.268949	-2	3	95.449974				100	
U(0	4)		3.99E-17	4	68.268949	3.99E-17	4	95.449974	5.38E-17	2.74	68.268949	5.38E-17	3.776	95.449974	100			99.9
T(5)		-1.14	1.14	68.268949	-2.65	2.65	95.449974										99.8	
Outlier_Model General								-240	243	68.268949	-3273	2614	95.449974				100	
Curve IntCal20																		

c. Excluding level I-K from Cueva Antón

```
Options()
 {
  Curve("IntCal20","intcal20.14c");
  BCAD=FALSE;
  SD1=TRUE;
  SD2=TRUE;
  ConvergenceData=TRUE;
  kIterations=300;
 };
Plot()
{
 Outlier_Model("General",T(5),U(0,4),"t");
Outlier_Model("SSimple",N(0,2),0,"s");
 Sequence()
 {
   Boundary("End Mousterian");
   Phase("End Mousterian")
{
R_Date(“VanguardCave_OxA-8526”,46700,1900)
{
Outlier(“General”, 0.05);
};
R_Date(“Zafarraya_OxA-21810”, 46300,2500)
{
Outlier(“General”, 0.05);
};
Age("ElSalt_V_OSL2", N(45200, 3400))
     {
      Outlier("General", 0.05);
     };
Age("ElSalt_V_OSL1", N(44700, 3200))
     {
      Outlier("General", 0.05);
     };
R_Date(“GorhamCave_OxA-8525”,43800,1300)
{
Outlier(“General”, 0.05);
};
R_Date(“GorhamCave_OxA-7791”,42200,1100)
{
Outlier(“General”, 0.05);
};
R_Date(“CuevaAnton_OxA-18672”,39650,550)
{
Outlier(“General”, 0.05);
};
R_Date(“LaBoja_VERA-5855”,33170,450)
{
Outlier(“General”, 0.05);
};
R_Date(“CuevaAnton_OxA-21244”,32890,200)
{
Outlier(“General”, 0.05);
};
R_Date(“LaBoja_VERA-5850”,32890,410)
{
```

```
Outlier("General", 0.05);
};
R_Date("CuevaAnton_OxA-22019",32390,280)
{
Outlier("General", 0.05);
};
  };
  Boundary("Final End Mousterian, Thermomediterranean");
 };
 };
};
```

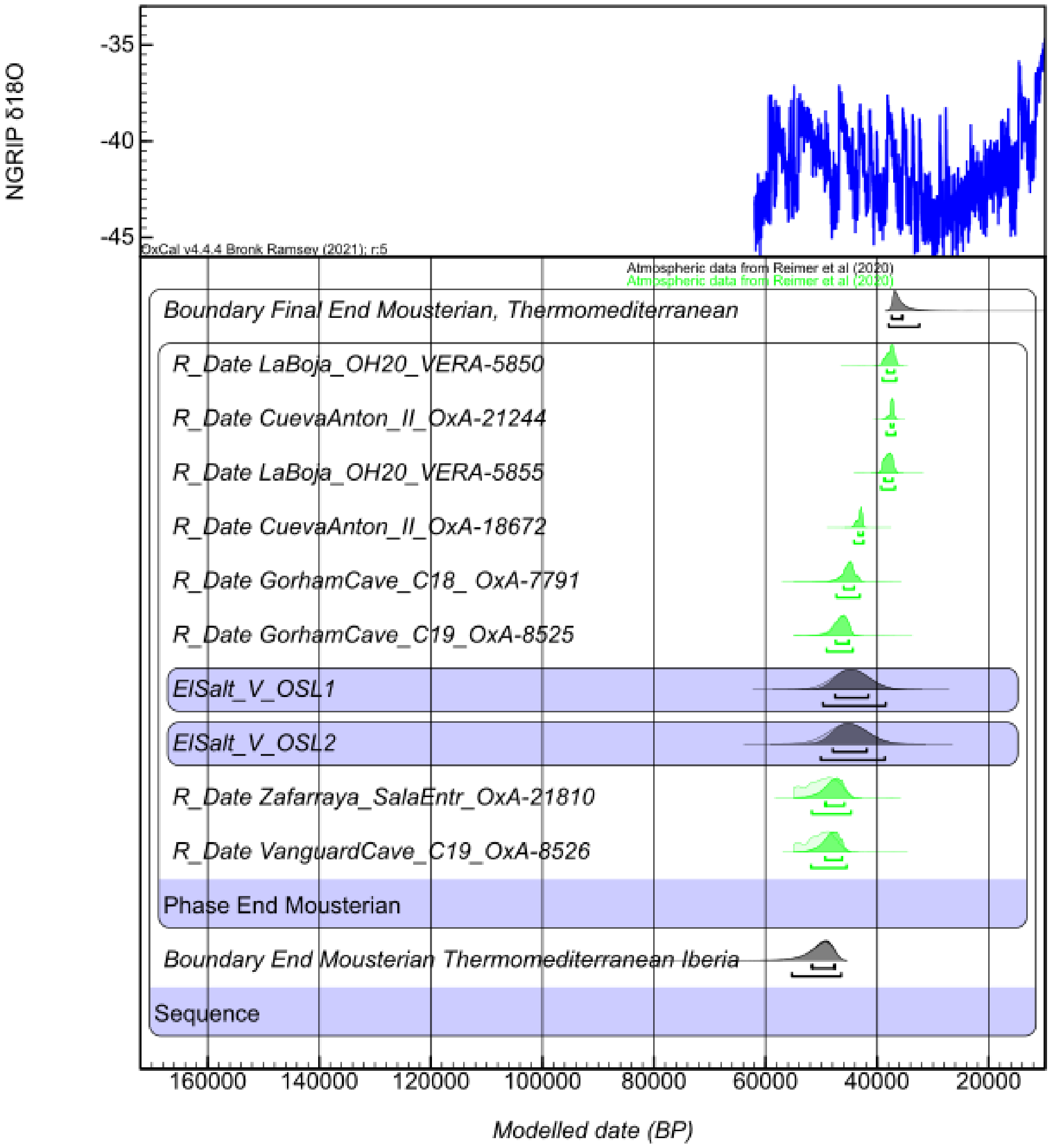

Figure 20. Plot of dated radiocarbon dates from Mousterian assemblages in the Thermomediterranean region after excluding the level I-K from Cueva Antón.

Name	Unmodelled (BP)						Modelled (BP)						Indices					
Amodel 106.6																		
Aoverall 106.4"																		
	from	to	%	from	to	%	from	to	%	from	to	%	Acomb	A	L	P	C	
Boundary Final End Mousterian	Thermomediterranean							37373	35445	68.268949	37938	32443	95.449974					98.7
R_Date LaBoja_OH20_VERA-5850	38158	36757	68.268949	38980	36425	95.449974	38269	36896	68.268949	39055	36535	95.449974		100.4		95.4	99.8	
R_Date CuevaAnton_II_OxA-21244	37557	37001	68.268949	38225	36690	95.449974	37592	37013	68.268949	38352	36737	95.449974		98.6		95.5	99.9	
R_Date LaBoja_OH20_VERA-5855	38611	37110	68.268949	39250	36699	95.449974	38671	37192	68.268949	39281	36787	95.449974		101.9		95.4	99.9	
R_Date CuevaAnton_II_OxA-18672	43365	42560	68.268949	44083	42460	95.449974	43378	42555	68.268949	44128	42445	95.449974		100.2		95.2	99.9	
R_Date GorhamCave_C18_ OxA-7791	45984	44128	68.268949	47307	43113	95.449974	45982	44129	68.268949	47288	43107	95.449974		100.9		95.2	99.8	
R_Date GorhamCave_C19_OxA-8525	47629	45077	68.268949	49869	44377	95.449974	47401	45064	68.26895	49048	44370	95.449974		105.8		95.2	99.8	
N(44700	3200)	41404	47996	68.268949	38300	51100	95.449974	41596	47516	68.268949	38492	49692	95.449974		105.4		95.2	100
#¿NOMBRE?							1950	1951	68.268949	1950	1951	95.449974					100	
ElSalt_V_OSL1	47996	41404	68.268949	51100	38300	95.449974	47516	41596	68.268949	49692	38492	95.449974					100	
N(45200	3400)	41698	48702	68.268949	38400	52000	95.449974	41868	47920	68.268949	38604	50096	95.449974		106.7		95.2	100
#¿NOMBRE?							1950	1951	68.268949	1950	1951	95.449974					100	
ElSalt_V_OSL2	48702	41698	68.268949	52000	38400	95.449974	47920	41868	68.268949	50096	38604	95.449974					100	
R_Date Zafarraya_SalaEntr_OxA-21810	52150	46593	68.268949	...	45920	95.449974	49219	45871	68.268949	51680	44687	95.449974		102.2		95	99.7	
R_Date VanguardCave_C19_OxA-8526	52069	47328	68.268949	...	46511	95.449973	49290	46285	68.268949	51816	45436	95.449974		99		94.8	99.7	
Phase End Mousterian																		
Boundary End Mousterian Thermomediterranean Iberia							51673	47571	68.268948	55289	46457	95.449974					97.2	

Sequence

N(0	2)		-2.06	2.06	68.2689<br>49	-4	4	95.4499<br>74									10<br>0
Outlier_Model SSimple								-2	3	68.2689<br>49	-2	3	95.4499<br>74			10<br>0	
U(0	4)		3.99E-17	4	68.2689<br>49	3.99E-<br>17	4	95.4499<br>74	5.38E-<br>17	2.8	68.2689<br>49	5.38E-<br>17	3.792	95.4499<br>74	100		10<br>0
T(5)		-1.14	1.14	68.2689<br>49	-2.65	2.65	95.4499<br>74									99.<br>9	
Outlier_Model General								-276	275	68.2689<br>49	-3772	3195	95.4499<br>73			10<br>0	

Curve IntCal20

d. Without the oldest and youngest dates

```
Options()
 {
  Curve("IntCal20","intcal20.14c");
  BCAD=FALSE;
  SD1=TRUE;
  SD2=TRUE;
  ConvergenceData=TRUE;
  kIterations=300;
 };
Plot()
{
  Outlier_Model("General",T(5),U(0,4),"t");
Outlier_Model("SSimple",N(0,2),0,"s");
  Sequence()
  {
    Boundary("End Mousterian");
    Phase("End Mousterian")
{
R_Date("VanguardCave_OxA-8526",46700,1900)
{
Outlier("General", 0.05);
};
R_Date("Zafarraya_OxA-21810", 46300,2500)
{
Outlier("General", 0.05);
};
Age("ElSalt_V_OSL2", N(45200, 3400))
      {
        Outlier("General", 0.05);
      };
Age("ElSalt_V_OSL1", N(44700, 3200))
      {
        Outlier("General", 0.05);
      };
R_Date("GorhamCave_OxA-8525",43800,1300)
{
Outlier("General", 0.05);
};
R_Date("GorhamCave_OxA-7791",42200,1100)
{
Outlier("General", 0.05);
};
R_Date("CuevaAnton_OxA-18672",39650,550)
{
Outlier("General", 0.05);
};
R_Date("LaBoja_VERA-5855",33170,450)
{
Outlier("General", 0.05);
};
R_Date("CuevaAnton_OxA-21244",32890,200)
{
Outlier("General", 0.05);
};
R_Date("LaBoja_VERA-5850",32890,410)
{
```

```
Outlier(“General”, 0.05);
};
R_Date(“CuevaAnton_OxA-22019”,32390,280)
{
Outlier(“General”, 0.05);
};
   };
   Boundary("Final End Mousterian");
  };
 };
};
```

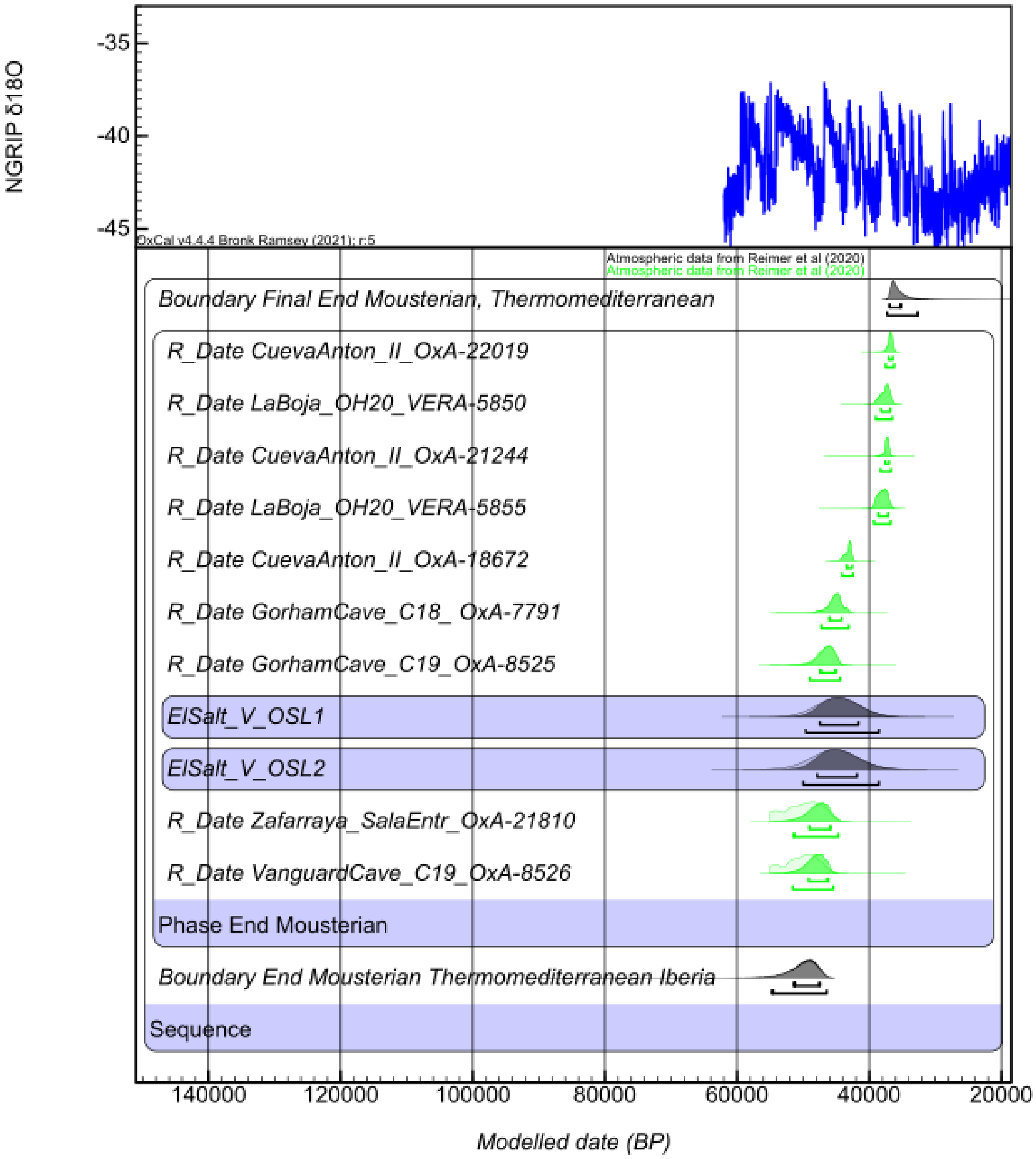

Figure 21. Plot of dated radiocarbon dates from Mousterian assemblages in the Thermomediterranean region after excluding the youngest and oldest dates

Name	Unmodelled (BP)						Modelled (BP)						Indices					
Amodel 105.5																		
Aoverall 105.7"																		
	from	to	%	from	to	%	from	to	%	from	to	%	Acomb	A	L	P	C	
Boundary Final End Mousterian	Thermomediterranean							36926	35170	68.268948	37306	32583	95.449973					97.7
R_Date CuevaAnton_II_OxA-22019	36992	36380	68.268949	37427	36184	95.449974	37075	36428	68.268949	37516	36212	95.449974		97.3		95.4	99.8	
R_Date LaBoja_OH20_VERA-5850	38158	36757	68.268949	38980	36425	95.449974	38185	36803	68.268949	39017	36473	95.449974		101.4		95.5	99.7	
R_Date CuevaAnton_II_OxA-21244	37557	37001	68.268949	38225	36690	95.449974	37564	37002	68.268949	38255	36679	95.449974		100.8		95.4	99.8	
R_Date LaBoja_OH20_VERA-5855	38611	37110	68.268949	39250	36699	95.449974	38618	37110	68.268948	39266	36712	95.449974		101		95.3	99.7	
R_Date CuevaAnton_II_OxA-18672	43365	42560	68.268949	44083	42460	95.449974	43386	42557	68.268949	44111	42446	95.449974		100.4		95.4	99.8	
R_Date GorhamCave_C18_ OxA-7791	45984	44128	68.268949	47307	43113	95.449974	45994	44133	68.268949	47243	43104	95.449974		101		95.2	99.6	
R_Date GorhamCave_C19_OxA-8525	47629	45077	68.268949	49869	44377	95.449974	47391	45055	68.268949	48973	44378	95.449974		106.4		95.2	99.5	
N(44700	3200)	41404	47996	68.268949	38300	51100	95.449974	41628	47452	68.268949	38524	49596	95.449974		105.6	95.2		100
#¿NOMBRE?							1950	1951	68.268949	1950	1951	95.449974					100	
ElSalt_V_OSL1	47996	41404	68.268949	51100	38300	95.449974	47452	41628	68.268949	49596	38524	95.449974					100	
N(45200	3400)	41698	48702	68.268949	38400	52000	95.449974	41834	47886	68.268949	38536	49994	95.449974		106.9	95.2		100
#¿NOMBRE?							1950	1951	68.268949	1950	1951	95.449974					100	
ElSalt_V_OSL2	48702	41698	68.268949	52000	38400	95.449974	47886	41834	68.268949	49994	38536	95.449974					100	
R_Date Zafarraya_SalaEntr_OxA-21810	52150	46593	68.268949	...	45920	95.449974	49045	45831	68.268949	51458	44666	95.449973		101.5		94.9	99.3	
R_Date VanguardCave_C19_OxA-8526	52069	47328	68.268949	...	46511	95.449973	49135	46200	68.268949	51590	45404	95.449974		97.4		94.7	99.3	
Phase End Mousterian																		
Boundary End Mousterian Thermomediterranean Iberia							51419	47458	68.268949	54700	46402	95.449974					95.6	

Sequence																	
N(0	2)		-2.06	2.06	68.2689<br>49	-4	4	95.4499<br>74									99.<br>9
Outlier_Model SSimple								-2	3	68.2689<br>49	-2	3	95.4499<br>74			10<br>0	
U(0	4)		3.99E-17	4	68.2689<br>49	3.99E-<br>17	4	95.4499<br>74	5.38E-<br>17	2.784	68.2689<br>49	5.38E-<br>17	3.788	95.4499<br>74	100		10<br>0
T(5)		-1.14	1.14	68.2689<br>49	-2.65	2.65	95.4499<br>74									99.<br>8	
Outlier_Model General								-266	264	68.2689<br>49	-3499	3031	95.4499<br>74			10<br>0	
Curve IntCal20																	

2.4.2. Châtelperronian

```
Options()
 {
  Curve("IntCal20","intcal20.14c");
  BCAD=FALSE;
  SD1=TRUE;
  SD2=TRUE;
  ConvergenceData=TRUE;
  kIterations=300;
 };
Plot()
{
  Outlier_Model("General",T(5),U(0,4),"t");
Outlier_Model("SSimple",N(0,2),0,"s");
  Sequence()
  {
    Boundary("Start Châtelperronian");
    Phase("Châtelperronian")
{
R_Date("CovaForadada_Beta-435465",34570,240)
{
Outlier("General", 0.05);
};
R_Date("CovaForadada_OxA-X-2649-9",34490,320)
{
Outlier("General", 0.05);
};
R_Date("CovaForadada_OxA-X-2650-9",34300,1000)
{
Outlier("General", 0.05);
};
    };
    Boundary("End Châtelperronian");
   };
  };
 };
```

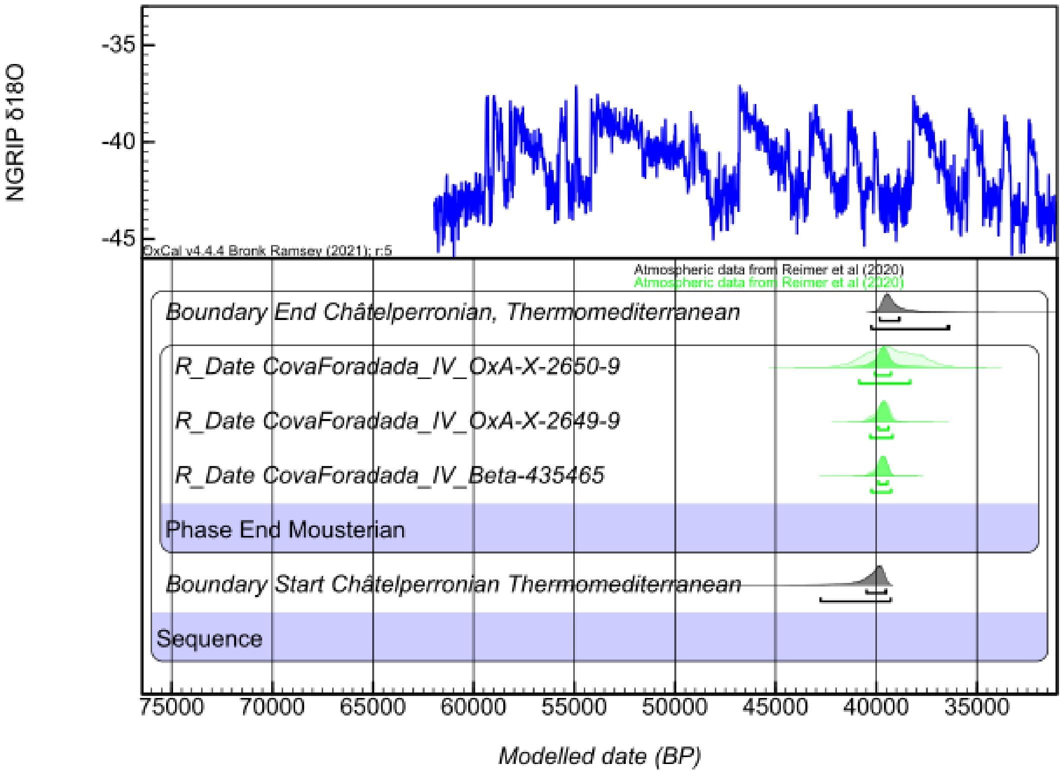

Figure 22. Plot of dated radiocarbon dates from Châtelperronian assemblages in the Thermomediterranean region

Name	Unmodelled (BP)						Modelled (BP)						Indices					
Amodel 129.1																		
Aoverall 126"																		
	from	to	%	from	to	%	from	to	%	from	to	%	Acomb	A	L	P	C	
Boundary End ChÃ¢telperronian	Thermomediterranean							39830	38858	68.26895	40242	36373	95.449973					97.2
R_Date CovaForadada_IV_OxA-X-2650-9	40574	38068	68.268949	41415	36925	95.449974	40060	39276	68.268949	40845	38313	95.449974		128.3		95.8	99.6	
R_Date CovaForadada_IV_OxA-X-2649-9	39957	39311	68.268949	40476	39119	95.449974	39892	39395	68.268949	40297	39190	95.449974		113.3		96.1	99.9	
R_Date CovaForadada_IV_Beta-435465	39948	39426	68.268949	40352	39268	95.449974	39885	39445	68.268949	40231	39268	95.449974		109.1		96.1	99.9	
Phase End Mousterian																		
Boundary Start ChÃ¢telperronian Thermomediterranean							40486	39509	68.268949	42769	39288	95.449974					96.9	
Sequence																		
N(0	2)	-2.06	2.06	68.268949	-4	4	95.449974											100
Outlier_Model SSimple																	100	
U(0	4)	3.99E-17	4	68.268949	3.99E-17	4	95.449974	5.38E-17	2.66	68.268949	5.38E-17	3.88	95.449974		100			100
T(5)	-1.14	1.14	68.268949	-2.65	2.65	95.449974											99.4	
Outlier_Model General							-99	100	68.268949	-854	907	95.449973					100	
Curve IntCal20																		

2.4.3. Aurignacian

a. All dates

```
Options()
 {
  Curve("IntCal20","intcal20.14c");
  BCAD=FALSE;
  SD1=TRUE;
  SD2=TRUE;
  ConvergenceData=TRUE;
  kIterations=300;
 };
Plot()
{
  Outlier_Model("General",T(5),U(0,4),"t");
Outlier_Model("SSimple",N(0,2),0,"s");
  Sequence()
  {
    Boundary("Start Aurignacian");
    Phase("Aurignacian")
{
      R_Date("Bajondillo_CNA-3218.1.2", 38160, 230)
      {
        Outlier("General", 0.05);
      };
      R_Date("Bajondillo_CNA3882.1.2", 37430, 570)
      {
        Outlier("General", 0.05);
      };
      R_Date("Bajondillo_CNA3213.3.2", 37005, 1790)
      {
        Outlier("General", 0.05);
      };
      R_Date("Bajondillo_CNA-3216.3.1", 36890, 210)
      {
        Outlier("General", 0.05);
      };
R_Date("Bajondillo_CNA3873.1.1",36890, 200)
      {
        Outlier("General", 0.05);
      };

R_Date("PegodoDiablo_OxA-X-2272-25",35050, 750)
      {
        Outlier("General", 0.05);
      };
R_Date("PegodoDiablo_OxA-15499",34900, 1000)
      {
        Outlier("General", 0.05);
      };
R_Date("CovadelesMalladetes_VERA-6514AboxSc",33370, 390)
      {
        Outlier("General", 0.05);
      };
R_Date("CovadelesMalladetes_VERA-6514",33300, 440)
      {
        Outlier("General", 0.05);
```

```
    };
R_Date("LaBoja_VERA-6157",33290, 466)
    {
     Outlier("General", 0.05);
    };
R_Date("LaBoja_VERA-6157AboxSc",33179, 455)
    {
     Outlier("General", 0.05);
    };
R_Date("CovaForadada_OxA-34233 ",33170, 370)
    {
     Outlier("General", 0.05);
    };
R_Date("CovadelesMalladetes_VERA-6513ABOxSC",32860, 380)
    {
     Outlier("General", 0.05);
    };
R_Date("Bajondillo_CNA-3876.1.1",32780, 340)
    {
     Outlier("General", 0.05);
    };
R_Date("CovadelesMalladetes_VERA-6513",32560, 370)
    {
     Outlier("General", 0.05);
    };
R_Date("CovadelesMalladetes_VERA-6511ABOxSC",32400, 360)
    {
     Outlier("General", 0.05);
    };
R_Date("LaBoja_VERA-6158HS",32231, 417)
    {
     Outlier("General", 0.05);
    };
R_Date("CovadelesMalladetes_VERA-VERA-6511",32160, 360)
    {
     Outlier("General", 0.05);
    };
R_Date("CovadelesMalladetes_VERA-VERA-6510",32120, 360)
    {
     Outlier("General", 0.05);
    };
R_Date("CovadelesMalladetes_VERA-6510ABOxSC",32080, 350)
    {
     Outlier("General", 0.05);
    };
R_Date("LaBoja_VERA-5854",32080, 400)
    {
     Outlier("General", 0.05);
    };
R_Date("CovaForadada_OxA-34251",32050, 550)
    {
     Outlier("General", 0.05);
    };
R_Date("CovadelesMalladetes_VERA-6512",31880, 350)
    {
     Outlier("General", 0.05);
    };
R_Date("CovaForadada_Beta-37881",31690, 180)
    {
```

```
    Outlier("General", 0.05);
   };
R_Date("Bajondillo_CNA-3883 1.1",31280, 280)
   {
    Outlier("General", 0.05);
   };
R_Date("LaBoja_VERA-6156",30918, 343)
   {
    Outlier("General", 0.05);
   };
R_Date("CovaForadada_Beta-414540",30770, 180)
   {
    Outlier("General", 0.05);
   };
R_Date("CovaForadada_MAMS-33909 ",30760, 150)
   {
    Outlier("General", 0.05);
   };
R_Date("PegodoDiablo_VERA-4050",30260, 320)
   {
    Outlier("General", 0.05);
   };
R_Date("GorhamCave_OxA-7076",30250, 700)
   {
    Outlier("General", 0.05);
   };
R_Date("CovaForadada_Beta-37880",30220, 180)
   {
    Outlier("General", 0.05);
   };
R_Date("GorhamCave_OxA-7074",30200, 700)
   {
    Outlier("General", 0.05);
   };
R_Date("CovadelesMalladetes_VERA-6508",30100, 280)
   {
    Outlier("General", 0.05);
   };
R_Date("LaBoja_VERA-5854HS",30090, 310)
   {
    Outlier("General", 0.05);
   };
R_Date("PegodoDiablo_VERA-4049",29810, 300)
   {
    Outlier("General", 0.05);
   };
R_Date("GorhamCave_OxA-7075",29800, 700)
   {
    Outlier("General", 0.05);
   };
R_Date("CovadelesMalladetes_VERA-6509", 29520, 270)
   {
    Outlier("General", 0.05);
   };
R_Date("CovadelesMalladetes_VERA-6427ABOxSC", 29490, 260)
   {
    Outlier("General", 0.05);
   };
R_Date("CovadelesForadada_Beta-103781", 29440, 190)
```

```
    {
     Outlier("General", 0.05);
    };
R_Date("LaBoja_VERA-5853HS",29300, 290)
    {
     Outlier("General", 0.05);
    };
R_Date("CovadelesMalladetes_VERA-6427A", 29270, 260)
    {
     Outlier("General", 0.05);
    };
R_Date("GorhamCave_OxA-7077",29250, 650)
    {
     Outlier("General", 0.05);
    };
R_Date("LaBoja_VERA-6155HS",29230, 287)
    {
     Outlier("General", 0.05);
    };
   R_Date("Bajondillo_CNA-3817.1",29180, 240)
    {
     Outlier("General", 0.05);
    };
  R_Date("PegodoDiablo_VERA-4047",29090, 270)
    {
     Outlier("General", 0.05);
    };
   R_Date("Bajondillo_CNA-3878.1.1",29040, 220)
    {
     Outlier("General", 0.05);
    };
   };
   Boundary("End Aurignacian");
  };
 };
};
```

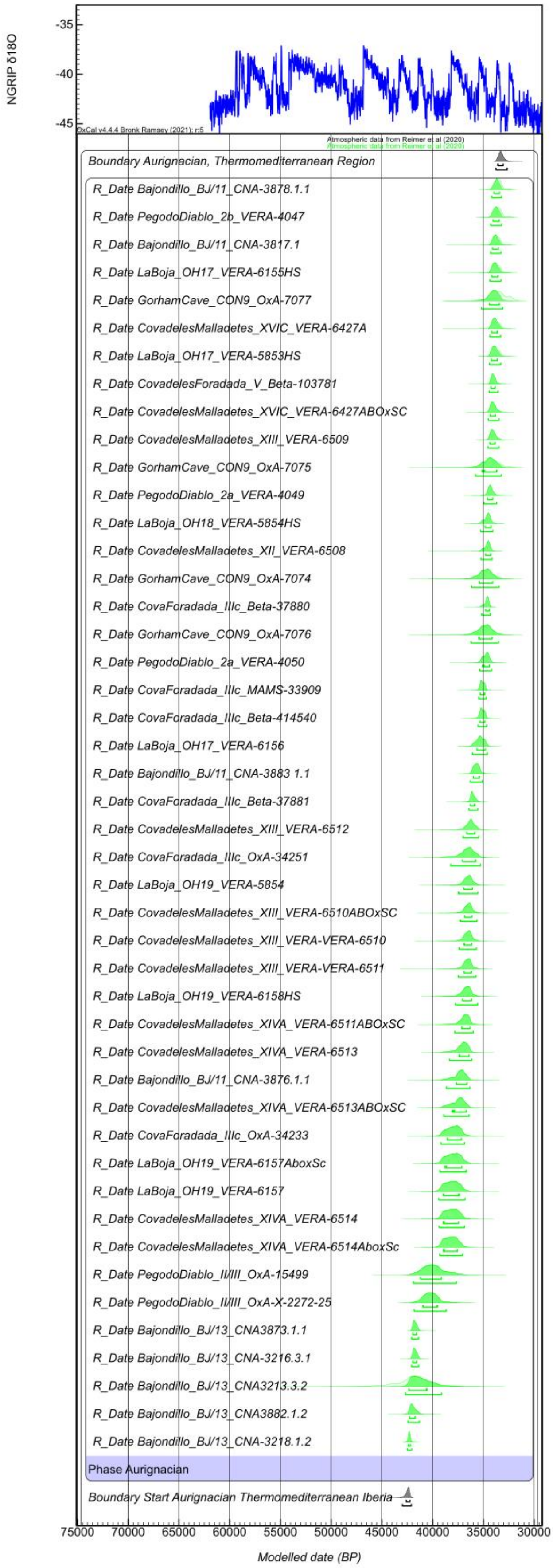

Figure 23. Plot of dated radiocarbon dates from Aurignacian assemblages in the Thermomediterranean region

Name	Unmodelled (BP)						Modelled (BP)						Indices					
Amodel 115.6																		
Aoverall 114.5"																		
	from	to	%	from	to	%	from	to	%	from	to	%	Acomb	A	L	P	C	
Boundary End Aurignacian	Thermomediterranean Region							33552	33039	68.26895	33751	32648	95.449974					96.4
R_Date Bajondillo_BJ/11_CNA-3878.1.1	33870	33241	68.268949	34216	32960	95.449974	33934	33400	68.268949	34181	33185	95.449974		105.9		95.5	99.2	
R_Date PegodoDiablo_2b_VERA-4047	33981	33259	68.268949	34335	32426	95.449973	34030	33440	68.268949	34268	33187	95.449974		108.7		95.4	99.4	
R_Date Bajondillo_BJ/11_CNA-3817.1	34059	33408	68.268949	34286	33124	95.449974	34092	33530	68.268949	34281	33251	95.449974		106.3		95.4	99.1	
R_Date LaBoja_OH17_VERA-6155HS	34143	33429	68.268949	34385	33065	95.449974	34163	33556	68.268949	34357	33246	95.449974		107.6		95.4	99.2	
R_Date GorhamCave_CON9_OxA-7077	34480	32939	68.268949	35060	31944	95.449973	34402	33422	68.268949	35193	33085	95.449974		113.5		95.2	99	
R_Date CovadelesMalladetes_XVIC_VERA-6427A	34189	33540	68.268949	34355	33179	95.449974	34185	33608	68.26895	34354	33290	95.449974		105.9		95.4	99.2	
R_Date LaBoja_OH17_VERA-5853HS	34230	33542	68.268949	34414	33145	95.449974	34218	33623	68.268949	34402	33284	95.449974		106.6		95.5	99.4	
R_Date CovadelesForadada_V_Beta-103781	34251	33831	68.268949	34419	33530	95.449974	34251	33834	68.268949	34420	33555	95.449974		101.5		95.3	99.4	
R_Date CovadelesMalladetes_XVIC_VERA-6427ABOxSC	34327	33805	68.268949	34497	33392	95.449974	34322	33811	68.268949	34506	33443	95.449974		102.4		95.4	99.3	
R_Date CovadelesMalladetes_XIII_VERA-6509	34353	33824	68.268949	34531	33402	95.449974	34351	33832	68.268949	34539	33463	95.449974		102.5		95.5	99.4	
R_Date GorhamCave_CON9_OxA-7075	35155	33610	68.268949	35927	32290	95.449973	35119	33679	68.26895	35777	33203	95.449974		108.5		95.2	98.8	
R_Date PegodoDiablo_2a_VERA-4049	34576	34076	68.268949	35035	33685	95.449973	34576	34075	68.268949	34936	33684	95.449974		100.8		95.3	99.4	
R_Date LaBoja_OH18_VERA-5854HS	34810	34241	68.268949	35249	34077	95.449974	34814	34242	68.268949	35260	34065	95.449974		100.2		95.3	99.3	
R_Date CovadelesMalladetes_XII_VERA-6508	34775	34271	68.268949	35210	34134	95.449974	34780	34265	68.268949	35227	34122	95.449974		100.1		95.1	99.3	
R_Date GorhamCave_CON9_OxA-7074	35409	34052	68.268949	36195	33257	95.449974	35387	34085	68.268949	36157	33464	95.449974		102.8		95.2	99	
R_Date CovaForadada_IIIc_Beta-37880	34772	34405	68.268949	35148	34316	95.449974	34775	34399	68.268949	35160	34312	95.449974		100.4		95.4	99.6	
R_Date GorhamCave_CON9_OxA-7076	35443	34103	68.268949	36221	33311	95.449974	35428	34124	68.268949	36197	33504	95.449974		102.3		95.2	98.9	

R_Date PegodoDiablo_2a_VERA-4050	35062	34405	68.26895	35336	34208	95.449974	35072	34400	68.268949	35349	34191	95.449974	100.3	95.2	99.2
R_Date CovaForadada_IIIc_MAMS-33909	35310	34874	68.268949	35444	34691	95.449974	35312	34869	68.26895	35454	34680	95.449974	100.3	95.3	99.3
R_Date CovaForadada_IIIc_Beta-414540	35326	34858	68.268949	35498	34661	95.449974	35331	34851	68.268949	35508	34650	95.449974	100.3	95.2	99.3
R_Date LaBoja_OH17_VERA-6156	35615	34805	68.268949	36057	34618	95.449974	35614	34805	68.268949	36067	34610	95.449974	100.3	95.2	99.2
R_Date Bajondillo_BJ/11_CNA-3883 1.1	35983	35394	68.268949	36271	35123	95.449974	35987	35392	68.268949	36280	35100	95.449974	100.3	95.4	99.4
R_Date CovaForadada_IIIc_Beta-37881	36265	35850	68.268949	36390	35547	95.449974	36266	35842	68.268949	36406	35531	95.449974	100.1	95.2	99.2
R_Date CovadelesMalladetes_XIII_VERA-6512	36630	35845	68.268949	36981	35464	95.449974	36635	35839	68.268949	36995	35451	95.449974	100.2	95.2	99.1
R_Date CovaForadada_IIIc_OxA-34251	37056	35790	68.268949	38195	35284	95.449974	37060	35773	68.268949	38210	35274	95.449974	100.1	95.1	98.8
R_Date LaBoja_OH19_VERA-5854	36914	36080	68.268949	37430	35550	95.449974	36922	36079	68.268949	37462	35525	95.449973	100.1	95.1	98.9
R_Date CovadelesMalladetes_XIII_VERA-6510ABOxSC	36837	36122	68.268949	37255	35631	95.449974	36837	36119	68.268949	37300	35624	95.449973	100	95.1	99
R_Date CovadelesMalladetes_XIII_VERA-VERA-6510	36876	36147	68.268949	37371	35680	95.449974	36891	36147	68.268949	37393	35673	95.449974	100.2	95.1	99
R_Date CovadelesMalladetes_XIII_VERA-VERA-6511	36901	36176	68.268949	37445	35755	95.449974	36906	36171	68.268949	37463	35737	95.449974	100	95.1	99.1
R_Date LaBoja_OH19_VERA-6158HS	37035	36163	68.268949	37715	35567	95.449974	37048	36161	68.268949	37739	35553	95.449974	100.1	95.1	98.9
R_Date CovadelesMalladetes_XIVA_VERA-6511ABOxSC	37099	36305	68.268949	38017	36002	95.449974	37120	36301	68.26895	37795	35990	95.449974	100.2	95.1	98.9
R_Date CovadelesMalladetes_XIVA_VERA-6513	37362	36421	68.268949	38306	36152	95.449974	37382	36415	68.268949	38331	36145	95.449974	100.1	95.1	99.1
R_Date Bajondillo_BJ/11_CNA-3876.1.1	37647	36614	68.268949	38608	36329	95.449974	37651	36613	68.268949	38628	36319	95.449974	100.1	95.1	99
R_Date CovadelesMalladetes_XIVA_VERA-6513ABOxSC	38043	36691	68.268949	38884	36409	95.449974	38073	36695	68.26895	38894	36398	95.449974	100.1	95.1	98.9
R_Date CovaForadada_IIIc_OxA-34233	38502	37154	68.268949	39160	36885	95.449974	38541	37159	68.268949	39173	36860	95.449974	100.1	95.1	98.8
R_Date LaBoja_OH19_VERA-6157AboxSc	38634	37122	68.268949	39263	36697	95.449974	38791	37120	68.268949	39276	36667	95.449974	100.2	95.2	98.9
R_Date LaBoja_OH19_VERA-6157	38907	37402	68.268949	39369	36816	95.449974	38926	37373	68.26895	39391	36800	95.449974	100.1	95.1	98.8
R_Date CovadelesMalladetes_XIVA_VERA-6514	38899	37444	68.268949	39330	36889	95.449974	38947	37447	68.26895	39348	36859	95.449974	100	95.1	98.7
R_Date CovadelesMalladetes_XIVA_VERA-6514AboxSc	38903	37567	68.268949	39279	37043	95.449974	38932	37549	68.268949	39286	37030	95.449974	100.1	95.1	99

R_Date PegodoDiablo_II/III_OxA-15499	41185	39138	68.268949	41895	37640	95.449974	41192	39135	68.268949	41897	37652	95.449974		100.4	95.2	98.8	
R_Date PegodoDiablo_II/III_OxA-X-2272-25	40939	39523	68.268949	41810	38656	95.449974	40957	39514	68.268949	41807	38652	95.449974		100.2	95.2	99.1	
R_Date Bajondillo_BJ/13_CNA3873.1.1	41950	41576	68.268949	42056	41385	95.449974	41951	41577	68.268949	42061	41376	95.449974		100.6	95.5	99.5	
R_Date Bajondillo_BJ/13_CNA-3216.3.1	41951	41571	68.268949	42062	41374	95.449974	41951	41568	68.268949	42069	41368	95.449974		100.6	95.6	99.5	
R_Date Bajondillo_BJ/13_CNA3213.3.2	42893	40127	68.268949	45584	38573	95.449974	42326	40568	68.268949	42658	39118	95.449974		118.4	95.2	98.6	
R_Date Bajondillo_BJ/13_CNA3882.1.2	42327	41723	68.268949	42506	41291	95.449974	42286	41719	68.268949	42415	41297	95.449974		103.8	95.5	99.5	
R_Date Bajondillo_BJ/13_CNA-3218.1.2	42418	42230	68.268949	42517	42127	95.449974	42380	42185	68.268949	42484	42060	95.449974		93.8	94.5	99.2	
Phase Aurignacian																	
Boundary Start Aurignacian							42588	42234	68.268949	42969	42095	95.449974				96.9	
Sequence																	
N(0	2)	-2.06	2.06	68.268949	-4	4	95.449974										100
Outlier_Model SSimple																100	
U(0	4)	3.99E-17	4	68.268949	3.99E-17	4	95.449974	5.38E-17	2.332	68.268949	5.38E-17	3.416	95.449974	100			99.5
T(5)	-1.14	1.14	68.268949	-2.65	2.65	95.449974										99.8	
Outlier_Model General							-116	116	68.268949	-1117	1284	95.449974				100	
Curve IntCal20																	

b. **Excluding dates performed on shells**

```
Options()
 {
  Curve("IntCal20","intcal20.14c");
  BCAD=FALSE;
  SD1=TRUE;
  SD2=TRUE;
  ConvergenceData=TRUE;
  kIterations=300;
 };
Plot()
{
  Outlier_Model("General",T(5),U(0,4),"t");
Outlier_Model("SSimple",N(0,2),0,"s");
  Sequence()
  {
    Boundary("Start Aurignacian");
    Phase("Aurignacian")
{
      R_Date("Bajondillo_CNA3213.3.2", 37005, 1790)
      {
        Outlier("General", 0.05);
      };
      R_Date("Bajondillo_CNA-3216.3.1", 36890, 210)
      {
        Outlier("General", 0.05);
      };
R_Date("Bajondillo_CNA3873.1.1",36890, 200)
      {
        Outlier("General", 0.05);
      };

R_Date("PegodoDiablo_OxA-X-2272-25",35050, 750)
      {
        Outlier("General", 0.05);
      };
R_Date("PegodoDiablo_OxA-15499",34900, 1000)
      {
        Outlier("General", 0.05);
      };
R_Date("CovadelesMalladetes_VERA-6514AboxSc",33370, 390)
      {
        Outlier("General", 0.05);
      };
R_Date("CovadelesMalladetes_VERA-6514",33300, 440)
      {
        Outlier("General", 0.05);
      };
R_Date("LaBoja_VERA-6157",33290, 466)
      {
        Outlier("General", 0.05);
      };
R_Date("LaBoja_VERA-6157AboxSc",33179, 455)
      {
        Outlier("General", 0.05);
      };
```

```
R_Date("CovaForadada_OxA-34233 ",33170, 370)
   {
    Outlier("General", 0.05);
   };
R_Date("CovadelesMalladetes_VERA-6513ABOxSC",32860, 380)
   {
    Outlier("General", 0.05);
   };
R_Date("CovadelesMalladetes_VERA-6513",32560, 370)
   {
    Outlier("General", 0.05);
   };
R_Date("CovadelesMalladetes_VERA-6511ABOxSC",32400, 360)
   {
    Outlier("General", 0.05);
   };
R_Date("LaBoja_VERA-6158HS",32231, 417)
   {
    Outlier("General", 0.05);
   };
R_Date("CovadelesMalladetes_VERA-VERA-6511",32160, 360)
   {
    Outlier("General", 0.05);
   };
R_Date("CovadelesMalladetes_VERA-VERA-6510",32120, 360)
   {
    Outlier("General", 0.05);
   };
R_Date("CovadelesMalladetes_VERA-6510ABOxSC",32080, 350)
   {
    Outlier("General", 0.05);
   };
R_Date("LaBoja_VERA-5854",32080, 400)
   {
    Outlier("General", 0.05);
   };
R_Date("CovaForadada_OxA-34251",32050, 550)
   {
    Outlier("General", 0.05);
   };
R_Date("CovadelesMalladetes_VERA-6512",31880, 350)
   {
    Outlier("General", 0.05);
   };
R_Date("CovaForadada_Beta-37881",31690, 180)
   {
    Outlier("General", 0.05);
   };
R_Date("LaBoja_VERA-6156",30918, 343)
   {
    Outlier("General", 0.05);
   };
R_Date("CovaForadada_Beta-414540",30770, 180)
   {
    Outlier("General", 0.05);
   };
R_Date("CovaForadada_MAMS-33909 ",30760, 150)
   {
    Outlier("General", 0.05);
```

```
    };
R_Date("PegodoDiablo_VERA-4050",30260, 320)
    {
     Outlier("General", 0.05);
    };
R_Date("GorhamCave_OxA-7076",30250, 700)
    {
     Outlier("General", 0.05);
    };
R_Date("CovaForadada_Beta-37880",30220, 180)
    {
     Outlier("General", 0.05);
    };
R_Date("GorhamCave_OxA-7074",30200, 700)
    {
     Outlier("General", 0.05);
    };
R_Date("CovadelesMalladetes_VERA-6508",30100, 280)
    {
     Outlier("General", 0.05);
    };
R_Date("LaBoja_VERA-5854HS",30090, 310)
    {
     Outlier("General", 0.05);
    };
R_Date("PegodoDiablo_VERA-4049",29810, 300)
    {
     Outlier("General", 0.05);
    };
R_Date("GorhamCave_OxA-7075",29800, 700)
    {
     Outlier("General", 0.05);
    };
R_Date("CovadelesMalladetes_VERA-6509", 29520, 270)
    {
     Outlier("General", 0.05);
    };
R_Date("CovadelesMalladetes_VERA-6427ABOxSC", 29490, 260)
    {
     Outlier("General", 0.05);
    };
R_Date("CovadelesForadada_Beta-103781", 29440, 190)
    {
     Outlier("General", 0.05);
    };
R_Date("LaBoja_VERA-5853HS",29300, 290)
    {
     Outlier("General", 0.05);
    };
R_Date("CovadelesMalladetes_VERA-6427A", 29270, 260)
    {
     Outlier("General", 0.05);
    };
R_Date("GorhamCave_OxA-7077",29250, 650)
    {
     Outlier("General", 0.05);
    };
R_Date("LaBoja_VERA-6155HS",29230, 287)
    {
```

```
      Outlier("General", 0.05);
     };
 R_Date("PegodoDiablo_VERA-4047",29090, 270)
     {
      Outlier("General", 0.05);
     };
   };
   Boundary("End Aurignacian ");
  };
 };
};
```

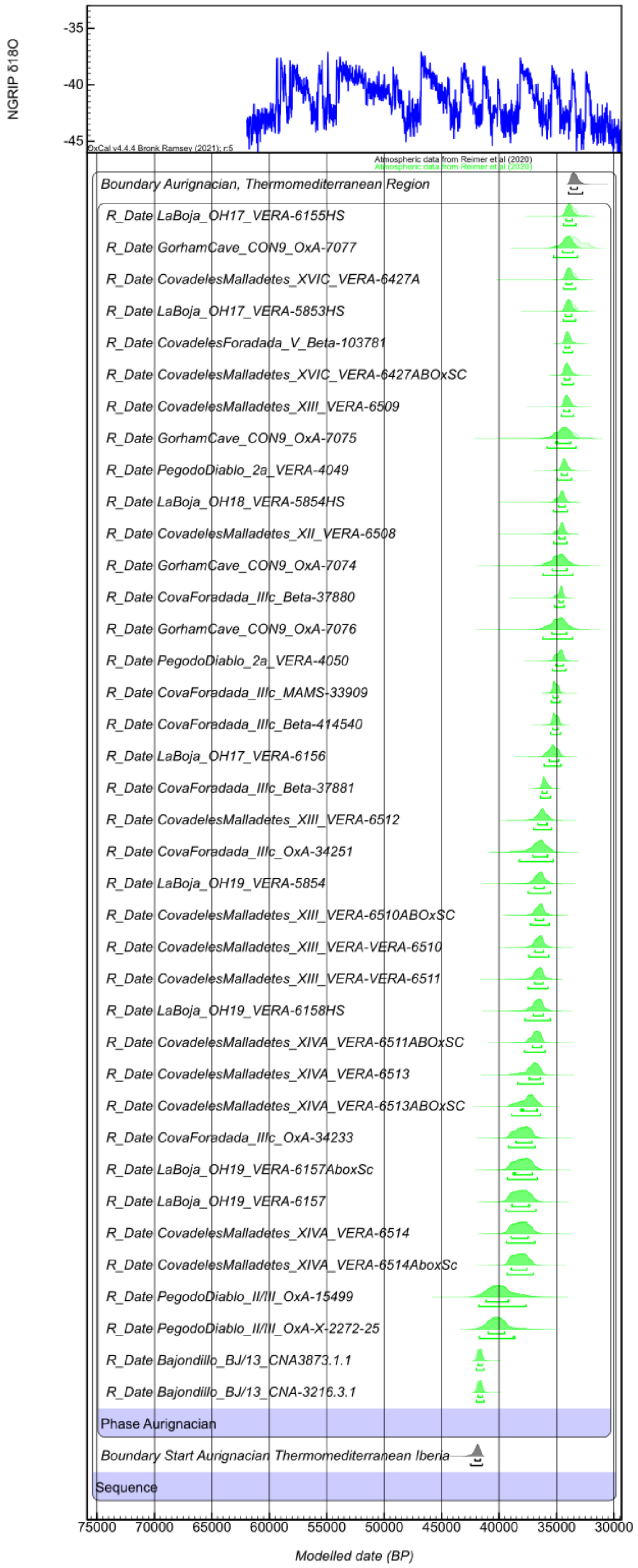

Figure 24 Plot of dated radiocarbon dates from Aurignacian assemblages in the Thermomediterranean region after excluding the radiocarbon determinations obtained from shell remains.

Name	Unmodelled (BP)						Modelled (BP)						Indices					
Amodel 112.8																		
Aoverall 111"																		
	from	to	%	from	to	%	from	to	%	from	to	%	Acomb	A	L	P	C	
Boundary End Aurignacian	Thermomediterranean Region							33766	33199	68.268949	33950	32740	95.449974					96.5
R_Date LaBoja_OH17_VERA-6155HS	34143	33429	68.268949	34385	33065	95.449974	34189	33651	68.268949	34386	33313	95.449974		108.9		95.3	99.2	
R_Date GorhamCave_CON9_OxA-7077	34480	32939	68.268949	35060	31944	95.449973	34468	33557	68.268949	35239	33166	95.449974		111.7		95.2	98.7	
R_Date CovadelesMalladetes_XVIC_VERA-6427A	34189	33540	68.268949	34355	33179	95.449974	34191	33684	68.268949	34381	33351	95.449974		108.5		95.3	98.9	
R_Date LaBoja_OH17_VERA-5853HS	34230	33542	68.268949	34414	33145	95.449974	34226	33693	68.268949	34425	33347	95.449974		109.3		95.4	99.2	
R_Date CovadelesForadada_V_Beta-103781	34251	33831	68.268949	34419	33530	95.449974	34251	33852	68.268949	34420	33612	95.449974		104.1		95.6	99.6	
R_Date CovadelesMalladetes_XVIC_VERA-6427ABOxSC	34327	33805	68.268949	34497	33392	95.449974	34318	33842	68.268949	34517	33526	95.449974		105.6		95.6	99.4	
R_Date CovadelesMalladetes_XIII_VERA-6509	34353	33824	68.268949	34531	33402	95.449974	34344	33848	68.268949	34555	33532	95.449974		105		95.4	99.4	
R_Date GorhamCave_CON9_OxA-7075	35155	33610	68.268949	35927	32290	95.449973	35087	33762	68.268949	35829	33306	95.449974		110.2		95.3	98.9	
R_Date PegodoDiablo_2a_VERA-4049	34576	34076	68.268949	35035	33685	95.449973	34574	34076	68.268949	34904	33705	95.449974		101.6		95.4	99.4	
R_Date LaBoja_OH18_VERA-5854HS	34810	34241	68.268949	35249	34077	95.449974	34813	34241	68.268949	35254	34062	95.449974		100.6		95.4	99.2	
R_Date CovadelesMalladetes_XII_VERA-6508	34775	34271	68.268949	35210	34134	95.449974	34777	34265	68.268949	35228	34120	95.449974		100.1		95.1	99.3	
R_Date GorhamCave_CON9_OxA-7074	35409	34052	68.268949	36195	33257	95.449974	35372	34088	68.268949	36179	33581	95.449974		104.1		95.3	98.8	
R_Date CovaForadada_IIIc_Beta-37880	34772	34405	68.268949	35148	34316	95.449974	34777	34404	68.268949	35159	34309	95.449974		100.4		95.4	99.4	
R_Date GorhamCave_CON9_OxA-7076	35443	34103	68.268949	36221	33311	95.449974	35403	34130	68.268949	36199	33607	95.449974		103.4		95.2	99	
R_Date PegodoDiablo_2a_VERA-4050	35062	34405	68.26895	35336	34208	95.449974	35058	34404	68.26895	35349	34195	95.449974		100.4		95.4	99.4	
R_Date CovaForadada_IIIc_MAMS-33909	35310	34874	68.268949	35444	34691	95.449974	35309	34868	68.268949	35455	34685	95.449974		100.3		95.4	99.5	
R_Date CovaForadada_IIIc_Beta-414540	35326	34858	68.268949	35498	34661	95.449974	35327	34854	68.268949	35511	34651	95.449974		100.3		95.4	99.5	

R_Date LaBoja_OH17_VERA-6156	35615	34805	68.268949	36057	34618	95.449974	35620	34803	68.268949	36074	34610	95.449974	100.3	95.3	99.2
R_Date CovaForadada_IIIc_Beta-37881	36265	35850	68.268949	36390	35547	95.449974	36265	35843	68.268949	36399	35533	95.449974	100.3	95.5	99.5
R_Date CovadelesMalladetes_XIII_VERA-6512	36630	35845	68.268949	36981	35464	95.449974	36637	35819	68.268949	36999	35445	95.449974	100.1	95.2	99.2
R_Date CovaForadada_IIIc_OxA-34251	37056	35790	68.268949	38195	35284	95.449974	37068	35763	68.268949	38239	35275	95.449974	100.1	95.2	99
R_Date LaBoja_OH19_VERA-5854	36914	36080	68.268949	37430	35550	95.449974	36917	36073	68.268949	37451	35529	95.449974	100.1	95.1	99
R_Date CovadelesMalladetes_XIII_VERA-6510ABOxSC	36837	36122	68.268949	37255	35631	95.449974	36842	36120	68.268949	37284	35617	95.449974	100.3	95.2	99.3
R_Date CovadelesMalladetes_XIII_VERA-VERA-6510	36876	36147	68.268949	37371	35680	95.449974	36884	36140	68.268949	37405	35668	95.449974	100.1	95.2	99.1
R_Date CovadelesMalladetes_XIII_VERA-VERA-6511	36901	36176	68.268949	37445	35755	95.449974	36904	36174	68.268949	37468	35736	95.449974	100.1	95.2	99.1
R_Date LaBoja_OH19_VERA-6158HS	37035	36163	68.268949	37715	35567	95.449974	37039	36157	68.268949	37749	35538	95.449974	100.2	95.2	98.9
R_Date CovadelesMalladetes_XIVA_VERA-6511ABOxSC	37099	36305	68.268949	38017	36002	95.449974	37104	36299	68.268949	37783	35994	95.449974	100.2	95.2	99
R_Date CovadelesMalladetes_XIVA_VERA-6513	37362	36421	68.268949	38306	36152	95.449974	37373	36415	68.268949	38354	36145	95.449974	100.2	95.2	99
R_Date CovadelesMalladetes_XIVA_VERA-6513ABOxSC	38043	36691	68.268949	38884	36409	95.449974	38117	36680	68.268948	38901	36404	95.449974	100.2	95.2	99.1
R_Date CovaForadada_IIIc_OxA-34233	38502	37154	68.268949	39160	36885	95.449974	38544	37160	68.268949	39172	36861	95.449974	100.2	95.2	99
R_Date LaBoja_OH19_VERA-6157AboxSc	38634	37122	68.268949	39263	36697	95.449974	38746	37128	68.268949	39274	36682	95.449974	100.2	95.1	99
R_Date LaBoja_OH19_VERA-6157	38907	37402	68.268949	39369	36816	95.449974	38907	37343	68.268949	39378	36798	95.449974	100.2	95.2	98.9
R_Date CovadelesMalladetes_XIVA_VERA-6514	38899	37444	68.268949	39330	36889	95.449974	38927	37447	68.268949	39347	36876	95.449974	100.2	95.2	98.9
R_Date CovadelesMalladetes_XIVA_VERA-6514AboxSc	38903	37567	68.268949	39279	37043	95.449974	38946	37565	68.26895	39296	37031	95.449974	100.2	95.1	99.1
R_Date PegodoDiablo_II/III_OxA-15499	41185	39138	68.268949	41895	37640	95.449974	41151	39155	68.268949	41741	37675	95.449974	101.9	95.3	99
R_Date PegodoDiablo_II/III_OxA-X-2272-25	40939	39523	68.268949	41810	38656	95.449974	40932	39516	68.268949	41713	38633	95.449974	101.1	95.2	99.1
R_Date Bajondillo_BJ/13_CNA3873.1.1	41950	41576	68.268949	42056	41385	95.449974	41828	41461	68.268949	41987	41322	95.449974	93.6	95.3	99
R_Date Bajondillo_BJ/13_CNA-3216.3.1	41951	41571	68.268949	42062	41374	95.449974	41827	41457	68.268949	41988	41313	95.449974	94.1	95.3	99.1

Phase Aurignacian

Boundary Start Aurignacian							42099	41635	68.2689 49	42474	41453	95.4499 74			97. 2	
Sequence																
N(0	2)	-2.06	2.06	68.2689 49	-4	4	95.4499 74									10 0
Outlier_Model SSimple															10 0	
U(0	4)	3.99E- 17	4	68.2689 49	3.99E- 17	4	95.4499 74	5.38E- 17	2.3	68.2689 49	5.38E- 17	3.508	95.4499 74	10 0		99. 7
T(5)	-1.14	1.14	68.2689 49	-2.65	2.65	95.4499 74									99. 9	
Outlier_Model General							-124	126	68.2689 49	-1229	1473	95.4499 73			10 0	
Curve IntCal20																

c. Without youngest and oldest dates

```
Options()
 {
  Curve("IntCal20","intcal20.14c");
  BCAD=FALSE;
  SD1=TRUE;
  SD2=TRUE;
  ConvergenceData=TRUE;
  kIterations=300;
 };
Plot()
{
  Outlier_Model("General",T(5),U(0,4),"t");
Outlier_Model("SSimple",N(0,2),0,"s");
  Sequence()
  {
    Boundary("Start Aurignacian");
    Phase("Aurignacian")
{
      R_Date("Bajondillo_CNA-3216.3.1", 36890, 210)
      {
        Outlier("General", 0.05);
      };
R_Date("Bajondillo_CNA3873.1.1",36890, 200)
      {
        Outlier("General", 0.05);
      };

R_Date("PegodoDiablo_OxA-X-2272-25",35050, 750)
      {
        Outlier("General", 0.05);
      };
R_Date("PegodoDiablo_OxA-15499",34900, 1000)
      {
        Outlier("General", 0.05);
      };
R_Date("CovadelesMalladetes_VERA-6514AboxSc",33370, 390)
      {
        Outlier("General", 0.05);
      };
R_Date("CovadelesMalladetes_VERA-6514",33300, 440)
      {
        Outlier("General", 0.05);
      };
R_Date("LaBoja_VERA-6157",33290, 466)
      {
        Outlier("General", 0.05);
      };
R_Date("LaBoja_VERA-6157AboxSc",33179, 455)
      {
        Outlier("General", 0.05);
      };
R_Date("CovaForadada_OxA-34233 ",33170, 370)
      {
        Outlier("General", 0.05);
      };
R_Date("CovadelesMalladetes_VERA-6513ABOxSC",32860, 380)
```

```
    {
     Outlier("General", 0.05);
    };
R_Date("CovadelesMalladetes_VERA-6513",32560, 370)
    {
     Outlier("General", 0.05);
    };
R_Date("CovadelesMalladetes_VERA-6511ABOxSC",32400, 360)
    {
     Outlier("General", 0.05);
    };
R_Date("LaBoja_VERA-6158HS",32231, 417)
    {
     Outlier("General", 0.05);
    };
R_Date("CovadelesMalladetes_VERA-VERA-6511",32160, 360)
    {
     Outlier("General", 0.05);
    };
R_Date("CovadelesMalladetes_VERA-VERA-6510",32120, 360)
    {
     Outlier("General", 0.05);
    };
R_Date("CovadelesMalladetes_VERA-6510ABOxSC",32080, 350)
    {
     Outlier("General", 0.05);
    };
R_Date("LaBoja_VERA-5854",32080, 400)
    {
     Outlier("General", 0.05);
    };
R_Date("CovaForadada_OxA-34251",32050, 550)
    {
     Outlier("General", 0.05);
    };
R_Date("CovadelesMalladetes_VERA-6512",31880, 350)
    {
     Outlier("General", 0.05);
    };
R_Date("CovaForadada_Beta-37881",31690, 180)
    {
     Outlier("General", 0.05);
    };
R_Date("LaBoja_VERA-6156",30918, 343)
    {
     Outlier("General", 0.05);
    };
R_Date("CovaForadada_Beta-414540",30770, 180)
    {
     Outlier("General", 0.05);
    };
R_Date("CovaForadada_MAMS-33909 ",30760, 150)
    {
     Outlier("General", 0.05);
    };
R_Date("PegodoDiablo_VERA-4050",30260, 320)
    {
     Outlier("General", 0.05);
    };
```

```
R_Date("GorhamCave_OxA-7076",30250, 700)
    {
     Outlier("General", 0.05);
    };
R_Date("CovaForadada_Beta-37880",30220, 180)
    {
     Outlier("General", 0.05);
    };
R_Date("GorhamCave_OxA-7074",30200, 700)
    {
     Outlier("General", 0.05);
    };
R_Date("CovadelesMalladetes_VERA-6508",30100, 280)
    {
     Outlier("General", 0.05);
    };
R_Date("LaBoja_VERA-5854HS",30090, 310)
    {
     Outlier("General", 0.05);
    };
R_Date("PegodoDiablo_VERA-4049",29810, 300)
    {
     Outlier("General", 0.05);
    };
R_Date("GorhamCave_OxA-7075",29800, 700)
    {
     Outlier("General", 0.05);
    };
R_Date("CovadelesMalladetes_VERA-6509", 29520, 270)
    {
     Outlier("General", 0.05);
    };
R_Date("CovadelesMalladetes_VERA-6427ABOxSC", 29490, 260)
    {
     Outlier("General", 0.05);
    };
R_Date("CovadelesForadada_Beta-103781", 29440, 190)
    {
     Outlier("General", 0.05);
    };
R_Date("LaBoja_VERA-5853HS",29300, 290)
    {
     Outlier("General", 0.05);
    };
R_Date("CovadelesMalladetes_VERA-6427A", 29270, 260)
    {
     Outlier("General", 0.05);
    };
R_Date("GorhamCave_OxA-7077",29250, 650)
    {
     Outlier("General", 0.05);
    };
R_Date("LaBoja_VERA-6155HS",29230, 287)
    {
     Outlier("General", 0.05);
    };
   };
  Boundary("End Aurignacian ");
 };
```

```
 };
};
```

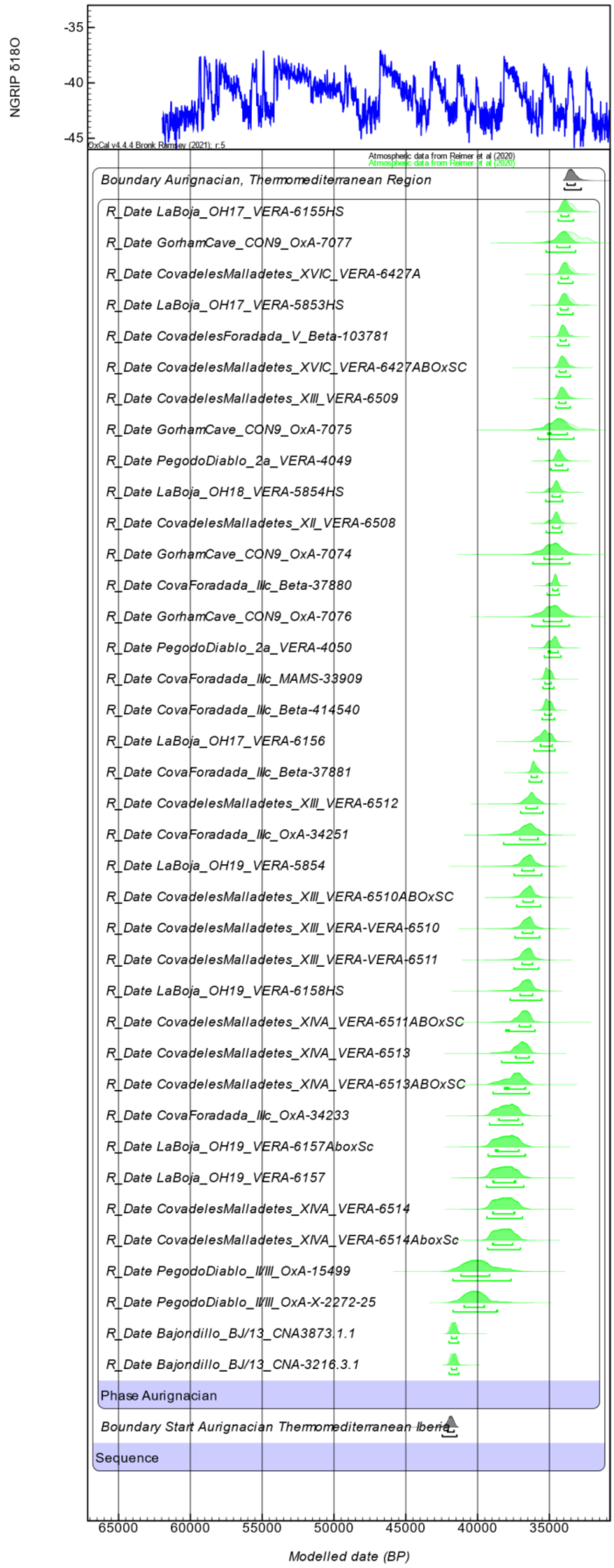

NGRIP δ18O
-35
-40
-45
OxCal v4.4.4 Bronk Ramsey (2021); r:5
Atmospheric data from Reimer et al (2020)
Boundary Aurignacian, Thermomediterranean Region
R_Date LaBoja_OH17_VERA-6155HS
R_Date GorhamCave_CON9_OxA-7077
R_Date CovadelesMalladetes_XVIC_VERA-6427A
R_Date LaBoja_OH17_VERA-5853HS
R_Date CovadelesForadada_V_Beta-103781
R_Date CovadelesMalladetes_XVIC_VERA-6427ABOxSC
R_Date CovadelesMalladetes_XIII_VERA-6509
R_Date GorhamCave_CON9_OxA-7075
R_Date PegodoDiablo_2a_VERA-4049
R_Date LaBoja_OH18_VERA-5854HS
R_Date CovadelesMalladetes_XII_VERA-6508
R_Date GorhamCave_CON9_OxA-7074
R_Date CovaForadada_IIIc_Beta-37880
R_Date GorhamCave_CON9_OxA-7076
R_Date PegodoDiablo_2a_VERA-4050
R_Date CovaForadada_IIIc_MAMS-33909
R_Date CovaForadada_IIIc_Beta-414540
R_Date LaBoja_OH17_VERA-6156
R_Date CovaForadada_IIIc_Beta-37881
R_Date CovadelesMalladetes_XIII_VERA-6512
R_Date CovaForadada_IIIc_OxA-34251
R_Date LaBoja_OH19_VERA-5854
R_Date CovadelesMalladetes_XIII_VERA-6510ABOxSC
R_Date CovadelesMalladetes_XIII_VERA-VERA-6510
R_Date CovadelesMalladetes_XIII_VERA-VERA-6511
R_Date LaBoja_OH19_VERA-6158HS
R_Date CovadelesMalladetes_XIVA_VERA-6511ABOxSC
R_Date CovadelesMalladetes_XIVA_VERA-6513
R_Date CovadelesMalladetes_XIVA_VERA-6513ABOxSC
R_Date CovaForadada_IIIc_OxA-34233
R_Date LaBoja_OH19_VERA-6157AboxSc
R_Date LaBoja_OH19_VERA-6157
R_Date CovadelesMalladetes_XIVA_VERA-6514
R_Date CovadelesMalladetes_XIVA_VERA-6514AboxSc
R_Date PegodoDiablo_II/III_OxA-15499
R_Date PegodoDiablo_II/III_OxA-X-2272-25
R_Date Bajondillo_BJ/13_CNA3873.1.1
R_Date Bajondillo_BJ/13_CNA-3216.3.1
Phase Aurignacian
Boundary Start Aurignacian Thermomediterranean Iberia
Sequence
65000
60000
55000
50000
45000
40000
35000
Modelled date (BP)

Figure 25 Plot of dated radiocarbon dates from Aurignacian assemblages in the Thermomediterranean region after excluding the oldest and youngest dates.

Name	Unmodelled (BP)						Modelled (BP)						Indices					
Amodel 112.4																		
Aoverall 111.2"																		
	from	to	%	from	to	%	from	to	%	from	to	%	Acomb	A	L	P	C	
Boundary End Aurignacian	Thermomediterranean Region							33773	33208	68.268949	33956	32783	95.449974					96
R_Date LaBoja_OH17_VERA-6155HS	34143	33429	68.268949	34385	33065	95.449974	34191	33658	68.268949	34382	33317	95.449974		109.1		95.4	99.1	
R_Date GorhamCave_CON9_OxA-7077	34480	32939	68.268949	35060	31944	95.449973	34476	33572	68.268949	35233	33178	95.449974		111.6		95.2	98.9	
R_Date CovadelesMalladetes_XVIC_VERA-6427A	34189	33540	68.268949	34355	33179	95.449974	34191	33688	68.268949	34376	33362	95.449974		108.9		95.5	99.1	
R_Date LaBoja_OH17_VERA-5853HS	34230	33542	68.268949	34414	33145	95.449974	34227	33697	68.268949	34426	33355	95.449974		109.5		95.5	99.3	
R_Date CovadelesForadada_V_Beta-103781	34251	33831	68.268949	34419	33530	95.449974	34247	33850	68.268949	34420	33619	95.449974		104.1		95.5	99.6	
R_Date CovadelesMalladetes_XVIC_VERA-6427ABOxSC	34327	33805	68.268949	34497	33392	95.449974	34321	33847	68.268949	34520	33532	95.449974		105.8		95.5	99.4	
R_Date CovadelesMalladetes_XIII_VERA-6509	34353	33824	68.268949	34531	33402	95.449974	34345	33858	68.268949	34554	33545	95.449974		105.4		95.5	99.4	
R_Date GorhamCave_CON9_OxA-7075	35155	33610	68.268949	35927	32290	95.449973	35122	33749	68.268949	35801	33303	95.449974		110.4		95.3	99	
R_Date PegodoDiablo_2a_VERA-4049	34576	34076	68.268949	35035	33685	95.449973	34574	34077	68.268949	34905	33708	95.449973		101.7		95.5	99.4	
R_Date LaBoja_OH18_VERA-5854HS	34810	34241	68.268949	35249	34077	95.449974	34809	34240	68.268949	35258	34067	95.449974		100.5		95.4	99.4	
R_Date CovadelesMalladetes_XII_VERA-6508	34775	34271	68.268949	35210	34134	95.449974	34775	34265	68.268949	35216	34124	95.449974		100.5		95.5	99.4	
R_Date GorhamCave_CON9_OxA-7074	35409	34052	68.268949	36195	33257	95.449974	35372	34095	68.268949	36156	33585	95.449974		104.1		95.2	98.9	
R_Date CovaForadada_IIIc_Beta-37880	34772	34405	68.268949	35148	34316	95.449974	34775	34400	68.268949	35157	34311	95.449974		100.4		95.5	99.6	
R_Date GorhamCave_CON9_OxA-7076	35443	34103	68.268949	36221	33311	95.449974	35422	34136	68.268949	36201	33617	95.449974		103.7		95.3	99	
R_Date PegodoDiablo_2a_VERA-4050	35062	34405	68.26895	35336	34208	95.449974	35080	34402	68.268949	35350	34196	95.449974		100.4		95.4	99.2	
R_Date CovaForadada_IIIc_MAMS-33909	35310	34874	68.268949	35444	34691	95.449974	35311	34875	68.268949	35455	34685	95.449974		100.4		95.4	99.4	
R_Date CovaForadada_IIIc_Beta-414540	35326	34858	68.268949	35498	34661	95.449974	35332	34860	68.268949	35507	34651	95.449974		100.4		95.5	99.5	

R_Date LaBoja_OH17_VERA-6156	35615	34805	68.268949	36057	34618	95.449974	35628	34802	68.26895	36070	34610	95.449974	100.3	95.3	99.2
R_Date CovaForadada_IIIc_Beta-37881	36265	35850	68.268949	36390	35547	95.449974	36269	35844	68.268949	36403	35528	95.449974	100.2	95.2	99.4
R_Date CovadelesMalladetes_XIII_VERA-6512	36630	35845	68.268949	36981	35464	95.449974	36634	35833	68.268949	36995	35448	95.449974	100.2	95.2	99.1
R_Date CovaForadada_IIIc_OxA-34251	37056	35790	68.268949	38195	35284	95.449974	37062	35772	68.26895	38189	35275	95.449974	100.2	95.2	99
R_Date LaBoja_OH19_VERA-5854	36914	36080	68.268949	37430	35550	95.449974	36916	36077	68.268949	37459	35534	95.449974	100.2	95.1	99
R_Date CovadelesMalladetes_XIII_VERA-6510ABOxSC	36837	36122	68.268949	37255	35631	95.449974	36839	36125	68.268949	37274	35616	95.449974	100.2	95.2	99.2
R_Date CovadelesMalladetes_XIII_VERA-VERA-6510	36876	36147	68.268949	37371	35680	95.449974	36882	36146	68.268949	37400	35671	95.449974	100.2	95.2	99.1
R_Date CovadelesMalladetes_XIII_VERA-VERA-6511	36901	36176	68.268949	37445	35755	95.449974	36903	36172	68.268949	37458	35738	95.449974	100.2	95.2	99
R_Date LaBoja_OH19_VERA-6158HS	37035	36163	68.268949	37715	35567	95.449974	37042	36160	68.268949	37742	35535	95.449974	100.2	95.2	98.7
R_Date CovadelesMalladetes_XIVA_VERA-6511ABOxSC	37099	36305	68.268949	38017	36002	95.449974	37104	36300	68.268949	38036	36001	95.449973	100.1	95.1	98.9
R_Date CovadelesMalladetes_XIVA_VERA-6513	37362	36421	68.268949	38306	36152	95.449974	37362	36411	68.268949	38327	36144	95.449974	100.1	95.1	98.9
R_Date CovadelesMalladetes_XIVA_VERA-6513ABOxSC	38043	36691	68.268949	38884	36409	95.449974	38128	36672	68.26895	38914	36402	95.449974	100.1	95.1	98.8
R_Date CovaForadada_IIIc_OxA-34233	38502	37154	68.268949	39160	36885	95.449974	38525	37153	68.26895	39168	36858	95.449974	100.1	95.2	99
R_Date LaBoja_OH19_VERA-6157AboxSc	38634	37122	68.268949	39263	36697	95.449974	38753	37122	68.26895	39266	36665	95.449973	100.1	95.1	98.9
R_Date LaBoja_OH19_VERA-6157	38907	37402	68.268949	39369	36816	95.449974	38934	37344	68.268949	39372	36783	95.449974	100.1	95.1	98.9
R_Date CovadelesMalladetes_XIVA_VERA-6514	38899	37444	68.268949	39330	36889	95.449974	38942	37414	68.268949	39352	36873	95.449974	100.1	95.1	98.9
R_Date CovadelesMalladetes_XIVA_VERA-6514AboxSc	38903	37567	68.268949	39279	37043	95.449974	38951	37561	68.268949	39299	37018	95.449974	100.1	95.1	99
R_Date PegodoDiablo_II/III_OxA-15499	41185	39138	68.268949	41895	37640	95.449974	41155	39168	68.268949	41723	37666	95.449974	101.9	95.2	98.8
R_Date PegodoDiablo_II/III_OxA-X-2272-25	40939	39523	68.268949	41810	38656	95.449974	40946	39519	68.268949	41705	38608	95.449973	101	95.2	99
R_Date Bajondillo_BJ/13_CNA3873.1.1	41950	41576	68.268949	42056	41385	95.449974	41818	41457	68.268949	41986	41321	95.449974	93.2	95.2	99.1
R_Date Bajondillo_BJ/13_CNA-3216.3.1	41951	41571	68.268949	42062	41374	95.449974	41818	41448	68.268949	41985	41310	95.449974	93.7	95.3	99.1

Phase Aurignacian

Boundary Start Aurignacian							42101	41640	68.268949	42458	41442	95.449974			96.3	
Sequence																
N(0	2)	-2.06	2.06	68.268949	-4	4	95.449974									100
Outlier_Model SSimple															100	
U(0	4)	3.99E-17	4	68.268949	3.99E-17	4	95.449974	5.38E-17	2.356	68.268949	5.38E-17	3.492	95.449974	100		99.8
T(5)	-1.14	1.14	68.268949	-2.65	2.65	95.449974									99.9	
Outlier_Model General							-119	120	68.268949	-1141	1373	95.449974			100	
Curve IntCal20																

Summary

The end of the Mousterian is 1,005 years younger if the radiocarbon determinations obtained from shell remains are excluded (Table 7). However, once these radiocarbon dates obtained from shells are removed, the chronology for the end of the Mousterian technocomplex remains similar if level I-K from Cueva Antón, or the oldest and youngest dates of the sequence, are removed (Table 7). Regarding the Châtelperronian, it is worth noting that there is only one archaeological site, so this chronology should be considered with caution when considering it the chronology of the Châtelperronian in this region. Regarding Aurignacian, both the start and end boundaries remains similar if the oldest and youngest dates are excluded (Table 7). However, the start of the Aurignacian is 640 years older when the dates obtained from shell remains are removed (Table 7).

Eurosiberian region

**Culture**	**Boundary**	**Model**	**68.2% prob.**		**95.54% prob.**		**A. model**
			From	to	From	to	
Mousterian	End	a. All dates	36.35	35.18	36.65	33.69	107.7
		b. Excluding dates obtained from shells	36.32	34.84	36.62	32.64	105.3
		c. Excluding Cueva Antón	33.37	35.45	37.93	32.44	106.6
		d. Excluding the oldest and youngest dates	36.92	35.17	37.30	32.58	105.5
Châtelperronian*	Start	a. All dates	40.48	39.50	42.76	39.28	129.1
	End	a. All dates	39.83	38.85	40.24	36.37	129.1
Aurignacian	Start	a. All dates	42.58	42.23	42.96	42.09	115.6
		b. Excluding dates performed on shells	42.09	41.63	42.47	41.45	112.8
		c. Excluding the oldest and youngest dates	42.10	41.64	42.45	41.44	112.4
	End	a. All dates	33.55	33.03	33.75	32.64	115.6
		b. Excluding dates performed on shells	33.76	33.19	33.95	32.74	112.8
		c. Excluding the oldest and youngest dates	33.77	33.20	33.95	32.78	112.4

Table 7. Results of the 68.2% and 95.4% PDF rage of the boundaries between each technocomplex. *Only one archaeological site is considered in the Châtelperronian model

REFERENCES

1. Ruiz, M. N. *et al.* Late Neanderthal subsistence strategies and cultural traditions in the northern Iberia Peninsula: Insights from Prado Vargas, Burgos, Spain. *Quat. Sci. Rev.* **254**, 106795 (2021).
2. Lombera-Hermida, A. de *et al.* Between two worlds: Cova Eirós and the Middle-Upper Palaeolithic transition in NW Iberia. *Comptes Rendus Palevol* **20**, 859–886 (2021).
3. Rey-Rodríguez, I. *et al.* Last Neanderthals and first Anatomically Modern Humans in the NW Iberian Peninsula: Climatic and environmental conditions inferred from the Cova Eirós small-vertebrate assemblage during MIS 3. *Quat. Sci. Rev.* **151**, 185–197 (2016).
4. Maíllo Fernández, J. M. El Chatelperroniense del Noroeste de la Península Ibérica. *Férvedes Rev. Investig. ISSN 1134-6787, N°. 5, 2008, págs. 127-136* 127–136 (2008).
5. Maíllo Fernández, J. M. La producción laminar en el Chatelperroniense de Cueva Morín: modalidades, intenciones y objetivos. *Trab. Prehist.* **62**, 47–64 (2005).
6. Maroto, J. *et al.* Current issues in late Middle Palaeolithic chronology: New assessments from Northern Iberia. *Quat. Int.* **247**, 15–25 (2012).
7. Marín-Arroyo, A. B. *et al.* Chronological reassessment of the Middle to Upper Paleolithic transition and Early Upper Paleolithic cultures in Cantabrian Spain. *PLoS One* **13**, e0194708 (2018).
8. Wood, R. E. *et al.* Radiocarbon dating casts doubt on the late chronology of the Middle to Upper Palaeolithic transition in southern Iberia. *Proc. Natl. Acad. Sci. U. S. A.* **110**, 2781–2786 (2013).
9. Jordá Pardo, J. F. Dataciones isotópicas del yacimiento del Pleistoceno superior de Jarama VI (Alto Valle del Jarama, Guadalajara, España) y sus implicaciones cronoestratigráficas. (2001).
10. Kehl, M. *et al.* Late Neanderthals at Jarama VI (central Iberia)? *Quat. Res.* **80**, 218–234 (2013).
11. Sala, N. *et al.* Central Iberia in the middle MIS 3. Paleoecological inferences during the period 34–40 cal kyr BP. *Quat. Sci. Rev.* **228**, 106027 (2020).
12. Mora, R. *et al.* Contextual, technological and chronometric data from Cova Gran: Their contribution to discussion of the Middle-to-Upper Paleolithic transition in northeastern Iberia. *Quat. Int.* **474**, 30–43 (2018).
13. Carrión, J. S. *et al.* The sequence at Carihuela Cave and its potential for research into Neanderthal ecology and the Mousterian in southern Spain. *Quat. Sci. Rev.* **217**, 194–216 (2019).
14. Zilhão, J. The late persistence of the Middle Palaeolithic and Neandertals in Iberia: A review of the evidence for and against the “Ebro Frontier” model. *Quat. Sci. Rev.* **270**, 107098 (2021).
15. Moreno, A. G. *et al.* La secuencia musteriense de la Cueva del Niño (Aýna, Albacete) y el poblamiento neandertal en el sureste de la Península Ibérica. *Trab. Prehist.* **71**, 221–241 (2014).
16. Michel, V., Delanghe-Sabatier, D., Bard, E. & Barroso Ruiz, C. U-series, ESR and 14C studies of the fossil remains from the Mousterian levels of Zafarraya Cave

(Spain): A revised chronology of Neandertal presence. *Quat. Geochronol.* **15**, 20–33 (2013).
17. Higham, T. *et al.* The timing and spatiotemporal patterning of Neanderthal disappearance. *Nat. 2014 5127514* **512**, 306–309 (2014).
18. Walker, M. J., Ortega, J., Parmová, K., López, M. V & Trinkaus, E. Morphology, body proportions, and postcranial hypertrophy of a female Neandertal from the Sima de las Palomas, southeastern Spain. *Proc. Natl. Acad. Sci. U. S. A.* **108**, 10087–91 (2011).
19. Walker, M. J. *et al.* Late Neandertals in Southeastern Iberia: Sima de las Palomas del Cabezo Gordo, Murcia, Spain. *Proc. Natl. Acad. Sci.* **105**, 20631–20636 (2008).
20. Finlayson, C. *et al.* Late survival of Neanderthals at the southernmost extreme of Europe. *Nat. 2006 4437113* **443**, 850–853 (2006).
21. Zilhão, J. & Pettitt, P. On the new dates for Gorham's Cave and the late survival of Iberian Neanderthals. *Before Farming* **2006**, 1–9 (2006).
22. Barton, R. N. E. *et al.* Gibraltar Neanderthals and results of recent excavations in Gorham's, Vanguard and Ibex Caves. *Antiquity* **73**, 13–23 (1999).
23. Goldberg, P. Geoarchaeological investigation of sediments from Gorham's and Vanguard Caves, Gibraltar: Microstratigraphical (soil micromorphological and chemical) signatures. in *Neanderthals on the Edge* (eds. Stringer, C., Finlayson, G. & Barton, N.) 183–200 (Oxbow Books, 2000).
24. Wood, R. E. The contribution of new radiocarbon dating pre-treatment techniques to understanding the Middle to Upper Palaeolithic transition in Iberia. (2011).
25. Kehl, M. *et al.* The rock shelter Abrigo del Molino (Segovia, Spain) and the timing of the late Middle Paleolithic in Central Iberia. *Quat. Res.* **90**, 180–200 (2018).
26. Hoffmann, D. L., Pike, A. W. G., Wainer, K. & Zilhão, J. New U-series results for the speleogenesis and the Palaeolithic archaeology of the Almonda karstic system (Torres Novas, Portugal). *Quat. Int.* **294**, 168–182 (2013).
27. Zilhão, J. *et al.* A revised, Last Interglacial chronology for the Middle Palaeolithic sequence of Gruta da Oliveira (Almonda karst system, Torres Novas, Portugal). *Quat. Sci. Rev.* **258**, 106885 (2021).
28. Delibrias, G., Guillier, M.-T. & Labeyrie, J. Gif Natural Radiocarbon Measurements X. *Radiocarbon* **28**, 9–68 (1986).
29. Zilhao, J. C., Alistair, W.G., P. & Bernhard, W. Gruta Nova da Columbeira (Bombarral , Portugal) : Site stratigraphy , age of the Mousterian sequence , and implications for the timing of Neanderthal extinction in Iberia. *Quartär Int. Yearb. Ice Age Stone Age Res.* **58**, 93–112 (2012).
30. Carvalho, M., Peireira, T. & Manso, C. Rabbit exploitation in the Middle Paleolithic at Gruta Nova da Columbeira, Portugal. *J. Archaeol. Sci. Reports* **21**, 821–832 (2018).
31. Jennings, R. P. *et al.* New dates and palaeoenvironmental evidence for the Middle to Upper Palaeolithic occupation of Higueral de Valleja Cave, southern Spain. *Quat. Sci. Rev.* **28**, 830–839 (2009).
