# Supplementary material for: Ecosystem productivity affected the spatiotemporal disappearance of Neanderthals in Iberia
Source: Nat Ecol Evol. 2022 Sep 29;6(11):1644–57. doi: 10.1038/s41559-022-01861-5 (PMC9630105; doi:10.1038/s41559-022-01861-5)
Supplement: Supplementary file 2 — Reporting Summary [file 41559_2022_1861_MOESM2_ESM.pdf]

nature portfolio

Corresponding author(s): Ana B. Marín-Arroyo; Marco Vidal-Cordasco

Last updated by author(s): 13/07/2022

Reporting Summary

Nature Portfolio wishes to improve the reproducibility of the work that we publish. This form provides structure for consistency and transparency in reporting. For further information on Nature Portfolio policies, see our Editorial Policies and the Editorial Policy Checklist.

Statistics

For all statistical analyses, confirm that the following items are present in the figure legend, table legend, main text, or Methods section.

n/a	Confirmed	
☒	☐	The exact sample size (n) for each experimental group/condition, given as a discrete number and unit of measurement
☒	☐	A statement on whether measurements were taken from distinct samples or whether the same sample was measured repeatedly
☐	☒	The statistical test(s) used AND whether they are one- or two-sided<br>*Only common tests should be described solely by name; describe more complex techniques in the Methods section.*
☐	☒	A description of all covariates tested
☐	☒	A description of any assumptions or corrections, such as tests of normality and adjustment for multiple comparisons
☐	☒	A full description of the statistical parameters including central tendency (e.g. means) or other basic estimates (e.g. regression coefficient) AND variation (e.g. standard deviation) or associated estimates of uncertainty (e.g. confidence intervals)
☐	☒	For null hypothesis testing, the test statistic (e.g. F, t, r) with confidence intervals, effect sizes, degrees of freedom and P value noted<br>*Give P values as exact values whenever suitable.*
☒	☐	For Bayesian analysis, information on the choice of priors and Markov chain Monte Carlo settings
☒	☐	For hierarchical and complex designs, identification of the appropriate level for tests and full reporting of outcomes
☐	☒	Estimates of effect sizes (e.g. Cohen's d, Pearson's r), indicating how they were calculated

Our web collection on statistics for biologists contains articles on many of the points above.

Software and code

Policy information about availability of computer code

Data collection	The LPJ-GUESS v4.0 was used to estimate the Net Primary Productivity. LPJ-GUESS can be obtained on request through Lund University (http://web.nateko.lu.se/lpj-guess)
Data analysis	The R codes used to perform all the analyses reported in the manuscript are available on Github: https://github.com/ERC-Subsilience/Data-and-code-associated-with-Iberia-Neanderthal-ecosystems-productivity_Nature-Ecology-Evolution; Vidal-Cordasco, M., Ocio, D., Hickler, T., & Marín-Arroyo, A. B. (2022). ERC-Subsilience/Data-and-code-associated-with-Iberia-Neanderthal-ecosystems-productivity_Nature-Ecology-Evolution: Data-and-code-associated-with-Iberia-Neanderthal-ecosystems-productivity_Nature-Ecology-Evolution. https://doi.org/10.5281/zenodo.6826921)

For manuscripts utilizing custom algorithms or software that are central to the research but not yet described in published literature, software must be made available to editors and reviewers. We strongly encourage code deposition in a community repository (e.g. GitHub). See the Nature Portfolio guidelines for submitting code & software for further information.

Data

Policy information about availability of data

All manuscripts must include a data availability statement. This statement should provide the following information, where applicable:

- Accession codes, unique identifiers, or web links for publicly available datasets
- A description of any restrictions on data availability
- For clinical datasets or third party data, please ensure that the statement adheres to our policy

All data generated or analysed during this study are available on Github: https://github.com/ERC-Subsilience/Data-and-code-associated-with-Iberia-Neanderthal-ecosystems-productivity_Nature-Ecology-Evolution; Vidal-Cordasco, M., Ocio, D., Hickler, T., & Marín-Arroyo, A. B. (2022). ERC-Subsilience/Data-and-code-

associated-with-Iberia-Neanderthal-ecosystems-productivity_Nature-Ecology-Evolution: Data-and-code-associated-with-Iberia-Neanderthal-ecosystems-productivity_Nature-Ecology-Evolution. https://doi.org/10.5281/zenodo.6826921)

Field-specific reporting

Please select the one below that is the best fit for your research. If you are not sure, read the appropriate sections before making your selection.

☐ Life sciences ☐ Behavioural & social sciences ☒ Ecological, evolutionary & environmental sciences

For a reference copy of the document with all sections, see nature.com/documents/nr-reporting-summary-flat.pdf

Ecological, evolutionary & environmental sciences study design

All studies must disclose on these points even when the disclosure is negative.

Study description	We test whether the temporal and spatial replacement patterns of H. neanderthalensis by H. sapiens in Iberia were affected by alterations in the ecosystem productivity.To this end, we integrated three modelling approaches. First, we built Bayesian age models for each cultural techno-complex in the four biogeographic regions of Iberia. This analysis was performed with the OxCAL4.2 software and the INTCAL20 calibration curve. This chronological assessment was complemented with Optimal Linear Estimation and Summed Calibrated Distribution of dated archaeological assemblages. Second, we used a generalised dynamic vegetation model (LPJ-GUESS v.4) with tested climate inputs from an atmospheric general circulation model to estimate the evolution of Net Primary Productivity (NPP) between 55 and 30 ky BP in each archaeological and paleontological site of the MIS 3. A sensitivity analysis was performed to assess the robustness of the NPP estimations by using the climate inputs obtained from alternative paleoclimatic models. Lastly, we validated a macroecological model against empirical present-day herbivore densities from a broad range of terrestrial ecosystems and used this modelling approach to estimate the herbivore carrying capacity in each stadial and interstadial phase.
Research sample	This study includes the chronometric dates and the herbivore species recovered from 62 archaeological and 7 paleontological sites dated in the MIS 3. We used the climate datasets from the CRU v.4 and the HadCM3B-M2.1 coupled general circulation model to estimate the Net Primary Productivity. The Eurasian Modern Pollen Database v.2 was used to obtain temperature and precipitation transfer functions based on pollen subsets. These predictive functions were applied to the fossil pollen recovered from 93 palynological assemblages from 51 dated archaeological levels from all the biogeographic regions included in this study. The Phylacine dataset was used to obtain the body mass of the herbivore species included in the study. To validate the macroecological model that estimates herbivore abundances, we used data of 516 extant herbivore population densities obtained from the TetraDENSITY database.
Sampling strategy	To our knowledge, this study includes all the archaeological sites of the Middle to Upper Palaeolithic transition in Iberia. A rarefaction test was used to assess the sample size used to reconstruct the paleocommunity composition in each biogeographic region. All data used in this study was compiled from the literature or obtained from the modeling approaches described in the manuscript.
Data collection	Data was collected by Vidal-Cordasco & Marín-Arroyo from the literature.
Timing and spatial scale	Archaeo-paleontological data was collected from the literature between 01/04/2021to 01/11/2021. Data from the LPJ-GUESS model was obtained from 01/06/2021 to 01/11/2021. Regarding the spatial scale, data obtained focused in the Iberian Peninsula.
Data exclusions	We excluded from the age models some archaeological levels with cultural or stratigraphic inconsistencies. Some of these exclusion criteria were pre-established (e.g. only included archaeological levels with Mousterian, Châtelperronian or Aurignacian remains were included); however, after performing the sensitivity tests, we decided to exclude all dates obtained from marine shell remains due to the uncertainties with the reservoir effects. The specific rationale behind each exclusion is discussed in Supplementary Note.
Reproducibility	To verify the findings, all analyses and models were re-run and different sensitivity analyses were performed. The results reported in the manuscript are reproducible with the data and codes available on Github: https://github.com/Marco-Vidal/Data-and-codes-associated-with-Vidal-Cordasco-et-al.-Nature-Ecology-Evolution-. All attempts to reproduce the experiments were successful.
Randomization	Samples were allocated into groups according to: 1) the biogeographic region of each archaeological or paleontological site, 2) the chronological and cultural attributions of each archaeological and paleontological unit, 3) the evolutionary trends of the Net Primary Productivity, and 4) the herbivore guild composition.
Blinding	Blinding was not relevant to this study since the validity of the results are independent of the individuals involved in the study.

Did the study involve field work? ☐ Yes ☒ No

Reporting for specific materials, systems and methods

We require information from authors about some types of materials, experimental systems and methods used in many studies. Here, indicate whether each material, system or method listed is relevant to your study. If you are not sure if a list item applies to your research, read the appropriate section before selecting a response.

Materials & experimental systems

n/a	Involved in the study	
☒	☐	Antibodies
☒	☐	Eukaryotic cell lines
☐	☒	Palaeontology and archaeology
☒	☐	Animals and other organisms
☒	☐	Human research participants
☒	☐	Clinical data
☒	☐	Dual use research of concern

Methods

n/a	Involved in the study	
☒	☐	ChIP-seq
☒	☐	Flow cytometry
☒	☐	MRI-based neuroimaging

Palaeontology and Archaeology

Specimen provenance: All archaeological material in this study were already published in different research papers

Specimen deposition: *Indicate where the specimens have been deposited to permit free access by other researchers.*

Dating methods: Any new date is provided in this study

☒ Tick this box to confirm that the raw and calibrated dates are available in the paper or in Supplementary Information.

Ethics oversight: No ethical approval was required

Note that full information on the approval of the study protocol must also be provided in the manuscript.
